# Predicting Conserved Water Molecules in Binding Sites of Proteins Using Machine Learning Methods and Combining Features

**DOI:** 10.1155/2022/5104464

**Published:** 2022-10-03

**Authors:** Wei Xiao, Juhui Ren, Jutao Hao, Haoyu Wang, Yuhao Li, Liangzhao Lin

**Affiliations:** School of Electronic and Information, Shanghai Dianji University, Shanghai 201306, China

## Abstract

Water molecules play an important role in many biological processes in terms of stabilizing protein structures, assisting protein folding, and improving binding affinity. It is well known that, due to the impacts of various environmental factors, it is difficult to identify the conserved water molecules (CWMs) from free water molecules (FWMs) directly as CWMs are normally deeply embedded in proteins and form strong hydrogen bonds with surrounding polar groups. To circumvent this difficulty, in this work, the abundance of spatial structure information and physicochemical properties of water molecules in proteins inspires us to adopt machine learning methods for identifying the CWMs. Therefore, in this study, a machine learning framework to identify the CWMs in the binding sites of the proteins was presented. First, by analyzing water molecules' physicochemical properties and spatial structure information, six features (i.e., atom density, hydrophilicity, hydrophobicity, solvent-accessible surface area, temperature B-factors, and mobility) were extracted. Those features were further analyzed and combined to reach a higher CWM identification rate. As a result, an optimal feature combination was determined. Based on this optimal combination, seven different machine learning models (including support vector machine (SVM), *K*-nearest neighbor (KNN), decision tree (DT), logistic regression (LR), discriminant analysis (DA), naïve Bayes (NB), and ensemble learning (EL)) were evaluated for their abilities in identifying two categories of water molecules, i.e., CWMs and FWMs. It showed that the EL model was the desired prediction model due to its comprehensive advantages. Furthermore, the presented methodology was validated through a case study of crystal 3skh and extensively compared with Dowser++. The prediction performance showed that the optimal feature combination and the desired EL model in our method could achieve satisfactory prediction accuracy in identifying CWMs from FWMs in the proteins' binding sites.

## 1. Introduction

The research on water molecules in the proteins' binding sites has attracted increasing attention during the past decade [1–6]. The water molecules usually interact with the surrounding atoms by forming the bridging hydrogen bonds, which are important in stabilizing protein structures, and assisting protein folding [2, 3]. Besides, it has been shown that water molecules can improve the binding affinity by increasing the binding energy [5]. In a typical crystal structure, water molecules are normally randomly distributed in the structure. To study the solvent effects of the water molecules, one often adopts the implicit solvent models, which mainly include the Poisson-Boltzmann solvent accessible surface model [7, 8] and the generalized Born solvent accessible surface model [9]. This category can accurately predict and evaluate the binding energy between ligands and targets by calculating the corresponding solvent entropy. However, they cannot reflect the mediating interactions of water molecules between the ligands and the targets, thus affecting the prediction accuracy of the binding modes [10, 11]. The other category is the explicit water models which involve the free energy calculation methods (such as *free energy perturbations* [12] and *thermodynamic integration* [13]) to evaluate the solvent entropy. Although those models can accurately calculate the solvent entropy, they cannot be applied to large-scale drug design due to their computationally demanding nature [3].

Generally speaking, the water molecules in the binding sites of the crystal structures can be divided into two groups, i.e., free water molecules (FWMs) and conserved water molecules (CWMs). The FWMs mean the water molecules that are easily displaced by ligands (often coined as the *displaced water molecules*) and those that are not displaced by ligands but are highly variable in crystal structures [14]. The FWMs not only occupy a certain space in the binding sites but also play an important role in molecular recognition and drug screening. Differently, the CWMs are not displaced by ligands; however, they exist in the overwhelming majority of the crystal structures [14]. In some studies, e.g., [15], the CWMs are determined if the distance between waters in the ligand-free and bound structures is less than 1.2 Å. Moreover, the CWMs that can be deeply buried in proteins and form strong hydrogen bonds with the polar groups of the surrounding proteins are regarded as the *structural water molecules* [16, 17], which have important effects on the structure and function of biomacromolecules (e.g., the catalytic activity of enzymes, the folding and unfolding of proteins, and the conformation of biomacromolecules) [16, 17]. Furthermore, if the CWM is located within 1 Å of another water molecule lying in at least one other homologous protein, then this CWM often refers to as the *consensus water molecule* [18]. Effective identification of the conserved (consensus) water molecules can facilitate ligand designs. For example, if the conserved (consensus) water molecules are known a priori in a protein's binding site, then the ligand design can be improved by including polar atoms at appropriate locations in the ligand to form the hydrogen bonds with the water molecules or to displace them from the binding site [19]. Also, the conserved (consensus) water molecules generally have more neighboring protein atoms, which lead to a more hydrophilic environment, and more hydrogen bonds to the proteins, making the protein atoms less mobile [20]. Additionally, the conserved (consensus) water molecules also play a key role in maintaining and stabilizing the alanine racemase dimer [21] and reducing the flexibility of the *Ω*-loop in class A *β*-lactamases [20]. However, if the influence of the two categories of water molecules on the crystal structures is taken into account, the computational complexity will be greatly increased. Previous studies have found that the CWMs not only stay in a certain space in the binding sites but also directly participate in protein-ligand interactions. Hence, to provide necessary insights for the conformational stability of the macromolecules and to refine the protein-ligand binding and the structural optimization of the ligands, it is necessary to effectively identify the CWMs from the FWMs in the binding sites.

Mainly due to the limitations in X-ray crystallography technology, neutron diffraction, or nuclear magnetic resonance, the position information of water molecules is often inaccurate or not accessible [22]. Therefore, it is difficult to identify the CWMs in the binding sites directly. Currently, four categories of computational methods are mainly used to determine their potential sites in practice [22]. The first category is the simulation-based methods which adopt the *molecular dynamics* (MD) or *Monte Carlo* (MC) simulations to predict the most possible transition status of water molecules in the binding sites. Typical methods include WaterMap [23], Dowser++ [24], and JAWS [25]. For example, JAWS [25] performs with a Metropolis MC scheme to locate the water molecules in the binding sites of a protein or protein-ligand complex. The simulation-based methods can accurately determine the water molecules' sites and obtain their conformation structures. However, this method comes at a cost of high computational complexity; the second category is based on empirical methods [26, 27], which mainly discriminate the water molecules by extracting their significant features (such as the temperature B-factor, solvent-contact surface area, and numbers of protein-water interactions). Hence, the extraction and selection of certain specific features can greatly affect the prediction and migration ability of the models. Differently, the third group, i.e., the knowledge-based methods [28–30], extracts the large-scale experimental data information and summarizes them into “knowledge” which can be used to aid the model prediction. However, to fit the models with high reliability, this category has special requirements on the experimental data's quantities and types. Methods in the fourth category (such as 3D-RISM [31], GIST [32], and GRID [33]) are the grid-based interaction methods in which an array of the grid points are generated first throughout and around the protein, then utilized to calculate the interaction potential [33]. The methods allow many thermodynamic quantities to be calculated in a fraction of the time. However, it is difficult to extract the physical information from the atomic-site density distributions [34]. Over the past few decades, machine learning techniques have been widely applied in solving the problem, such as analysis, classification, and prediction in big data; thus, it is developing rapidly in bioinformatics research [35–37].

Motivated by the above discussions, in this study, a machine learning-based method was presented to predict the CWMs in proteins' binding sites. First, the homologous protein structures of the training dataset were collected and overlapped, and the protein structure pairs with a large root-mean-square deviation (RMSD) value were filtered out. Then, the nearest Euclidean distance (NED) between the water molecule in the binding site and the nearest water molecule in the overlapping protein was calculated. Following the definition in [15], a water molecule with a distance less than and equal to 1.2 Å was defined as the CWM; otherwise, it was defined as the FWM. Next, by analyzing the physicochemical properties and the spatial structure information of each water molecule, six important features (i.e., atom density, hydrophilicity, hydrophobicity, solvent-accessible surface area, temperature B-factors, and mobility) were extracted. Based on this, a feature selection method was adopted to evaluate different feature combinations. As a result, the optimal combination with the best prediction performance was determined. Furthermore, seven machine learning models (i.e., support vector machine (SVM) [38, 39], *K*-nearest neighbor (KNN) [26], decision tree (DT) [40], logistic regression (LR) [41], discriminant analysis (DA) [42], naïve Bayes (NB) [43], and ensemble learning (EL) [44]) were adopted to evaluate their discriminating performance based on the optimal feature combination. Finally, the EL model was investigated as the desired model to identify the CWMs. At last, the performance of the proposed model was evaluated against a test set and further compared with Dowser++. The results revealed that the CWMs could be accurately identified by the proposed feature combination and the machine learning model.

## 2. Methods

### 2.1. Data Collection and Processing

Based on the previous work [22], 2003 pairs of protein-ligand crystal structures with a resolution less than 2.0 Å were collected as the training set. Since the conformational and chemical differences between the homologous protein pairs may affect the position comparison of the water molecules, the overlapping was performed using the Pymol software [45]. Only the homologous protein pairs with the RMSD less than or equal to 2.0 Å were retained.

**Figure 1:**
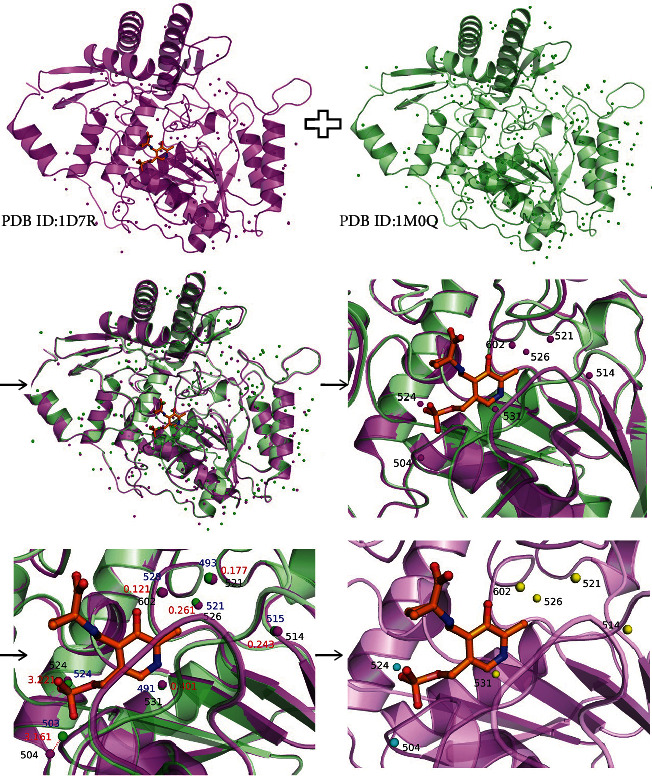
The training process of the dataset. The crystal structure 1D7R is marked in magenta. The crystal structure 1M0Q (i.e., the crystal structure of dialkylglycine decarboxylase complexed with S-1-aminoethanephosphonate [43]) marked in green is the homologous protein of the crystal structure 1D7R. The ligand in the conformation of the crystal structure 1D7R is shown as an orange ball-and-sticks. The magenta and green spheres represent the water molecules in the crystal structures 1D7R and 1M0Q, respectively, while the yellow and cyan ones represent the CWMs and FWMs in the crystal structure 1D7R, respectively. The distances between each of the two water molecules are indicated in red.

Taking 1D7R (i.e., the crystal structure of the complex of 2,2-dialkylglycine decarboxylase with 5PA [46]) as an example (see Figure 1), the detailed training procedure was shown.

- *Align* and *overlap* the homologous crystal structure 1M0Q on 1D7R such that the homologous protein pair was in the same coordinate system;
- *Form* the binding pocket by the protein atoms within a distance of 7.0 Å of any ligand atoms [27, 47] centered on the center point of the ligand in 1D7R;
- In the binding site of 1D7R, there were seven water molecules (magenta spheres). For each water molecule, *calculate* the corresponding NED to the water molecules (green spheres) in 1M0Q;
- *Determine* the CWMs using 1.2 Å [15] as a threshold for the NEDs between the oxygen atoms of the two water molecules in the homologous protein pair. When the NEDs were less than and equal to 1.2 Å, the water molecules in the original crystal structures were regarded as the CWMs (yellow spheres). Otherwise, they were referred to as the FWMs (cyan spheres).

Based on the above data processing steps, the proportion of the number of the FWMs against that of the CWMs in the training set was around 1 : 1.25.

### 2.2. Feature Extraction of Water Molecules

After the training dataset was processed, the extraction of effective features was important for the prediction accuracy of the training model. In this work, by analyzing the physicochemical properties and the spatial structure information of the water molecules in the binding sites, the following six features were extracted to characterize their microenvironments.

(I) *Atom Density*. It was defined as the number of protein atoms within a distance of 3.6 Å of each water molecule [22]. Due to the influence of the morphology of the protein surface, the atom density in the concave groove was normally higher than that in the convex. As a result, the water molecules in the concave grooves tend to interact more with the surrounding polar atoms; thus, they were considered to be highly conservative.
(II) *Atomic Hydrophilicity*. By analyzing the surface-bounded water molecules in 56 high-resolution crystal structures, the individual hydration propensities for each type of amino acid atoms, *h*_*i*_, could be determined by dividing the total number of the water molecules that hydrates an atom by the number of the surface-exposed occurrences [48]. Based on this, the atomic hydrophilicity [18] (Equation (1)) could be calculated by the weighted summation of the propensities from all the atoms (denoted by *N*) within 4 Å of the water molecule, i.e.,
  (1) $$\begin{array}{c} \overset{N}{\underset{i=1}{\sum }}h_{i}e^{{\text{r}}_{i}/d_{0}}, \end{array}$$
  where *r*_*i*_ was the distance between the atom *i* and a water molecule, and *d*_0_ was the distance scale of the interaction.
(III) *Atomic Hydrophobicity*. The hydrophobicity properties of the protein-ligand interfaces varied with proteins, and they reflected the local chemical environment of the water molecule. For the lipophilic score as considered in this work, the corresponding atomic hydrophobicity [18], i.e.,
  (2) $$\begin{array}{c} \overset{N}{\underset{i=1}{\sum }}l_{i}e^{-{\text{r}}_{i}/d_{0}}, \end{array}$$
  where *l*_*i*_ was the carbon propensity of the atom *i*. The other variables were defined the same as in Equation (1).
(IV) *Solvent-Accessible Surface Area (SASA)*. SASA was a measure of the accessibility of water molecules to the outer bulk aqueous environment. As mentioned earlier, the water molecules in the concave grooves on the surface of proteins had fewer contacts with the surrounding aqueous environment as compared to that in the convex. Normally, the NACCESS program [49] was adopted to calculate the SASA of both CWMs and FWMs in the area of concave groove and convex.
(V) *Temperature B-Factors (BFs)*. The BF [27] was often used to measure the atomic stability level in a crystal structure, which was obtained through the square of the average displacement, $\bar{U}$, of an atom, as shown below:
  (3) $$\begin{array}{c} \text{BF}=8\pi^{2}{\bar{U}}^{2}. \end{array}$$
 The BF value reflected the trend of position changing of water molecules in the structure. Generally, the more flexible an atom was, the greater its displacement from its average position. Therefore, water molecules with higher BF values had stronger fluidity than those with lower ones.
(VI) *Mobility*. Instead of staying at a fixed position in a protein, water molecules tend to move around within a certain range. To measure the mobility, *M*, differences of the water molecules, the following equation was adopted to calculate the displacement degree of an atom from its average position:
  (4) $$\begin{array}{c} M=\frac{{\text{BF}}_{\text{i}}/\left(\sum_{i=\text{l}}^{m}{BF}_{\text{i}}/m\right)}{O_{\text{i}}/\left(\sum_{i=\text{l}}^{m}O_{i}/m\right)}, \end{array}$$
  where BF_*i*_ and *O*_*i*_ were the average values of temperature B-factors and the occupancy rates of the *i*th atom, respectively.

The combinations of these features were further evaluated in the following to choose the optimal combination in terms of the CWM identification performance.

### 2.3. Prediction Models

Based on the above six features, the seven most sophisticated machine learning models were adopted to evaluate their performance in terms of CWM identification in the binding sites of the proteins. These models included the SVM, KNN, DT, LR, DA, NB, and EL.

### 2.4. Performance Assessment

In order to quantify the performance of different prediction models, a quality measure was required in order to evaluate the validity of the different feature combinations selected. The performance of different feature combinations and prediction models was further evaluated by considering the following aspects: accuracy (ACC), sensitivity (SN), positive predictive value (PPV), and *F*-score. Mathematically, these parameters were defined in Equations (5)–(8), respectively:

(5) $$\begin{array}{c} \text{ACC}=\frac{\text{TP}+\text{TN}}{\text{TP}+\text{TN}+\text{FP}+\text{FN}}, \end{array}$$

(6) $$\begin{array}{c} \text{SN}=\frac{\text{TP}}{\text{TP}+\text{FN}}, \end{array}$$

(7) $$\begin{array}{c} \text{PPV}=\frac{\text{TP}}{\text{TP}+\text{FP}}, \end{array}$$

(8) $$\begin{array}{c} F‐\text{score}=\frac{2*\text{TP}}{2*\text{TP}+\text{FP}+\text{FN}}, \end{array}$$

where TP and TN meant the numbers of the *true positive* and the *true negative*, respectively, while FP and FN indicated the numbers of the *false positive* and the *false negative*, respectively. More specifically, in this study, they were defined, respectively, as follows:

*True positive*: CWMs that were correctly identified as CWMs.

*False positive*: FWMs that were incorrectly identified as CWMs.

*True negative*: FWMs that were correctly identified as FWMs.

*False negative*: CWMs that were incorrectly identified as FWMs.

Besides, as a useful tool to assess the ability of the prediction model, *the area under the receiver operating characteristic curve* (AUC) was also considered to evaluate their performance. Note that, for all the prediction models, a five-fold cross-validation procedure was adopted to avoid overfitting issues.

## 3. Results and Discussions

### 3.1. Analysis of Features

**Figure 2:**
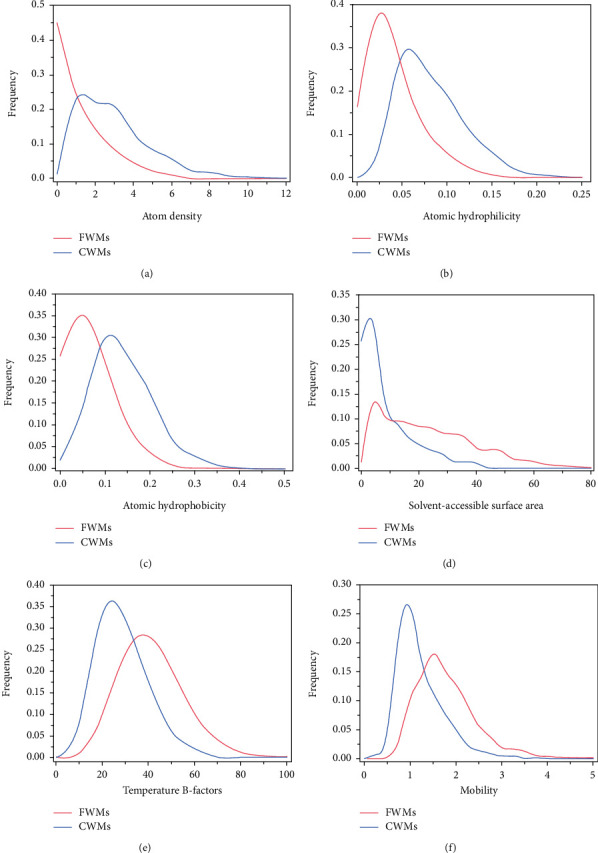
Distributions of different features: (a) atom density; (b) atomic hydrophilicity; (c) atomic hydrophobicity; (d) solvent-accessible surface area; (e) temperature B-factors; (f) mobility. The blue and red curves represent the distributions of the features for the CWMs and FWMs, respectively.

The positions of water molecules in the binding sites of protein were influenced by various factors. To identify the CWMs among them in a more effective way, the distributions of all six features were analyzed in Figure 2. Moreover, the minimum, maximum, and average values of these features for both CWMs and FWMs were listed in Table 1.

From Figure 2, it was obvious that the distributions of all six features of the CWMs and FWMs did not overlap completely. Take the atom density (Figure 2(a)), hydrophilicity (Figure 2(b)), and hydrophobicity (Figure 2(c)) of the CWMs for examples, their *modes* (i.e., the most frequent value in a dataset) were around 1.00, 0.06, and 0.12, respectively, which were all larger than those of the FWMs. This was due to the fact that the CWMs were generally located in the concave grooves of the binding sites, while the FWMs tended to be on the convex surface. In this sense, the atoms around the CWMs were more densely packed than those around the FWMs. As for the B-factors (Figure 2(e)) and the mobility (Figure 2(f)), the corresponding *modes* for the CWMs were around 25 and 1, respectively, which were smaller than those for the FWMs. It was mainly because the CWMs were relatively stable and had less displacement from their average positions. Accordingly, their temperature B-factors and mobility values were relatively small. However, their *modes* of distributions of SASA (Figure 2(d)) for the two categories of water molecules were about 5, which were roughly the same despite the significant frequency differences.

In a summary, the distributions of these six features for the two categories of water molecules overlapped greatly. This made it challenging to identify the CWMs from the FWMs in the proteins' binding sites using one feature alone. Therefore, it motivated us to explore the benefits of the combined features.

### 3.2. Evaluation of Feature Combinations

As shown in Figure 2, it was difficult to identify the key water molecules directly using a single feature alone; we instead considered the combined features to discriminate their final performance. In order to find the desired feature combination in a reasonable way, we evaluated their averaged performance of ACCs, SNs, PPVs, *F*-scores, and AUCs under the seven most commonly used machine learning models (i.e., SVM, KNN, DT, LR, DA, NB, and EL). For example, the averaged ACC, i.e., $\bar{\text{ACC}}$, was defined as the averaged value of all ACC values from all the models, that is,

(9) $$\begin{array}{c} \bar{\text{ACC}}=\frac{\sum_{i}^{n}{ACC}_{i}}{n}, \end{array}$$

where *i* indicated the *i*th machine learning model, *i* = 1, ⋯, *n*. In this study, *n* was chosen as 7. $\bar{\text{SN}}$, $\bar{\text{PPV}}$, $\bar{F‐\text{score}}$, and $\bar{\text{AUC}}$ were defined in a similar way. The results were shown in Table 2 with the detailed table attached in Table S1.

As can be seen from Table 2, in terms of $\bar{\text{ACC}}$, the highest rates of predicting CWMs and FWMs correctly to the total predictions could be achieved by feature combinations No. 1, No. 7, and No. 10 with respective values: 0.725, 0.724, 0.724 (±0.001). These results indicated that combining features in a reasonable way could lead to a better identification ability of the water molecules in the binding sites of proteins. However, a single criterion may cause the loss of the generality. Hence, a comprehensive evaluation of the varied performance resulting from different feature combinations was necessary. To this end, in the following, other commonly used criteria such as $\bar{\text{SN}}$, $\bar{\text{PPV}}$, $\bar{F‐\text{score}}$, and $\bar{\text{AUC}}$ were considered as well. $\bar{\text{SN}}$ was a measure of how effective the prediction model could identify the actual positives (CWMs). It turned out the feature combination No. 58 gave the highest $\bar{\text{SN}}$ value of 0.867. This indicated that the feature of the atom density was important in correctly identifying CWMs. As for $\bar{\text{PPV}}$, which reflected the precision of identifying the CWM, as a result, the combinations No. 1, No. 7, No. 10, and No. 19 achieved the highest values of 0.754 (±0.001). When it came to $\bar{F‐\text{score}}$, which was determined by both PPV and SN (see Equation (8)), the feature combinations No. 1 and No. 10 performed better than other combinations. As for $\bar{\text{AUC}}$, the feature combinations No. 1 and No. 3 gave the best CWM prediction performance with a value of 0.790. Given the above analyses, it was easy to conclude that feature combination No. 1 achieved the best performance in four (i.e., $\bar{\text{ACC}}$, $\bar{\text{PPV}}$, $\bar{F‐\text{score}}$, and $\bar{\text{AUC}}$) out of the five criteria. Naturally, feature combination No. 1 was chosen as the optimal feature combination for the following analysis, which indicated that the water molecules in the binding sites of proteins could be identified more accurately by combining all six features.

### 3.3. Comparison of Prediction Models

Based on the chosen optimal feature combination, the performance of seven commonly used machine learning models in identifying water molecules in the binding sites of proteins was evaluated with results shown in Table 3.

It could be seen from Table 3 that different models were accompanied by their respective performances. Among them, the EL model performed best in four (i.e., ACC, SN, *F*-score, and AUC) out of five criteria, and also, its average performance value was 0.853, which was the highest among all the models. However, in terms of PPV, the DT model posed advantages over other models. After comprehensively considering their performance in terms of different kinds of criteria, it was not hard to conclude that the EL model performed better in identifying the water molecules in the binding sites of the proteins. Therefore, the EL model was selected as the desired prediction model.

### 3.4. Case Study

**Figure 3:**
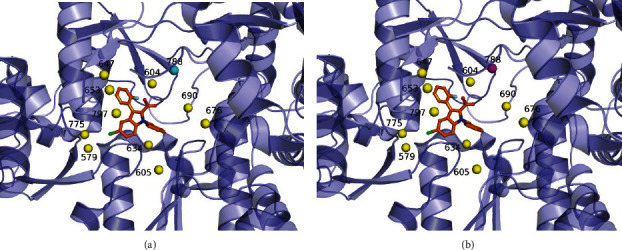
(a) All water molecules in the binding site of Chain B of the crystal structure 3skh, where the yellow and cyan spheres represent the CWMs and FWM, respectively. (b) The predicted results using the EL model, where the yellow spheres represent the correctly identified CWMs, and the magenta sphere represents the mispredicted FWM. Note that the ligands of Chain B in these conformations are shown as the orange ball-and-stick models.

In the following, we took 3skh (i.e., the crystal structure of I. Novel HCV NS5B polymerase inhibitors: discovery of indole 2-carboxylic acids with C3-heterocycles [46]) as a case study, where eleven water molecules were distributed in the binding site of Chain B of the crystal structure 3skh (Figure 3(a)). Among them, the W788 was an FWM (cyan sphere), and the others were CWMs (yellow spheres). By employing the EL model (Figure 3(b)), it could successfully identify all the CWMs but failed on the FWM W788 (magenta sphere). It showed that the prediction model could achieve satisfactory accuracies in predicting the CWMs in the binding site of Chain B of the crystal structure 3skh.

### 3.5. Comparison with Other Methods

In this section, the performance of our method in identifying the CWMs in the proteins' binding sites had been compared with Dowser++ [24] using the same test set [22]. The Dowser++ was based on a semiempirical modification of a program for protein hydration Dowser [50], AutoDock Vina [51], and WaterDock [18]. The six features and the categories of water molecules in the test set were collected in Table S2. Encouragingly, the accuracies of the proposed EL model in predicting the CWMs could reach 77.0% (the detailed predicted results were attached in Table S3), as compared with 59.3% by using Dowser++ (the detailed predicted results were attached in Table S3). These results demonstrated that our method was performing better in predicting the CWMs in the proteins' binding sites.

## 4. Conclusion

In this study, a machine learning-based approach was proposed to identify the CWMs in proteins' binding sites. By analyzing the physicochemical properties and the spatial structure information of the water molecules, six features were extracted to characterize their surrounding microenvironment. A feature selection method was used to train and evaluate different feature combinations, and the optimal combination with better performance was determined. On this basis, seven machine learning models were introduced to evaluate their abilities in identifying the two categories of water molecules. As a result, the EL model with better performance was selected according to various evaluations. A test set was used to verify the effectiveness of the optimal feature combination and the chosen prediction models in our method and compared to Dowser++. The results indicated that our method demonstrated strong performance, which further showed that the desired feature combination and prediction model proposed in this study could effectively identify the CWMs in proteins' binding sites.

## Figures and Tables

**Table 1:** The minimum, maximum, and average values of the features.

Categories of water molecules	Values	Features					
		Atom density	Atomic hydrophilicity	Atomic hydrophobicity	SASA (Å^2^)	BFs	Mobility
FWMs	Min	0.000	0.000	0.000	0.000	0.000	0.000
	Max	7.000	0.147	0.249	84.949	99.930	8.992
	Mean	1.146	0.029	0.049	21.877	36.371	1.673

CWMs	Min	0.000	0.005	0.000	0.000	0.000	0.027
	Max	12.000	0.243	0.505	40.877	94.67	12.289
	Mean	3.033	0.071	0.116	6.683	23.740	1.099

**Table 2:** Averaged performance indices under different feature combinations.

No.	Combination	$\bar{\text{ACC}}$	$\bar{\text{SN}}$	$\bar{\text{PPV}}$	$\bar{F‐\text{score}}$	$\bar{\text{AUC}}$
1	ABCDEF^∗^	**0.725** ^∗∗^	0.809	**0.753**	**0.779**	**0.790**
2	ABCDE	0.717	0.799	0.749	0.773	0.774
3	ABCDF	0.723	0.811	0.751	**0.779**	**0.790**
4	ABCEF	0.723	0.810	0.751	0.778	0.787
5	ABDEF	0.710	0.814	0.733	0.771	0.770
6	ACDEF	0.720	0.815	0.744	0.778	0.780
7	BCDEF	**0.724**	0.807	**0.753**	0.778	0.786
8	ABCD	0.719	0.804	0.749	0.774	0.771
9	ABCE	0.718	0.798	0.751	0.773	0.771
10	ABCF	**0.724**	0.808	**0.754**	**0.780**	0.786
11	ABDE	0.698	0.810	0.722	0.763	0.753
12	ABDF	0.711	0.817	0.734	0.773	0.771
13	ABEF	0.708	0.807	0.735	0.769	0.771
14	ACDE	0.711	0.813	0.735	0.771	0.766
15	ACDF	0.722	0.821	0.744	**0.780**	0.782
16	ACEF	0.719	0.814	0.743	0.777	0.780
17	ADEF	0.703	0.847	0.714	0.775	0.745
18	BCDE	0.718	0.797	0.751	0.773	0.771
19	BCDF	0.723	0.806	**0.753**	0.778	0.787
20	BCEF	0.719	0.799	0.752	0.774	0.783
21	BDEF	0.710	0.811	0.735	0.771	0.773
22	CDEF	0.720	0.812	0.746	0.777	0.780
23	ABC	0.718	0.802	0.749	0.774	0.771
24	ABD	0.699	0.818	0.720	0.765	0.753
25	ABE	0.697	0.810	0.721	0.763	0.754
26	ABF	0.709	0.810	0.735	0.770	0.773
27	ACD	0.712	0.816	0.735	0.773	0.762
28	ACE	0.707	0.807	0.734	0.768	0.760
29	ACF	0.720	0.819	0.743	**0.779**	0.783
30	ADE	0.679	0.827	0.696	0.755	0.705
31	ADF	0.703	0.844	0.715	0.774	0.745
32	AEF	0.701	0.836	0.716	0.771	0.739
33	BCD	0.718	0.799	0.750	0.773	0.773
34	BCE	0.713	0.785	0.751	0.767	0.767
35	BCF	0.716	0.796	0.749	0.771	0.777
36	BDE	0.699	0.811	0.723	0.764	0.754
37	BDF	0.712	0.816	0.735	0.773	0.773
38	BEF	0.705	0.798	0.735	0.765	0.771
39	CDE	0.712	0.808	0.738	0.771	0.767
40	CDF	0.722	0.819	0.745	**0.780**	0.783
41	CEF	0.721	0.817	0.745	**0.779**	0.780
42	DEF	0.703	0.835	0.718	0.772	0.748
43	AB	0.695	0.815	0.717	0.763	0.740
44	AC	0.708	0.820	0.729	0.772	0.760
45	AD	0.673	0.848	0.684	0.757	0.691
46	AE	0.676	0.846	0.688	0.759	0.693
47	AF	0.700	0.847	0.711	0.773	0.739
48	BC	0.707	0.789	0.742	0.764	0.755
49	BD	0.699	0.815	0.721	0.765	0.756
50	BE	0.694	0.802	0.722	0.759	0.750
51	BF	0.702	0.793	0.734	0.761	0.766
52	CD	0.713	0.817	0.736	0.774	0.764
53	CE	0.704	0.795	0.736	0.764	0.759
54	CF	0.713	0.813	0.737	0.772	0.773
55	DE	0.677	0.830	0.693	0.755	0.703
56	DF	0.702	0.840	0.716	0.772	0.746
57	EF	0.697	0.828	0.715	0.767	0.740
58	A	0.664	**0.867**	0.667	0.759	0.662
59	B	0.674	0.796	0.703	0.745	0.729
60	C	0.691	0.811	0.714	0.759	0.738
61	D	0.670	0.842	0.684	0.754	0.693
62	E	0.670	0.838	0.685	0.754	0.690
63	F	0.685	0.842	0.697	0.763	0.726

^∗^A, B, C, D, E, and F represent the features of the atom density, mobility, temperature B-factors, atomic hydrophilicity, atomic hydrophobicity, and SASA, respectively. ^∗∗^In each category, we highlight the values of the best performance in bold. Note that we allow ±0.001 deviations for the values. For example, the $\bar{\text{ACC}}$ values of the best performance are 0.725 and 0.724, respectively.

**Table 3:** Performance comparison of seven machine learning models in identifying water molecules in the binding sites of proteins using the optimal feature combination.

Prediction models	ACC	SN	PPV	*F*-score	AUC	Average performance^∗∗^
SVM	0.809	0.889	0.793	0.838	0.880	0.842
KNN	0.805	0.873	0.797	0.833	0.890	0.840
DT	0.805	0.838	**0.817**	0.827	0.900	0.837
LR	0.795	0.831	0.807	0.819	0.870	0.824
DA	0.793	0.836	0.801	0.818	0.870	0.824
NB	0.798	0.828	0.812	0.820	0.890	0.830
EL	**0.817** ^∗^	**0.890**	0.803	**0.844**	**0.910**	**0.853**

^∗^Bold values indicate the highest performance values. ^∗∗^For each model, the average performance is defined by averaging out all the values from five criteria.

## Data Availability

The datasets supporting the conclusions of this article are included in the additional files.

## Abbreviations

CWMs: Conserved water molecules
SVM: Support vector machine
KNN: *K*-nearest neighbor
DT: Decision tree
LR: Logistic regression
DA: Discriminant analysis
NB: Naïve Bayes
EL: Ensemble learning
FWMs: Free water molecules
RMSD: Root-mean-square deviation
NED: Nearest Euclidean distance
SASA: Solvent-accessible surface area
BFs: Temperature B-factors
ACC: Accuracy
SN: Sensitivity
PPV: Positive predictive value
AUC: Area under the receiver operating characteristic curve.

## Supplementary Materials

Supplementary Materials Additional file 1: average values of the performance parameters of different feature combinations obtained by using seven models. Additional file 2: the results of the six features and the categories of water molecules using the test set. Additional file 3: prediction results obtained using the optimal feature combination and the chosen prediction models in our method. Additional file 4: prediction results obtained using the program Dowser++.
