# Supplementary material for: Predicting Conserved Water Molecules in Binding Sites of Proteins Using Machine Learning Methods and Combining Features
Source: Comput Math Methods Med. 2022 Oct 3;2022:5104464. doi: 10.1155/2022/5104464 (PMC9550495; doi:10.1155/2022/5104464)
Supplement: Supplementary Materials — Additional file 1: average values of the performance parameters of different feature combinations obtained by using seven models. Additional file 2: the results of the six features and the categories of water molecules using the test set. Additional file 3: prediction results obtained using the optimal feature combination and the chosen prediction models in our method. Additional file 4: prediction results obtained using the program Dowser++. [file 5104464.f1.docx]

**Supporting Information**

**Predicting Conserved Water Molecules** **in the Binding Sites of Proteins using Machine Learning Methods and Combining Features**

Wei Xiao^1*^, Juhui Ren^1^, Jutao Hao^1^, Haoyu Wang^1^, Yuhao Li^1^, Liangzhao Lin^1*^

^1^School of Electronic and Information, Shanghai Dianji University, Shanghai 201306, China

* To whom correspondence should be addressed. [xiaow@sdju.edu.cn](mailto:xiaow@sdju.edu.cn); [linlz@sdju.edu.cn](mailto:linlz@sdju.edu.cn)

**Table of contents**

**Table S1**. Average values of the performance parameters of different feature combinations obtained by using seven models.

**Table S2**. The results of the six features and the categories of water molecules using the test set.

**Table S3**. Prediction results obtained using the optimal feature combination and the chosen prediction models in our method.

**Table S4.** Prediction results obtained using the program Dowser++.

**Table S1.** Average values of the performance parameters of different feature combinations obtained by using seven models.

| **NO.** | **Combinations*** | **Models** | **ACC** | **SN** | **PPV** | **F-score** | **AUC** |
| --- | --- | --- | --- | --- | --- | --- | --- |
| 1 | ABCDEF | SVM | 0.809 | 0.889 | 0.793 | 0.838 | 0.880 |
|  |  | KNN | 0.716 | 0.800 | 0.749 | 0.774 | 0.780 |
|  |  | EL | 0.714 | 0.798 | 0.749 | 0.773 | 0.780 |
|  |  | DT | 0.706 | 0.769 | 0.753 | 0.761 | 0.760 |
|  |  | DA | 0.711 | 0.836 | 0.729 | 0.779 | 0.780 |
|  |  | LR | 0.712 | 0.832 | 0.732 | 0.778 | 0.780 |
|  |  | NB | 0.705 | 0.738 | 0.763 | 0.750 | 0.770 |
| 2 | ABCDE | SVM | 0.799 | 0.882 | 0.784 | 0.830 | 0.880 |
|  |  | KNN | 0.710 | 0.793 | 0.747 | 0.769 | 0.760 |
|  |  | EL | 0.710 | 0.779 | 0.753 | 0.766 | 0.760 |
|  |  | DT | 0.702 | 0.762 | 0.751 | 0.757 | 0.750 |
|  |  | DA | 0.702 | 0.824 | 0.724 | 0.771 | 0.760 |
|  |  | LR | 0.704 | 0.823 | 0.727 | 0.772 | 0.760 |
|  |  | NB | 0.692 | 0.730 | 0.756 | 0.743 | 0.750 |
| 3 | ABCDF | SVM | 0.808 | 0.886 | 0.794 | 0.837 | 0.880 |
|  |  | KNN | 0.714 | 0.797 | 0.749 | 0.772 | 0.780 |
|  |  | EL | 0.713 | 0.795 | 0.748 | 0.771 | 0.780 |
|  |  | DT | 0.701 | 0.773 | 0.745 | 0.759 | 0.760 |
|  |  | DA | 0.710 | 0.839 | 0.727 | 0.779 | 0.780 |
|  |  | LR | 0.710 | 0.834 | 0.729 | 0.778 | 0.780 |
|  |  | NB | 0.706 | 0.751 | 0.763 | 0.757 | 0.770 |
| 4 | ABCEF | SVM | 0.806 | 0.874 | 0.797 | 0.834 | 0.880 |
|  |  | KNN | 0.713 | 0.796 | 0.748 | 0.771 | 0.780 |
|  |  | EL | 0.714 | 0.790 | 0.752 | 0.770 | 0.780 |
|  |  | DT | 0.700 | 0.790 | 0.736 | 0.762 | 0.760 |
|  |  | DA | 0.712 | 0.840 | 0.728 | 0.780 | 0.770 |
|  |  | LR | 0.712 | 0.835 | 0.730 | 0.779 | 0.770 |
|  |  | NB | 0.704 | 0.742 | 0.764 | 0.753 | 0.770 |
| 5 | ABDEF | SVM | 0.795 | 0.894 | 0.773 | 0.829 | 0.860 |
|  |  | KNN | 0.703 | 0.809 | 0.731 | 0.768 | 0.760 |
|  |  | EL | 0.702 | 0.801 | 0.734 | 0.766 | 0.760 |
|  |  | DT | 0.684 | 0.789 | 0.719 | 0.752 | 0.740 |
|  |  | DA | 0.699 | 0.839 | 0.715 | 0.772 | 0.760 |
|  |  | LR | 0.699 | 0.832 | 0.718 | 0.771 | 0.760 |
|  |  | NB | 0.685 | 0.734 | 0.743 | 0.739 | 0.750 |
| 6 | ACDEF | SVM | 0.808 | 0.896 | 0.787 | 0.838 | 0.870 |
|  |  | KNN | 0.708 | 0.814 | 0.735 | 0.772 | 0.770 |
|  |  | EL | 0.709 | 0.799 | 0.742 | 0.770 | 0.770 |
|  |  | DT | 0.690 | 0.756 | 0.740 | 0.748 | 0.750 |
|  |  | DA | 0.712 | 0.842 | 0.727 | 0.780 | 0.770 |
|  |  | LR | 0.712 | 0.838 | 0.729 | 0.780 | 0.770 |
|  |  | NB | 0.699 | 0.762 | 0.748 | 0.755 | 0.760 |
| 7 | BCDEF | SVM | 0.806 | 0.871 | 0.798 | 0.833 | 0.870 |
|  |  | KNN | 0.713 | 0.798 | 0.748 | 0.772 | 0.780 |
|  |  | EL | 0.715 | 0.797 | 0.750 | 0.773 | 0.780 |
|  |  | DT | 0.703 | 0.776 | 0.746 | 0.761 | 0.760 |
|  |  | DA | 0.714 | 0.839 | 0.730 | 0.781 | 0.770 |
|  |  | LR | 0.713 | 0.836 | 0.731 | 0.780 | 0.770 |
|  |  | NB | 0.702 | 0.733 | 0.767 | 0.749 | 0.770 |
| 8 | ABCD | SVM | 0.797 | 0.879 | 0.783 | 0.828 | 0.860 |
|  |  | KNN | 0.709 | 0.789 | 0.747 | 0.768 | 0.760 |
|  |  | EL | 0.714 | 0.784 | 0.755 | 0.769 | 0.760 |
|  |  | DT | 0.697 | 0.765 | 0.744 | 0.754 | 0.740 |
|  |  | DA | 0.706 | 0.834 | 0.725 | 0.775 | 0.760 |
|  |  | LR | 0.707 | 0.832 | 0.726 | 0.775 | 0.760 |
|  |  | NB | 0.701 | 0.742 | 0.760 | 0.751 | 0.760 |
| 9 | ABCE | SVM | 0.797 | 0.871 | 0.787 | 0.827 | 0.860 |
|  |  | KNN | 0.710 | 0.785 | 0.749 | 0.767 | 0.760 |
|  |  | EL | 0.712 | 0.775 | 0.757 | 0.766 | 0.760 |
|  |  | DT | 0.705 | 0.756 | 0.759 | 0.757 | 0.740 |
|  |  | DA | 0.702 | 0.830 | 0.722 | 0.772 | 0.760 |
|  |  | LR | 0.702 | 0.828 | 0.723 | 0.772 | 0.760 |
|  |  | NB | 0.697 | 0.738 | 0.758 | 0.748 | 0.760 |
| 10 | ABCF | SVM | 0.807 | 0.870 | 0.801 | 0.834 | 0.880 |
|  |  | KNN | 0.715 | 0.796 | 0.751 | 0.773 | 0.780 |
|  |  | EL | 0.712 | 0.792 | 0.749 | 0.770 | 0.780 |
|  |  | DT | 0.709 | 0.771 | 0.755 | 0.763 | 0.760 |
|  |  | DA | 0.711 | 0.843 | 0.726 | 0.780 | 0.770 |
|  |  | LR | 0.712 | 0.836 | 0.729 | 0.780 | 0.770 |
|  |  | NB | 0.705 | 0.751 | 0.765 | 0.758 | 0.770 |
| 11 | ABDE | SVM | 0.786 | 0.897 | 0.761 | 0.824 | 0.850 |
|  |  | KNN | 0.692 | 0.805 | 0.720 | 0.761 | 0.740 |
|  |  | EL | 0.690 | 0.803 | 0.720 | 0.759 | 0.740 |
|  |  | DT | 0.672 | 0.777 | 0.711 | 0.742 | 0.730 |
|  |  | DA | 0.687 | 0.833 | 0.706 | 0.764 | 0.740 |
|  |  | LR | 0.690 | 0.830 | 0.709 | 0.765 | 0.740 |
|  |  | NB | 0.671 | 0.728 | 0.730 | 0.729 | 0.730 |
| 12 | ABDF | SVM | 0.793 | 0.890 | 0.773 | 0.827 | 0.870 |
|  |  | KNN | 0.703 | 0.809 | 0.732 | 0.768 | 0.760 |
|  |  | EL | 0.702 | 0.808 | 0.731 | 0.768 | 0.760 |
|  |  | DT | 0.687 | 0.785 | 0.724 | 0.753 | 0.740 |
|  |  | DA | 0.698 | 0.843 | 0.713 | 0.772 | 0.760 |
|  |  | LR | 0.700 | 0.836 | 0.717 | 0.772 | 0.760 |
|  |  | NB | 0.693 | 0.748 | 0.748 | 0.748 | 0.750 |
| 13 | ABEF | SVM | 0.793 | 0.872 | 0.781 | 0.824 | 0.870 |
|  |  | KNN | 0.701 | 0.800 | 0.732 | 0.765 | 0.760 |
|  |  | EL | 0.696 | 0.788 | 0.732 | 0.759 | 0.760 |
|  |  | DT | 0.685 | 0.764 | 0.731 | 0.747 | 0.740 |
|  |  | DA | 0.696 | 0.841 | 0.711 | 0.771 | 0.760 |
|  |  | LR | 0.695 | 0.834 | 0.714 | 0.769 | 0.760 |
|  |  | NB | 0.692 | 0.747 | 0.746 | 0.747 | 0.750 |
| 14 | ACDE | SVM | 0.796 | 0.894 | 0.773 | 0.829 | 0.860 |
|  |  | KNN | 0.701 | 0.798 | 0.733 | 0.764 | 0.750 |
|  |  | EL | 0.703 | 0.799 | 0.735 | 0.766 | 0.750 |
|  |  | DT | 0.693 | 0.792 | 0.727 | 0.758 | 0.740 |
|  |  | DA | 0.699 | 0.828 | 0.720 | 0.770 | 0.760 |
|  |  | LR | 0.699 | 0.825 | 0.720 | 0.769 | 0.760 |
|  |  | NB | 0.684 | 0.753 | 0.734 | 0.743 | 0.740 |
| 15 | ACDF | SVM | 0.807 | 0.893 | 0.788 | 0.838 | 0.876 |
|  |  | KNN | 0.710 | 0.809 | 0.739 | 0.772 | 0.770 |
|  |  | EL | 0.712 | 0.809 | 0.741 | 0.773 | 0.770 |
|  |  | DT | 0.696 | 0.778 | 0.737 | 0.757 | 0.760 |
|  |  | DA | 0.711 | 0.844 | 0.726 | 0.781 | 0.770 |
|  |  | LR | 0.711 | 0.839 | 0.728 | 0.780 | 0.770 |
|  |  | NB | 0.704 | 0.772 | 0.748 | 0.760 | 0.760 |
| 16 | ACEF | SVM | 0.806 | 0.883 | 0.792 | 0.835 | 0.870 |
|  |  | KNN | 0.707 | 0.810 | 0.735 | 0.771 | 0.770 |
|  |  | EL | 0.707 | 0.790 | 0.744 | 0.767 | 0.770 |
|  |  | DT | 0.686 | 0.765 | 0.731 | 0.748 | 0.750 |
|  |  | DA | 0.710 | 0.844 | 0.725 | 0.780 | 0.770 |
|  |  | LR | 0.710 | 0.838 | 0.727 | 0.779 | 0.770 |
|  |  | NB | 0.704 | 0.771 | 0.750 | 0.760 | 0.760 |
| 17 | ADEF | SVM | 0.788 | 0.911 | 0.757 | 0.827 | 0.852 |
|  |  | KNN | 0.691 | 0.834 | 0.710 | 0.767 | 0.730 |
|  |  | EL | 0.687 | 0.825 | 0.709 | 0.762 | 0.730 |
|  |  | DT | 0.684 | 0.818 | 0.708 | 0.759 | 0.720 |
|  |  | DA | 0.690 | 0.849 | 0.703 | 0.769 | 0.730 |
|  |  | LR | 0.691 | 0.846 | 0.706 | 0.769 | 0.730 |
|  |  | NB | 0.691 | 0.846 | 0.706 | 0.769 | 0.720 |
| 18 | BCDE | SVM | 0.795 | 0.867 | 0.786 | 0.825 | 0.860 |
|  |  | KNN | 0.711 | 0.788 | 0.750 | 0.768 | 0.760 |
|  |  | EL | 0.711 | 0.779 | 0.754 | 0.766 | 0.760 |
|  |  | DT | 0.703 | 0.763 | 0.752 | 0.758 | 0.740 |
|  |  | DA | 0.705 | 0.827 | 0.726 | 0.773 | 0.760 |
|  |  | LR | 0.705 | 0.827 | 0.726 | 0.773 | 0.760 |
|  |  | NB | 0.699 | 0.728 | 0.766 | 0.746 | 0.760 |
| 19 | BCDF | SVM | 0.803 | 0.866 | 0.798 | 0.830 | 0.878 |
|  |  | KNN | 0.714 | 0.792 | 0.751 | 0.771 | 0.780 |
|  |  | EL | 0.712 | 0.792 | 0.749 | 0.770 | 0.780 |
|  |  | DT | 0.702 | 0.774 | 0.745 | 0.760 | 0.760 |
|  |  | DA | 0.711 | 0.840 | 0.728 | 0.780 | 0.770 |
|  |  | LR | 0.712 | 0.835 | 0.730 | 0.779 | 0.770 |
|  |  | NB | 0.707 | 0.742 | 0.768 | 0.755 | 0.770 |
| 20 | BCEF | SVM | 0.783 | 0.825 | 0.794 | 0.809 | 0.854 |
|  |  | KNN | 0.713 | 0.790 | 0.752 | 0.770 | 0.780 |
|  |  | EL | 0.709 | 0.782 | 0.750 | 0.766 | 0.780 |
|  |  | DT | 0.701 | 0.784 | 0.740 | 0.762 | 0.760 |
|  |  | DA | 0.712 | 0.840 | 0.729 | 0.780 | 0.770 |
|  |  | LR | 0.712 | 0.835 | 0.730 | 0.779 | 0.770 |
|  |  | NB | 0.706 | 0.737 | 0.770 | 0.753 | 0.770 |
| 21 | BDEF | SVM | 0.793 | 0.878 | 0.778 | 0.825 | 0.870 |
|  |  | KNN | 0.703 | 0.805 | 0.733 | 0.767 | 0.760 |
|  |  | EL | 0.701 | 0.803 | 0.732 | 0.766 | 0.760 |
|  |  | DT | 0.685 | 0.785 | 0.721 | 0.752 | 0.740 |
|  |  | DA | 0.698 | 0.840 | 0.714 | 0.772 | 0.760 |
|  |  | LR | 0.698 | 0.833 | 0.717 | 0.770 | 0.760 |
|  |  | NB | 0.691 | 0.734 | 0.752 | 0.743 | 0.760 |
| 22 | CDEF | SVM | 0.804 | 0.877 | 0.793 | 0.833 | 0.870 |
|  |  | KNN | 0.707 | 0.811 | 0.735 | 0.771 | 0.770 |
|  |  | EL | 0.709 | 0.800 | 0.742 | 0.770 | 0.770 |
|  |  | DT | 0.690 | 0.758 | 0.739 | 0.748 | 0.750 |
|  |  | DA | 0.712 | 0.843 | 0.727 | 0.781 | 0.770 |
|  |  | LR | 0.711 | 0.837 | 0.729 | 0.779 | 0.770 |
|  |  | NB | 0.706 | 0.761 | 0.757 | 0.759 | 0.760 |
| 23 | ABC | SVM | 0.794 | 0.869 | 0.783 | 0.824 | 0.860 |
|  |  | KNN | 0.709 | 0.786 | 0.748 | 0.766 | 0.760 |
|  |  | EL | 0.709 | 0.771 | 0.755 | 0.763 | 0.760 |
|  |  | DT | 0.701 | 0.766 | 0.749 | 0.757 | 0.740 |
|  |  | DA | 0.703 | 0.838 | 0.720 | 0.775 | 0.760 |
|  |  | LR | 0.705 | 0.836 | 0.722 | 0.775 | 0.760 |
|  |  | NB | 0.706 | 0.749 | 0.763 | 0.756 | 0.760 |
| 24 | ABD | SVM | 0.780 | 0.887 | 0.758 | 0.817 | 0.850 |
|  |  | KNN | 0.686 | 0.804 | 0.715 | 0.757 | 0.740 |
|  |  | EL | 0.690 | 0.810 | 0.717 | 0.761 | 0.740 |
|  |  | DT | 0.682 | 0.799 | 0.713 | 0.753 | 0.730 |
|  |  | DA | 0.690 | 0.849 | 0.703 | 0.769 | 0.740 |
|  |  | LR | 0.690 | 0.839 | 0.706 | 0.767 | 0.740 |
|  |  | NB | 0.673 | 0.740 | 0.727 | 0.734 | 0.730 |
| 25 | ABE | SVM | 0.777 | 0.878 | 0.759 | 0.814 | 0.850 |
|  |  | KNN | 0.687 | 0.795 | 0.720 | 0.756 | 0.740 |
|  |  | EL | 0.691 | 0.792 | 0.725 | 0.757 | 0.740 |
|  |  | DT | 0.677 | 0.777 | 0.716 | 0.745 | 0.730 |
|  |  | DA | 0.683 | 0.844 | 0.698 | 0.764 | 0.740 |
|  |  | LR | 0.684 | 0.837 | 0.701 | 0.763 | 0.740 |
|  |  | NB | 0.679 | 0.748 | 0.731 | 0.739 | 0.740 |
| 26 | ABF | SVM | 0.790 | 0.866 | 0.780 | 0.821 | 0.872 |
|  |  | KNN | 0.701 | 0.791 | 0.736 | 0.763 | 0.760 |
|  |  | EL | 0.700 | 0.796 | 0.733 | 0.763 | 0.760 |
|  |  | DT | 0.688 | 0.774 | 0.729 | 0.751 | 0.740 |
|  |  | DA | 0.694 | 0.845 | 0.708 | 0.771 | 0.760 |
|  |  | LR | 0.696 | 0.838 | 0.713 | 0.770 | 0.760 |
|  |  | NB | 0.695 | 0.758 | 0.745 | 0.752 | 0.760 |
| 27 | ACD | SVM | 0.791 | 0.888 | 0.772 | 0.826 | 0.854 |
|  |  | KNN | 0.702 | 0.800 | 0.733 | 0.765 | 0.750 |
|  |  | EL | 0.705 | 0.801 | 0.737 | 0.767 | 0.750 |
|  |  | DT | 0.692 | 0.782 | 0.730 | 0.755 | 0.740 |
|  |  | DA | 0.700 | 0.837 | 0.717 | 0.773 | 0.750 |
|  |  | LR | 0.702 | 0.835 | 0.720 | 0.773 | 0.750 |
|  |  | NB | 0.692 | 0.772 | 0.735 | 0.753 | 0.740 |
| 28 | ACE | SVM | 0.789 | 0.881 | 0.772 | 0.823 | 0.852 |
|  |  | KNN | 0.699 | 0.793 | 0.734 | 0.762 | 0.750 |
|  |  | EL | 0.699 | 0.778 | 0.741 | 0.759 | 0.750 |
|  |  | DT | 0.686 | 0.767 | 0.731 | 0.748 | 0.730 |
|  |  | DA | 0.693 | 0.829 | 0.713 | 0.766 | 0.750 |
|  |  | LR | 0.694 | 0.827 | 0.715 | 0.767 | 0.750 |
|  |  | NB | 0.692 | 0.775 | 0.734 | 0.754 | 0.740 |
| 29 | ACF | SVM | 0.805 | 0.876 | 0.795 | 0.834 | 0.882 |
|  |  | KNN | 0.706 | 0.801 | 0.738 | 0.768 | 0.770 |
|  |  | EL | 0.711 | 0.803 | 0.743 | 0.772 | 0.770 |
|  |  | DT | 0.688 | 0.775 | 0.729 | 0.751 | 0.750 |
|  |  | DA | 0.710 | 0.846 | 0.724 | 0.780 | 0.770 |
|  |  | LR | 0.711 | 0.842 | 0.727 | 0.780 | 0.770 |
|  |  | NB | 0.707 | 0.788 | 0.745 | 0.766 | 0.770 |
| 30 | ADE | SVM | 0.766 | 0.926 | 0.727 | 0.814 | 0.814 |
|  |  | KNN | 0.665 | 0.826 | 0.686 | 0.750 | 0.690 |
|  |  | EL | 0.665 | 0.834 | 0.684 | 0.752 | 0.690 |
|  |  | DT | 0.657 | 0.809 | 0.684 | 0.741 | 0.670 |
|  |  | DA | 0.668 | 0.818 | 0.692 | 0.750 | 0.690 |
|  |  | LR | 0.669 | 0.817 | 0.693 | 0.750 | 0.690 |
|  |  | NB | 0.660 | 0.757 | 0.705 | 0.730 | 0.690 |
| 31 | ADF | SVM | 0.788 | 0.911 | 0.758 | 0.828 | 0.856 |
|  |  | KNN | 0.693 | 0.839 | 0.709 | 0.769 | 0.730 |
|  |  | EL | 0.694 | 0.841 | 0.709 | 0.769 | 0.730 |
|  |  | DT | 0.685 | 0.828 | 0.705 | 0.762 | 0.720 |
|  |  | DA | 0.689 | 0.856 | 0.700 | 0.770 | 0.730 |
|  |  | LR | 0.690 | 0.852 | 0.702 | 0.770 | 0.730 |
|  |  | NB | 0.683 | 0.780 | 0.722 | 0.750 | 0.720 |
| 32 | AEF | SVM | 0.787 | 0.895 | 0.763 | 0.824 | 0.854 |
|  |  | KNN | 0.690 | 0.827 | 0.711 | 0.765 | 0.720 |
|  |  | EL | 0.691 | 0.828 | 0.711 | 0.765 | 0.720 |
|  |  | DT | 0.680 | 0.814 | 0.705 | 0.756 | 0.720 |
|  |  | DA | 0.686 | 0.857 | 0.697 | 0.769 | 0.720 |
|  |  | LR | 0.687 | 0.851 | 0.700 | 0.768 | 0.720 |
|  |  | NB | 0.685 | 0.777 | 0.725 | 0.750 | 0.720 |
| 33 | BCD | SVM | 0.786 | 0.853 | 0.782 | 0.816 | 0.858 |
|  |  | KNN | 0.711 | 0.785 | 0.751 | 0.768 | 0.760 |
|  |  | EL | 0.714 | 0.786 | 0.754 | 0.770 | 0.760 |
|  |  | DT | 0.702 | 0.771 | 0.746 | 0.759 | 0.750 |
|  |  | DA | 0.705 | 0.832 | 0.724 | 0.774 | 0.760 |
|  |  | LR | 0.706 | 0.830 | 0.725 | 0.774 | 0.760 |
|  |  | NB | 0.705 | 0.735 | 0.769 | 0.752 | 0.760 |
| 34 | BCE | SVM | 0.768 | 0.810 | 0.781 | 0.795 | 0.830 |
|  |  | KNN | 0.712 | 0.782 | 0.753 | 0.767 | 0.760 |
|  |  | EL | 0.709 | 0.775 | 0.754 | 0.764 | 0.760 |
|  |  | DT | 0.696 | 0.743 | 0.754 | 0.749 | 0.740 |
|  |  | DA | 0.701 | 0.828 | 0.722 | 0.771 | 0.760 |
|  |  | LR | 0.701 | 0.826 | 0.722 | 0.771 | 0.760 |
|  |  | NB | 0.705 | 0.732 | 0.771 | 0.751 | 0.760 |
| 35 | BCF | SVM | 0.757 | 0.803 | 0.770 | 0.786 | 0.822 |
|  |  | KNN | 0.715 | 0.788 | 0.755 | 0.771 | 0.780 |
|  |  | EL | 0.714 | 0.791 | 0.752 | 0.771 | 0.780 |
|  |  | DT | 0.701 | 0.767 | 0.748 | 0.758 | 0.750 |
|  |  | DA | 0.709 | 0.844 | 0.724 | 0.779 | 0.770 |
|  |  | LR | 0.710 | 0.840 | 0.727 | 0.779 | 0.770 |
|  |  | NB | 0.705 | 0.742 | 0.766 | 0.754 | 0.770 |
| 36 | BDE | SVM | 0.777 | 0.877 | 0.760 | 0.814 | 0.850 |
|  |  | KNN | 0.691 | 0.806 | 0.720 | 0.760 | 0.740 |
|  |  | EL | 0.690 | 0.800 | 0.721 | 0.759 | 0.740 |
|  |  | DT | 0.679 | 0.787 | 0.714 | 0.749 | 0.730 |
|  |  | DA | 0.688 | 0.838 | 0.705 | 0.766 | 0.740 |
|  |  | LR | 0.688 | 0.832 | 0.706 | 0.764 | 0.740 |
|  |  | NB | 0.680 | 0.734 | 0.738 | 0.736 | 0.740 |
| 37 | BDF | SVM | 0.790 | 0.875 | 0.776 | 0.822 | 0.872 |
|  |  | KNN | 0.705 | 0.801 | 0.736 | 0.767 | 0.760 |
|  |  | EL | 0.704 | 0.808 | 0.733 | 0.768 | 0.760 |
|  |  | DT | 0.693 | 0.810 | 0.720 | 0.762 | 0.740 |
|  |  | DA | 0.697 | 0.842 | 0.712 | 0.772 | 0.760 |
|  |  | LR | 0.699 | 0.835 | 0.716 | 0.771 | 0.760 |
|  |  | NB | 0.695 | 0.742 | 0.753 | 0.748 | 0.760 |
| 38 | BEF | SVM | 0.771 | 0.813 | 0.784 | 0.798 | 0.850 |
|  |  | KNN | 0.699 | 0.791 | 0.734 | 0.762 | 0.760 |
|  |  | EL | 0.696 | 0.790 | 0.732 | 0.760 | 0.770 |
|  |  | DT | 0.680 | 0.779 | 0.719 | 0.748 | 0.740 |
|  |  | DA | 0.696 | 0.841 | 0.711 | 0.771 | 0.760 |
|  |  | LR | 0.697 | 0.836 | 0.714 | 0.770 | 0.760 |
|  |  | NB | 0.694 | 0.739 | 0.753 | 0.746 | 0.760 |
| 39 | CDE | SVM | 0.787 | 0.871 | 0.774 | 0.819 | 0.856 |
|  |  | KNN | 0.702 | 0.792 | 0.738 | 0.764 | 0.760 |
|  |  | EL | 0.704 | 0.792 | 0.739 | 0.765 | 0.760 |
|  |  | DT | 0.697 | 0.780 | 0.738 | 0.758 | 0.740 |
|  |  | DA | 0.698 | 0.829 | 0.717 | 0.769 | 0.750 |
|  |  | LR | 0.699 | 0.829 | 0.719 | 0.770 | 0.750 |
|  |  | NB | 0.695 | 0.760 | 0.744 | 0.752 | 0.750 |
| 40 | CDF | SVM | 0.801 | 0.868 | 0.794 | 0.829 | 0.874 |
|  |  | KNN | 0.707 | 0.803 | 0.738 | 0.770 | 0.770 |
|  |  | EL | 0.714 | 0.809 | 0.743 | 0.775 | 0.770 |
|  |  | DT | 0.701 | 0.795 | 0.734 | 0.764 | 0.760 |
|  |  | DA | 0.711 | 0.844 | 0.725 | 0.780 | 0.770 |
|  |  | LR | 0.711 | 0.839 | 0.727 | 0.779 | 0.770 |
|  |  | NB | 0.707 | 0.772 | 0.753 | 0.763 | 0.770 |
| 41 | CEF | SVM | 0.782 | 0.822 | 0.794 | 0.808 | 0.860 |
|  |  | KNN | 0.711 | 0.806 | 0.742 | 0.772 | 0.770 |
|  |  | EL | 0.711 | 0.795 | 0.746 | 0.770 | 0.770 |
|  |  | DT | 0.710 | 0.844 | 0.724 | 0.780 | 0.750 |
|  |  | DA | 0.710 | 0.844 | 0.724 | 0.780 | 0.770 |
|  |  | LR | 0.710 | 0.839 | 0.727 | 0.779 | 0.770 |
|  |  | NB | 0.710 | 0.770 | 0.757 | 0.764 | 0.770 |
| 42 | DEF | SVM | 0.787 | 0.898 | 0.762 | 0.825 | 0.854 |
|  |  | KNN | 0.689 | 0.831 | 0.708 | 0.765 | 0.730 |
|  |  | EL | 0.692 | 0.829 | 0.712 | 0.766 | 0.740 |
|  |  | DT | 0.684 | 0.820 | 0.707 | 0.759 | 0.720 |
|  |  | DA | 0.690 | 0.853 | 0.702 | 0.770 | 0.730 |
|  |  | LR | 0.692 | 0.850 | 0.705 | 0.771 | 0.730 |
|  |  | NB | 0.687 | 0.764 | 0.733 | 0.748 | 0.730 |
| 43 | AB | SVM | 0.776 | 0.873 | 0.760 | 0.813 | 0.850 |
|  |  | KNN | 0.685 | 0.795 | 0.717 | 0.754 | 0.720 |
|  |  | EL | 0.687 | 0.800 | 0.718 | 0.757 | 0.710 |
|  |  | DT | 0.677 | 0.775 | 0.717 | 0.745 | 0.700 |
|  |  | DA | 0.678 | 0.853 | 0.690 | 0.763 | 0.730 |
|  |  | LR | 0.680 | 0.844 | 0.695 | 0.762 | 0.730 |
|  |  | NB | 0.681 | 0.767 | 0.724 | 0.745 | 0.740 |
| 44 | AC | SVM | 0.792 | 0.889 | 0.771 | 0.826 | 0.850 |
|  |  | KNN | 0.698 | 0.794 | 0.732 | 0.762 | 0.750 |
|  |  | EL | 0.697 | 0.799 | 0.728 | 0.762 | 0.740 |
|  |  | DT | 0.690 | 0.778 | 0.730 | 0.753 | 0.730 |
|  |  | DA | 0.693 | 0.840 | 0.709 | 0.769 | 0.750 |
|  |  | LR | 0.693 | 0.836 | 0.710 | 0.768 | 0.750 |
|  |  | NB | 0.695 | 0.801 | 0.726 | 0.761 | 0.750 |
| 45 | AD | SVM | 0.764 | 0.950 | 0.717 | 0.817 | 0.800 |
|  |  | KNN | 0.657 | 0.850 | 0.673 | 0.751 | 0.680 |
|  |  | EL | 0.659 | 0.837 | 0.678 | 0.749 | 0.670 |
|  |  | DT | 0.648 | 0.829 | 0.671 | 0.741 | 0.660 |
|  |  | DA | 0.663 | 0.834 | 0.683 | 0.751 | 0.680 |
|  |  | LR | 0.662 | 0.831 | 0.683 | 0.750 | 0.680 |
|  |  | NB | 0.656 | 0.805 | 0.685 | 0.740 | 0.670 |
| 46 | AE | SVM | 0.765 | 0.926 | 0.727 | 0.814 | 0.800 |
|  |  | KNN | 0.662 | 0.840 | 0.679 | 0.751 | 0.670 |
|  |  | EL | 0.662 | 0.855 | 0.676 | 0.755 | 0.670 |
|  |  | DT | 0.661 | 0.828 | 0.682 | 0.748 | 0.670 |
|  |  | DA | 0.660 | 0.838 | 0.678 | 0.750 | 0.680 |
|  |  | LR | 0.661 | 0.834 | 0.681 | 0.750 | 0.680 |
|  |  | NB | 0.661 | 0.800 | 0.691 | 0.742 | 0.680 |
| 47 | AF | SVM | 0.785 | 0.886 | 0.766 | 0.821 | 0.850 |
|  |  | KNN | 0.687 | 0.827 | 0.708 | 0.763 | 0.720 |
|  |  | EL | 0.692 | 0.851 | 0.705 | 0.771 | 0.720 |
|  |  | DT | 0.684 | 0.845 | 0.698 | 0.765 | 0.710 |
|  |  | DA | 0.684 | 0.860 | 0.694 | 0.768 | 0.720 |
|  |  | LR | 0.684 | 0.855 | 0.696 | 0.767 | 0.720 |
|  |  | NB | 0.684 | 0.807 | 0.712 | 0.757 | 0.730 |
| 48 | BC | SVM | 0.729 | 0.789 | 0.741 | 0.764 | 0.778 |
|  |  | KNN | 0.707 | 0.781 | 0.748 | 0.764 | 0.760 |
|  |  | EL | 0.711 | 0.763 | 0.762 | 0.762 | 0.750 |
|  |  | DT | 0.698 | 0.767 | 0.745 | 0.756 | 0.740 |
|  |  | DA | 0.698 | 0.845 | 0.712 | 0.773 | 0.750 |
|  |  | LR | 0.699 | 0.840 | 0.715 | 0.773 | 0.750 |
|  |  | NB | 0.705 | 0.739 | 0.768 | 0.753 | 0.760 |
| 49 | BD | SVM | 0.777 | 0.874 | 0.761 | 0.814 | 0.850 |
|  |  | KNN | 0.688 | 0.802 | 0.718 | 0.758 | 0.740 |
|  |  | EL | 0.690 | 0.803 | 0.719 | 0.759 | 0.740 |
|  |  | DT | 0.676 | 0.790 | 0.710 | 0.748 | 0.730 |
|  |  | DA | 0.687 | 0.846 | 0.701 | 0.767 | 0.740 |
|  |  | LR | 0.688 | 0.839 | 0.704 | 0.766 | 0.740 |
|  |  | NB | 0.684 | 0.748 | 0.737 | 0.743 | 0.750 |
| 50 | BE | SVM | 0.760 | 0.812 | 0.770 | 0.790 | 0.830 |
|  |  | KNN | 0.682 | 0.783 | 0.718 | 0.750 | 0.740 |
|  |  | EL | 0.687 | 0.798 | 0.718 | 0.756 | 0.740 |
|  |  | DT | 0.675 | 0.791 | 0.709 | 0.748 | 0.720 |
|  |  | DA | 0.683 | 0.844 | 0.698 | 0.764 | 0.740 |
|  |  | LR | 0.683 | 0.836 | 0.701 | 0.763 | 0.740 |
|  |  | NB | 0.687 | 0.750 | 0.739 | 0.744 | 0.740 |
| 51 | BF | SVM | 0.746 | 0.789 | 0.763 | 0.775 | 0.820 |
|  |  | KNN | 0.699 | 0.777 | 0.741 | 0.758 | 0.760 |
|  |  | EL | 0.699 | 0.777 | 0.741 | 0.758 | 0.760 |
|  |  | DT | 0.689 | 0.774 | 0.731 | 0.752 | 0.740 |
|  |  | DA | 0.692 | 0.848 | 0.706 | 0.770 | 0.760 |
|  |  | LR | 0.693 | 0.840 | 0.710 | 0.769 | 0.760 |
|  |  | NB | 0.694 | 0.747 | 0.749 | 0.748 | 0.760 |
| 52 | CD | SVM | 0.788 | 0.880 | 0.771 | 0.822 | 0.850 |
|  |  | KNN | 0.700 | 0.794 | 0.735 | 0.763 | 0.750 |
|  |  | EL | 0.704 | 0.800 | 0.737 | 0.767 | 0.750 |
|  |  | DT | 0.697 | 0.790 | 0.733 | 0.761 | 0.740 |
|  |  | DA | 0.700 | 0.837 | 0.718 | 0.773 | 0.750 |
|  |  | LR | 0.700 | 0.834 | 0.718 | 0.772 | 0.750 |
|  |  | NB | 0.701 | 0.784 | 0.740 | 0.761 | 0.760 |
| 53 | CE | SVM | 0.766 | 0.822 | 0.773 | 0.796 | 0.830 |
|  |  | KNN | 0.694 | 0.768 | 0.739 | 0.753 | 0.750 |
|  |  | EL | 0.698 | 0.776 | 0.740 | 0.758 | 0.750 |
|  |  | DT | 0.685 | 0.758 | 0.733 | 0.746 | 0.730 |
|  |  | DA | 0.694 | 0.830 | 0.713 | 0.767 | 0.750 |
|  |  | LR | 0.694 | 0.828 | 0.714 | 0.767 | 0.750 |
|  |  | NB | 0.699 | 0.784 | 0.737 | 0.760 | 0.750 |
| 54 | CF | SVM | 0.756 | 0.813 | 0.763 | 0.787 | 0.820 |
|  |  | KNN | 0.705 | 0.796 | 0.739 | 0.766 | 0.770 |
|  |  | EL | 0.705 | 0.803 | 0.736 | 0.768 | 0.760 |
|  |  | DT | 0.695 | 0.797 | 0.727 | 0.760 | 0.750 |
|  |  | DA | 0.709 | 0.848 | 0.722 | 0.780 | 0.770 |
|  |  | LR | 0.710 | 0.843 | 0.725 | 0.779 | 0.770 |
|  |  | NB | 0.708 | 0.790 | 0.745 | 0.767 | 0.770 |
| 55 | DE | SVM | 0.764 | 0.920 | 0.727 | 0.812 | 0.810 |
|  |  | KNN | 0.660 | 0.828 | 0.681 | 0.748 | 0.690 |
|  |  | EL | 0.666 | 0.841 | 0.683 | 0.754 | 0.680 |
|  |  | DT | 0.652 | 0.796 | 0.684 | 0.736 | 0.670 |
|  |  | DA | 0.667 | 0.825 | 0.689 | 0.751 | 0.690 |
|  |  | LR | 0.666 | 0.822 | 0.689 | 0.750 | 0.690 |
|  |  | NB | 0.662 | 0.776 | 0.701 | 0.736 | 0.690 |
| 56 | DF | SVM | 0.787 | 0.890 | 0.765 | 0.823 | 0.860 |
|  |  | KNN | 0.688 | 0.827 | 0.709 | 0.763 | 0.730 |
|  |  | EL | 0.690 | 0.838 | 0.707 | 0.767 | 0.730 |
|  |  | DT | 0.680 | 0.828 | 0.701 | 0.759 | 0.710 |
|  |  | DA | 0.689 | 0.856 | 0.700 | 0.770 | 0.730 |
|  |  | LR | 0.690 | 0.852 | 0.702 | 0.770 | 0.730 |
|  |  | NB | 0.688 | 0.786 | 0.725 | 0.754 | 0.730 |
| 57 | EF | SVM | 0.766 | 0.840 | 0.764 | 0.800 | 0.840 |
|  |  | KNN | 0.685 | 0.822 | 0.707 | 0.760 | 0.730 |
|  |  | EL | 0.688 | 0.826 | 0.709 | 0.763 | 0.730 |
|  |  | DT | 0.678 | 0.813 | 0.703 | 0.754 | 0.710 |
|  |  | DA | 0.688 | 0.858 | 0.698 | 0.770 | 0.720 |
|  |  | LR | 0.687 | 0.851 | 0.700 | 0.768 | 0.720 |
|  |  | NB | 0.688 | 0.788 | 0.724 | 0.754 | 0.730 |
| 58 | A | SVM | 0.748 | 0.887 | 0.692 | 0.814 | 0.762 |
|  |  | KNN | 0.650 | 0.864 | 0.663 | 0.750 | 0.640 |
|  |  | EL | 0.650 | 0.864 | 0.663 | 0.750 | 0.640 |
|  |  | DT | 0.650 | 0.864 | 0.663 | 0.750 | 0.640 |
|  |  | DA | 0.650 | 0.864 | 0.663 | 0.750 | 0.650 |
|  |  | LR | 0.650 | 0.864 | 0.663 | 0.750 | 0.650 |
|  |  | NB | 0.650 | 0.864 | 0.663 | 0.750 | 0.650 |
| 59 | B | SVM | 0.702 | 0.724 | 0.736 | 0.730 | 0.762 |
|  |  | KNN | 0.669 | 0.744 | 0.721 | 0.732 | 0.720 |
|  |  | EL | 0.669 | 0.794 | 0.702 | 0.745 | 0.720 |
|  |  | DT | 0.664 | 0.796 | 0.695 | 0.742 | 0.710 |
|  |  | DA | 0.668 | 0.867 | 0.678 | 0.761 | 0.730 |
|  |  | LR | 0.671 | 0.853 | 0.684 | 0.759 | 0.730 |
|  |  | NB | 0.674 | 0.794 | 0.707 | 0.748 | 0.730 |
| 60 | C | SVM | 0.697 | 0.790 | 0.703 | 0.744 | 0.746 |
|  |  | KNN | 0.690 | 0.791 | 0.724 | 0.756 | 0.740 |
|  |  | EL | 0.692 | 0.798 | 0.723 | 0.759 | 0.740 |
|  |  | DT | 0.682 | 0.792 | 0.715 | 0.752 | 0.720 |
|  |  | DA | 0.691 | 0.849 | 0.704 | 0.770 | 0.740 |
|  |  | LR | 0.692 | 0.846 | 0.706 | 0.770 | 0.740 |
|  |  | NB | 0.692 | 0.808 | 0.720 | 0.761 | 0.740 |
| 61 | D | SVM | 0.757 | 0.915 | 0.722 | 0.807 | 0.802 |
|  |  | KNN | 0.653 | 0.833 | 0.673 | 0.745 | 0.670 |
|  |  | EL | 0.656 | 0.822 | 0.680 | 0.744 | 0.670 |
|  |  | DT | 0.648 | 0.824 | 0.672 | 0.740 | 0.670 |
|  |  | DA | 0.659 | 0.834 | 0.679 | 0.748 | 0.680 |
|  |  | LR | 0.660 | 0.831 | 0.680 | 0.748 | 0.680 |
|  |  | NB | 0.660 | 0.837 | 0.679 | 0.749 | 0.680 |
| 62 | E | SVM | 0.736 | 0.841 | 0.727 | 0.780 | 0.782 |
|  |  | KNN | 0.658 | 0.850 | 0.673 | 0.751 | 0.670 |
|  |  | EL | 0.660 | 0.850 | 0.675 | 0.753 | 0.670 |
|  |  | DT | 0.660 | 0.835 | 0.680 | 0.749 | 0.670 |
|  |  | DA | 0.659 | 0.830 | 0.680 | 0.748 | 0.680 |
|  |  | LR | 0.660 | 0.828 | 0.681 | 0.748 | 0.680 |
|  |  | NB | 0.657 | 0.833 | 0.678 | 0.747 | 0.680 |
| 63 | F | SVM | 0.725 | 0.817 | 0.724 | 0.768 | 0.780 |
|  |  | KNN | 0.676 | 0.823 | 0.698 | 0.756 | 0.720 |
|  |  | EL | 0.680 | 0.846 | 0.694 | 0.763 | 0.720 |
|  |  | DT | 0.670 | 0.855 | 0.683 | 0.759 | 0.700 |
|  |  | DA | 0.681 | 0.866 | 0.689 | 0.768 | 0.720 |
|  |  | LR | 0.682 | 0.859 | 0.692 | 0.766 | 0.720 |
|  |  | NB | 0.679 | 0.831 | 0.699 | 0.759 | 0.720 |

*A, B, C, D, E, and F respectively represent the feature descriptors of atom density, mobility, temperature B-factors, atomic hydrophilicity, atomic hydrophobicity, and solvent-accessible surface area.

**Table S2.** The results of the six feature descriptors and the categories of water molecules using the test set.

| **NO.** | **Atom density** | **Mobility** | **Temperature B-factors** | **Atomic hydrophilicity** | **Atomic hydrophobicity** | **Solvent-accessible surface area** | **Categories** |
| --- | --- | --- | --- | --- | --- | --- | --- |
| 1 | 0 | 1.155 | 0.497 | 0.020 | 0.025 | 13.154 | 0 |
| 2 | 1 | 1.001 | 0.004 | 0.037 | 0.071 | 3.657 | 1 |
| 3 | 2 | 0.948 | -0.167 | 0.047 | 0.092 | 0.000 | 0 |
| 4 | 2 | 0.928 | -0.232 | 0.077 | 0.029 | 0.093 | 0 |
| 5 | 2 | 0.875 | -0.400 | 0.049 | 0.099 | 0.000 | 0 |
| 6 | 4 | 0.820 | -0.575 | 0.055 | 0.206 | 0.031 | 0 |
| 7 | 3 | 0.691 | -0.991 | 0.090 | 0.028 | 0.618 | 0 |
| 8 | 1 | 1.066 | 0.212 | 0.044 | 0.053 | 0.267 | 0 |
| 9 | 1 | 1.196 | 0.628 | 0.029 | 0.000 | 0.000 | 0 |
| 10 | 5 | 0.826 | -0.558 | 0.125 | 0.056 | 7.981 | 0 |
| 11 | 4 | 0.630 | -1.184 | 0.103 | 0.084 | 4.163 | 1 |
| 12 | 3 | 0.603 | -1.272 | 0.056 | 0.160 | 11.832 | 1 |
| 13 | 1 | 0.784 | -0.691 | 0.043 | 0.072 | 6.105 | 0 |
| 14 | 4 | 0.746 | -0.813 | 0.083 | 0.137 | 0.848 | 1 |
| 15 | 2 | 0.763 | -0.758 | 0.064 | 0.102 | 1.839 | 0 |
| 16 | 6 | 0.739 | -0.834 | 0.155 | 0.225 | 0.000 | 0 |
| 17 | 6 | 0.770 | -0.738 | 0.129 | 0.156 | 0.446 | 0 |
| 18 | 3 | 0.592 | -1.305 | 0.051 | 0.186 | 8.844 | 1 |
| 19 | 7 | 0.604 | -1.267 | 0.103 | 0.253 | 1.656 | 1 |
| 20 | 0 | 1.303 | 0.971 | 0.009 | 0.000 | 0.000 | 0 |
| 21 | 6 | 0.569 | -1.379 | 0.119 | 0.173 | 4.401 | 1 |
| 22 | 2 | 0.544 | -1.459 | 0.032 | 0.126 | 0.354 | 1 |
| 23 | 1 | 0.665 | -1.074 | 0.023 | 0.021 | 0.104 | 1 |
| 24 | 5 | 0.659 | -1.092 | 0.081 | 0.114 | 16.390 | 1 |
| 25 | 4 | 0.697 | -0.970 | 0.091 | 0.136 | 1.131 | 1 |
| 26 | 1 | 1.036 | 0.114 | 0.033 | 0.024 | 0.182 | 0 |
| 27 | 4 | 1.776 | 2.484 | 0.064 | 0.095 | 7.189 | 1 |
| 28 | 4 | 0.543 | -1.463 | 0.094 | 0.178 | 0.000 | 1 |
| 29 | 1 | 0.718 | -0.903 | 0.036 | 0.098 | 0.008 | 1 |
| 30 | 1 | 0.626 | -1.198 | 0.045 | 0.048 | 0.584 | 0 |
| 31 | 0 | 0.720 | -0.898 | 0.004 | 0.052 | 0.071 | 1 |
| 32 | 2 | 0.633 | -1.174 | 0.019 | 0.099 | 0.000 | 1 |
| 33 | 2 | 0.598 | -1.289 | 0.070 | 0.097 | 0.000 | 0 |
| 34 | 1 | 0.813 | -0.599 | 0.036 | 0.096 | 9.176 | 1 |
| 35 | 3 | 0.621 | -1.214 | 0.082 | 0.077 | 0.000 | 1 |
| 36 | 4 | 1.039 | 0.124 | 0.074 | 0.109 | 1.117 | 1 |
| 37 | 7 | 1.250 | 0.801 | 0.116 | 0.160 | 0.000 | 0 |
| 38 | 4 | 0.628 | -0.360 | 0.131 | 0.165 | 5.470 | 0 |
| 39 | 0 | 1.054 | 0.052 | 0.016 | 0.066 | 0.000 | 0 |
| 40 | 3 | 1.148 | 0.143 | 0.071 | 0.105 | 0.387 | 0 |
| 41 | 8 | 1.743 | 0.718 | 0.152 | 0.172 | 0.878 | 0 |
| 42 | 2 | 1.115 | 0.111 | 0.044 | 0.126 | 10.454 | 0 |
| 43 | 1 | 1.808 | 0.782 | 0.058 | 0.053 | 22.670 | 0 |
| 44 | 0 | 1.868 | 0.839 | 0.004 | 0.047 | 9.553 | 0 |
| 45 | 2 | 1.754 | 0.729 | 0.067 | 0.088 | 18.945 | 0 |
| 46 | 0 | 2.115 | 1.078 | 0.000 | 0.000 | 49.703 | 0 |
| 47 | 3 | 0.744 | -0.247 | 0.130 | 0.119 | 0.225 | 0 |
| 48 | 0 | 2.566 | 1.514 | 0.000 | 0.000 | 84.949 | 0 |
| 49 | 0 | 2.309 | 1.266 | 0.014 | 0.021 | 47.387 | 0 |
| 50 | 0 | 2.354 | 1.309 | 0.002 | 0.020 | 40.901 | 0 |
| 51 | 1 | 3.228 | 2.154 | 0.039 | 0.079 | 38.256 | 0 |
| 52 | 1 | 0.866 | -0.567 | 0.030 | 0.065 | 0.000 | 0 |
| 53 | 4 | 0.885 | -0.484 | 0.099 | 0.169 | 1.610 | 0 |
| 54 | 4 | 0.677 | -1.366 | 0.066 | 0.164 | 8.340 | 0 |
| 55 | 2 | 0.928 | -0.306 | 0.065 | 0.104 | 0.000 | 0 |
| 56 | 4 | 0.807 | -0.815 | 0.073 | 0.202 | 0.000 | 0 |
| 57 | 3 | 1.407 | 1.720 | 0.046 | 0.075 | 0.429 | 0 |
| 58 | 1 | 0.945 | -0.234 | 0.033 | 0.072 | 0.000 | 0 |
| 59 | 3 | 0.686 | -1.326 | 0.062 | 0.120 | 2.789 | 0 |
| 60 | 3 | 1.096 | 0.408 | 0.088 | 0.092 | 9.051 | 0 |
| 61 | 4 | 1.207 | 0.876 | 0.099 | 0.126 | 2.917 | 0 |
| 62 | 1 | 1.161 | 0.680 | 0.031 | 0.069 | 5.952 | 0 |
| 63 | 5 | 0.859 | -0.595 | 0.107 | 0.156 | 2.296 | 0 |
| 64 | 1 | 1.274 | 1.159 | 0.062 | 0.025 | 22.312 | 0 |
| 65 | 0 | 1.492 | 2.082 | 0.010 | 0.043 | 32.182 | 0 |
| 66 | 4 | 0.968 | -0.136 | 0.085 | 0.240 | 0.000 | 0 |
| 67 | 5 | 0.971 | -0.122 | 0.104 | 0.073 | 0.787 | 0 |
| 68 | 3 | 1.111 | 0.471 | 0.063 | 0.104 | 6.172 | 0 |
| 69 | 8 | 1.113 | 0.477 | 0.132 | 0.356 | 0.000 | 0 |
| 70 | 0 | 1.468 | 1.977 | 0.007 | 0.000 | 48.302 | 0 |
| 71 | 2 | 0.743 | -1.085 | 0.063 | 0.123 | 2.616 | 0 |
| 72 | 3 | 0.946 | -0.227 | 0.059 | 0.147 | 8.994 | 0 |
| 73 | 3 | 0.548 | -1.250 | 0.057 | 0.118 | 0.000 | 0 |
| 74 | 5 | 0.644 | -0.960 | 0.106 | 0.202 | 1.332 | 0 |
| 75 | 3 | 0.722 | -0.728 | 0.068 | 0.150 | 0.000 | 0 |
| 76 | 2 | 0.730 | -0.702 | 0.072 | 0.076 | 0.000 | 0 |
| 77 | 2 | 0.864 | -0.298 | 0.059 | 0.073 | 0.000 | 0 |
| 78 | 2 | 1.084 | 0.366 | 0.086 | 0.073 | 7.253 | 0 |
| 79 | 4 | 1.388 | 1.281 | 0.090 | 0.133 | 1.117 | 0 |
| 80 | 2 | 1.594 | 1.904 | 0.055 | 0.048 | 25.675 | 0 |
| 81 | 6 | 0.986 | 0.068 | 0.118 | 0.164 | 6.594 | 0 |
| 82 | 0 | 0.618 | -0.495 | 0.034 | 0.060 | 25.115 | 0 |
| 83 | 3 | 0.644 | -0.462 | 0.049 | 0.220 | 5.177 | 0 |
| 84 | 2 | 0.696 | -0.394 | 0.073 | 0.063 | 0.000 | 0 |
| 85 | 7 | 0.391 | -0.790 | 0.138 | 0.215 | 8.807 | 0 |
| 86 | 5 | 0.552 | -0.582 | 0.089 | 0.168 | 0.492 | 0 |
| 87 | 4 | 1.223 | 0.289 | 0.072 | 0.184 | 2.734 | 0 |
| 88 | 2 | 0.673 | -0.425 | 0.093 | 0.151 | 2.744 | 0 |
| 89 | 6 | 1.174 | 0.225 | 0.118 | 0.137 | 0.000 | 0 |
| 90 | 1 | 0.363 | -0.827 | 0.042 | 0.099 | 5.633 | 0 |
| 91 | 5 | 0.709 | -0.378 | 0.110 | 0.100 | 4.734 | 0 |
| 92 | 0 | 1.264 | 0.343 | 0.018 | 0.044 | 33.394 | 0 |
| 93 | 1 | 1.450 | 0.584 | 0.052 | 0.050 | 26.478 | 0 |
| 94 | 2 | 2.073 | 1.392 | 0.016 | 0.090 | 15.334 | 0 |
| 95 | 0 | 0.854 | -0.189 | 0.022 | 0.047 | 14.704 | 0 |
| 96 | 0 | 1.107 | 0.139 | 0.000 | 0.000 | 28.867 | 0 |
| 97 | 1 | 1.004 | 0.005 | 0.017 | 0.000 | 24.369 | 0 |
| 98 | 1 | 0.862 | -0.179 | 0.006 | 0.072 | 26.980 | 0 |
| 99 | 3 | 1.568 | 0.737 | 0.032 | 0.066 | 18.177 | 0 |
| 100 | 0 | 2.455 | 1.888 | 0.004 | 0.045 | 26.807 | 0 |
| 101 | 0 | 2.699 | 2.204 | 0.000 | 0.000 | 38.405 | 0 |
| 102 | 2 | 2.638 | 2.125 | 0.045 | 0.076 | 12.995 | 0 |
| 103 | 0 | 1.777 | 1.008 | 0.000 | 0.000 | 24.198 | 0 |
| 104 | 4 | 1.715 | 0.927 | 0.088 | 0.153 | 19.510 | 0 |
| 105 | 1 | 1.645 | 0.837 | 0.049 | 0.052 | 13.944 | 0 |
| 106 | 1 | 2.276 | 1.656 | 0.020 | 0.026 | 26.052 | 0 |
| 107 | 1 | 1.506 | 0.656 | 0.025 | 0.028 | 27.323 | 0 |
| 108 | 1 | 1.418 | 0.542 | 0.022 | 0.042 | 36.603 | 0 |
| 109 | 0 | 1.636 | 0.825 | 0.000 | 0.000 | 6.915 | 0 |
| 110 | 7 | 0.558 | -0.574 | 0.104 | 0.229 | 0.859 | 0 |
| 111 | 7 | 1.023 | 0.030 | 0.093 | 0.104 | 0.000 | 0 |
| 112 | 0 | 0.645 | -0.461 | 0.038 | 0.064 | 22.137 | 0 |
| 113 | 3 | 0.732 | -0.347 | 0.049 | 0.226 | 4.917 | 0 |
| 114 | 2 | 0.151 | -1.101 | 0.074 | 0.061 | 0.000 | 0 |
| 115 | 7 | 0.333 | -0.866 | 0.149 | 0.216 | 9.164 | 0 |
| 116 | 4 | 0.795 | -0.267 | 0.076 | 0.162 | 0.363 | 0 |
| 117 | 3 | 0.878 | -0.158 | 0.077 | 0.185 | 2.832 | 0 |
| 118 | 2 | 0.327 | -0.873 | 0.074 | 0.133 | 2.991 | 0 |
| 119 | 5 | 0.312 | -0.892 | 0.128 | 0.086 | 0.000 | 0 |
| 120 | 2 | 0.280 | -0.934 | 0.043 | 0.110 | 4.832 | 0 |
| 121 | 4 | 0.598 | -0.522 | 0.115 | 0.121 | 4.567 | 0 |
| 122 | 0 | 1.051 | 0.066 | 0.016 | 0.021 | 23.959 | 0 |
| 123 | 1 | 1.601 | 0.780 | 0.043 | 0.043 | 22.086 | 0 |
| 124 | 0 | 1.654 | 0.848 | 0.020 | 0.117 | 17.704 | 0 |
| 125 | 0 | 0.909 | -0.117 | 0.024 | 0.053 | 18.254 | 0 |
| 126 | 0 | 1.129 | 0.168 | 0.002 | 0.023 | 24.646 | 0 |
| 127 | 1 | 1.471 | 0.611 | 0.019 | 0.000 | 26.984 | 0 |
| 128 | 2 | 1.429 | 0.556 | 0.056 | 0.092 | 1.988 | 0 |
| 129 | 0 | 1.724 | 0.940 | 0.004 | 0.049 | 28.500 | 0 |
| 130 | 1 | 1.674 | 0.875 | 0.029 | 0.051 | 21.727 | 0 |
| 131 | 0 | 2.045 | 1.355 | 0.002 | 0.024 | 34.654 | 0 |
| 132 | 0 | 1.844 | 1.094 | 0.000 | 0.000 | 35.527 | 0 |
| 133 | 1 | 1.445 | 0.577 | 0.045 | 0.069 | 13.867 | 0 |
| 134 | 0 | 1.242 | 0.313 | 0.000 | 0.000 | 22.746 | 0 |
| 135 | 3 | 1.435 | 0.565 | 0.087 | 0.119 | 21.722 | 0 |
| 136 | 0 | 1.225 | 0.292 | 0.000 | 0.000 | 33.593 | 0 |
| 137 | 1 | 3.222 | 2.882 | 0.047 | 0.068 | 15.519 | 0 |
| 138 | 1 | 1.914 | 1.186 | 0.019 | 0.027 | 24.314 | 0 |
| 139 | 1 | 0.699 | -0.390 | 0.019 | 0.027 | 1.205 | 0 |
| 140 | 5 | 0.609 | -0.507 | 0.100 | 0.208 | 0.886 | 0 |
| 141 | 5 | 0.775 | -0.291 | 0.091 | 0.100 | 0.000 | 0 |
| 142 | 2 | 0.461 | -0.700 | 0.043 | 0.084 | 22.184 | 0 |
| 143 | 4 | 0.289 | -0.922 | 0.053 | 0.228 | 4.684 | 0 |
| 144 | 2 | 0.223 | -1.008 | 0.075 | 0.046 | 0.000 | 0 |
| 145 | 7 | 0.366 | -0.822 | 0.154 | 0.215 | 7.477 | 0 |
| 146 | 4 | 0.843 | -0.204 | 0.087 | 0.173 | 0.402 | 0 |
| 147 | 2 | 1.408 | 0.529 | 0.074 | 0.129 | 3.407 | 0 |
| 148 | 2 | 0.362 | -0.827 | 0.074 | 0.151 | 2.860 | 0 |
| 149 | 7 | 0.810 | -0.246 | 0.125 | 0.095 | 0.000 | 0 |
| 150 | 6 | 0.782 | -0.282 | 0.105 | 0.203 | 0.999 | 0 |
| 151 | 1 | 0.265 | -0.954 | 0.048 | 0.146 | 5.556 | 0 |
| 152 | 6 | 0.518 | -0.625 | 0.108 | 0.102 | 4.599 | 0 |
| 153 | 0 | 1.457 | 0.592 | 0.002 | 0.023 | 31.707 | 0 |
| 154 | 1 | 1.470 | 0.609 | 0.058 | 0.025 | 26.927 | 0 |
| 155 | 1 | 0.993 | -0.010 | 0.019 | 0.104 | 14.233 | 0 |
| 156 | 0 | 0.787 | -0.276 | 0.011 | 0.045 | 13.874 | 0 |
| 157 | 0 | 1.616 | 0.800 | 0.000 | 0.000 | 36.003 | 0 |
| 158 | 1 | 1.123 | 0.160 | 0.028 | 0.000 | 22.754 | 0 |
| 159 | 0 | 1.153 | 0.198 | 0.009 | 0.023 | 15.866 | 0 |
| 160 | 1 | 1.089 | 0.116 | 0.004 | 0.056 | 18.778 | 0 |
| 161 | 2 | 1.985 | 1.277 | 0.032 | 0.021 | 9.940 | 0 |
| 162 | 1 | 2.770 | 2.297 | 0.045 | 0.071 | 19.469 | 0 |
| 163 | 0 | 3.155 | 2.795 | 0.002 | 0.026 | 20.603 | 0 |
| 164 | 6 | 1.708 | 0.919 | 0.111 | 0.141 | 17.421 | 0 |
| 165 | 3 | 1.954 | 1.238 | 0.076 | 0.136 | 9.160 | 0 |
| 166 | 0 | 2.536 | 1.993 | 0.002 | 0.024 | 26.212 | 0 |
| 167 | 0 | 3.267 | 2.941 | 0.002 | 0.020 | 35.097 | 0 |
| 168 | 1 | 2.764 | 2.288 | 0.033 | 0.026 | 32.442 | 0 |
| 169 | 6 | 0.944 | -0.072 | 0.111 | 0.212 | 1.167 | 0 |
| 170 | 5 | 1.028 | 0.037 | 0.094 | 0.119 | 0.096 | 0 |
| 171 | 0 | 0.736 | -0.343 | 0.035 | 0.063 | 22.403 | 0 |
| 172 | 4 | 0.394 | -0.786 | 0.047 | 0.219 | 5.088 | 0 |
| 173 | 2 | 0.381 | -0.803 | 0.068 | 0.045 | 0.000 | 0 |
| 174 | 7 | 0.565 | -0.564 | 0.151 | 0.224 | 8.735 | 0 |
| 175 | 3 | 0.389 | -0.793 | 0.083 | 0.157 | 0.528 | 0 |
| 176 | 5 | 1.057 | 0.074 | 0.075 | 0.147 | 3.720 | 0 |
| 177 | 2 | 0.652 | -0.451 | 0.088 | 0.112 | 3.469 | 0 |
| 178 | 6 | 1.226 | 0.293 | 0.145 | 0.080 | 0.000 | 0 |
| 179 | 2 | 0.238 | -0.988 | 0.046 | 0.127 | 4.645 | 0 |
| 180 | 4 | 0.705 | -0.382 | 0.104 | 0.106 | 4.767 | 0 |
| 181 | 0 | 1.197 | 0.255 | 0.013 | 0.019 | 12.267 | 0 |
| 182 | 1 | 2.552 | 2.013 | 0.029 | 0.019 | 26.245 | 0 |
| 183 | 1 | 1.511 | 0.663 | 0.013 | 0.081 | 16.348 | 0 |
| 184 | 0 | 1.309 | 0.401 | 0.024 | 0.052 | 12.515 | 0 |
| 185 | 0 | 1.962 | 1.248 | 0.000 | 0.000 | 39.434 | 0 |
| 186 | 1 | 1.410 | 0.531 | 0.015 | 0.000 | 21.434 | 0 |
| 187 | 0 | 1.358 | 0.465 | 0.010 | 0.026 | 17.497 | 0 |
| 188 | 0 | 1.708 | 0.918 | 0.000 | 0.000 | 25.300 | 0 |
| 189 | 0 | 1.511 | 0.663 | 0.002 | 0.029 | 11.396 | 0 |
| 190 | 3 | 0.952 | -0.062 | 0.034 | 0.067 | 15.631 | 0 |
| 191 | 1 | 3.066 | 2.681 | 0.029 | 0.027 | 25.541 | 0 |
| 192 | 0 | 1.499 | 0.647 | 0.002 | 0.021 | 24.686 | 0 |
| 193 | 5 | 1.484 | 0.628 | 0.100 | 0.132 | 20.960 | 0 |
| 194 | 0 | 1.523 | 0.678 | 0.000 | 0.000 | 25.361 | 0 |
| 195 | 1 | 1.247 | 0.321 | 0.061 | 0.100 | 8.650 | 0 |
| 196 | 1 | 2.285 | 1.666 | 0.022 | 0.029 | 15.027 | 0 |
| 197 | 2 | 2.246 | 1.617 | 0.030 | 0.056 | 25.869 | 0 |
| 198 | 0 | 1.636 | 0.825 | 0.000 | 0.000 | 6.506 | 0 |
| 199 | 1 | 1.891 | 1.631 | 0.020 | 0.047 | 49.258 | 0 |
| 200 | 6 | 0.629 | -0.678 | 0.168 | 0.203 | 0.093 | 0 |
| 201 | 2 | 0.971 | -0.053 | 0.028 | 0.126 | 9.029 | 0 |
| 202 | 3 | 1.790 | 1.446 | 0.040 | 0.107 | 24.174 | 0 |
| 203 | 6 | 0.643 | -0.653 | 0.160 | 0.112 | 5.395 | 0 |
| 204 | 2 | 1.240 | 0.439 | 0.023 | 0.124 | 34.269 | 0 |
| 205 | 2 | 1.457 | 0.837 | 0.040 | 0.109 | 18.507 | 0 |
| 206 | 1 | 2.019 | 1.863 | 0.004 | 0.048 | 40.973 | 0 |
| 207 | 0 | 3.357 | 1.241 | 0.004 | 0.049 | 24.418 | 0 |
| 208 | 9 | 0.708 | -0.534 | 0.110 | 0.254 | 3.356 | 0 |
| 209 | 2 | 1.074 | 0.135 | 0.011 | 0.141 | 17.217 | 0 |
| 210 | 1 | 1.215 | 0.392 | 0.017 | 0.078 | 14.375 | 0 |
| 211 | 4 | 0.642 | -1.042 | 0.101 | 0.099 | 0.167 | 0 |
| 212 | 2 | 0.506 | -1.439 | 0.064 | 0.214 | 23.899 | 0 |
| 213 | 2 | 0.658 | -0.995 | 0.096 | 0.169 | 0.000 | 0 |
| 214 | 3 | 0.545 | -1.327 | 0.099 | 0.102 | 13.527 | 0 |
| 215 | 3 | 1.103 | 0.301 | 0.059 | 0.126 | 13.640 | 0 |
| 216 | 2 | 0.990 | -0.030 | 0.052 | 0.072 | 39.411 | 0 |
| 217 | 3 | 0.659 | -0.475 | 0.075 | 0.080 | 0.129 | 0 |
| 218 | 3 | 0.690 | -0.431 | 0.072 | 0.165 | 6.130 | 0 |
| 219 | 5 | 0.845 | -0.216 | 0.083 | 0.131 | 7.476 | 0 |
| 220 | 3 | 0.542 | -0.638 | 0.065 | 0.147 | 2.737 | 0 |
| 221 | 1 | 1.073 | 0.101 | 0.013 | 0.060 | 0.000 | 0 |
| 222 | 3 | 1.064 | 0.089 | 0.078 | 0.093 | 0.000 | 0 |
| 223 | 3 | 1.548 | 0.763 | 0.124 | 0.074 | 0.549 | 0 |
| 224 | 5 | 0.932 | -0.094 | 0.114 | 0.083 | 1.758 | 0 |
| 225 | 7 | 0.641 | -0.501 | 0.113 | 0.252 | 0.000 | 0 |
| 226 | 1 | 1.120 | 0.168 | 0.058 | 0.068 | 0.000 | 0 |
| 227 | 6 | 0.794 | -0.286 | 0.058 | 0.154 | 6.573 | 0 |
| 228 | 3 | 0.758 | -0.337 | 0.060 | 0.063 | 4.304 | 0 |
| 229 | 3 | 0.688 | -0.434 | 0.067 | 0.190 | 1.991 | 0 |
| 230 | 2 | 1.214 | 0.298 | 0.036 | 0.097 | 0.256 | 0 |
| 231 | 3 | 1.187 | 0.261 | 0.073 | 0.131 | 0.307 | 0 |
| 232 | 1 | 1.681 | 0.949 | 0.051 | 0.048 | 2.659 | 0 |
| 233 | 4 | 1.814 | 1.134 | 0.100 | 0.066 | 1.866 | 0 |
| 234 | 6 | 1.509 | 0.708 | 0.084 | 0.313 | 1.273 | 0 |
| 235 | 7 | 3.645 | 3.684 | 0.113 | 0.242 | 12.546 | 0 |
| 236 | 2 | 1.865 | 1.204 | 0.038 | 0.032 | 26.020 | 0 |
| 237 | 2 | 1.599 | 1.000 | 0.053 | 0.058 | 12.757 | 0 |
| 238 | 3 | 0.878 | -0.310 | 0.062 | 0.157 | 0.162 | 0 |
| 239 | 1 | 0.952 | -0.123 | 0.042 | 0.045 | 0.092 | 0 |
| 240 | 0 | 0.974 | -0.065 | 0.011 | 0.021 | 22.563 | 0 |
| 241 | 2 | 0.688 | -0.790 | 0.055 | 0.110 | 1.473 | 0 |
| 242 | 3 | 0.942 | -0.147 | 0.069 | 0.098 | 0.000 | 0 |
| 243 | 4 | 0.859 | -0.357 | 0.065 | 0.064 | 3.333 | 0 |
| 244 | 2 | 0.908 | -0.233 | 0.071 | 0.044 | 5.758 | 0 |
| 245 | 0 | 0.868 | -0.334 | 0.000 | 0.000 | 1.275 | 0 |
| 246 | 1 | 1.039 | 0.099 | 0.006 | 0.078 | 23.142 | 0 |
| 247 | 4 | 0.587 | -1.047 | 0.048 | 0.167 | 9.608 | 0 |
| 248 | 5 | 1.031 | 0.077 | 0.126 | 0.226 | 0.157 | 0 |
| 249 | 3 | 1.614 | 1.554 | 0.042 | 0.080 | 7.470 | 0 |
| 250 | 1 | 1.065 | 0.165 | 0.034 | 0.043 | 3.425 | 0 |
| 251 | 7 | 0.806 | -0.490 | 0.164 | 0.117 | 1.212 | 0 |
| 252 | 5 | 1.966 | 2.445 | 0.019 | 0.237 | 9.121 | 0 |
| 253 | 2 | 1.462 | 1.170 | 0.036 | 0.087 | 4.182 | 0 |
| 254 | 1 | 0.896 | -0.263 | 0.018 | 0.019 | 8.147 | 0 |
| 255 | 1 | 1.180 | 0.455 | 0.042 | 0.025 | 0.102 | 0 |
| 256 | 2 | 1.661 | 1.673 | 0.029 | 0.086 | 1.175 | 0 |
| 257 | 0 | 1.489 | 1.238 | 0.000 | 0.000 | 2.094 | 0 |
| 258 | 0 | 1.584 | 1.479 | 0.000 | 0.000 | 37.008 | 0 |
| 259 | 3 | 1.573 | 1.451 | 0.016 | 0.205 | 4.660 | 0 |
| 260 | 3 | 1.374 | 0.946 | 0.012 | 0.152 | 20.979 | 0 |
| 261 | 3 | 1.405 | 1.026 | 0.103 | 0.111 | 2.789 | 0 |
| 262 | 0 | 1.371 | 0.938 | 0.000 | 0.000 | 42.841 | 0 |
| 263 | 1 | 1.569 | 1.442 | 0.004 | 0.048 | 2.639 | 0 |
| 264 | 0 | 1.706 | 1.188 | 0.007 | 0.000 | 26.466 | 0 |
| 265 | 0 | 1.639 | 1.074 | 0.000 | 0.000 | 13.471 | 0 |
| 266 | 0 | 1.826 | 1.389 | 0.007 | 0.000 | 19.138 | 0 |
| 267 | 1 | 0.908 | -0.155 | 0.054 | 0.049 | 4.492 | 0 |
| 268 | 0 | 2.019 | 1.714 | 0.000 | 0.000 | 43.700 | 0 |
| 269 | 1 | 0.546 | -0.763 | 0.020 | 0.044 | 0.023 | 0 |
| 270 | 0 | 1.915 | 1.538 | 0.000 | 0.000 | 12.281 | 0 |
| 271 | 2 | 1.735 | 1.237 | 0.045 | 0.097 | 0.000 | 0 |
| 272 | 4 | 1.192 | 0.322 | 0.091 | 0.128 | 7.203 | 0 |
| 273 | 3 | 1.309 | 0.519 | 0.039 | 0.108 | 5.816 | 0 |
| 274 | 0 | 1.791 | 1.329 | 0.000 | 0.000 | 28.884 | 0 |
| 275 | 0 | 0.966 | -0.058 | 0.024 | 0.043 | 6.273 | 0 |
| 276 | 2 | 0.760 | -0.404 | 0.039 | 0.075 | 0.041 | 0 |
| 277 | 2 | 1.930 | 1.563 | 0.022 | 0.053 | 12.037 | 0 |
| 278 | 2 | 1.906 | 1.524 | 0.056 | 0.029 | 0.058 | 0 |
| 279 | 5 | 0.652 | -0.586 | 0.105 | 0.115 | 3.254 | 0 |
| 280 | 1 | 3.525 | 1.282 | 0.028 | 0.000 | 17.156 | 0 |
| 281 | 0 | 0.983 | -0.028 | 0.000 | 0.000 | 1.438 | 0 |
| 282 | 1 | 1.642 | 1.079 | 0.046 | 0.040 | 6.139 | 0 |
| 283 | 0 | 1.517 | 0.870 | 0.011 | 0.000 | 2.618 | 0 |
| 284 | 3 | 2.083 | 1.821 | 0.045 | 0.084 | 4.527 | 0 |
| 285 | 3 | 1.106 | 0.178 | 0.075 | 0.031 | 0.251 | 0 |
| 286 | 2 | 1.202 | 0.339 | 0.043 | 0.129 | 7.251 | 0 |
| 287 | 2 | 1.065 | 0.109 | 0.056 | 0.031 | 5.101 | 0 |
| 288 | 1 | 1.777 | 1.307 | 0.058 | 0.117 | 8.817 | 0 |
| 289 | 3 | 0.607 | -0.661 | 0.083 | 0.089 | 8.098 | 0 |
| 290 | 1 | 0.658 | -0.575 | 0.060 | 0.103 | 0.305 | 0 |
| 291 | 0 | 0.500 | -0.841 | 0.001 | 0.019 | 0.000 | 0 |
| 292 | 1 | 2.013 | 1.703 | 0.023 | 0.090 | 10.226 | 0 |
| 293 | 1 | 1.046 | 0.078 | 0.038 | 0.028 | 0.109 | 0 |
| 294 | 4 | 0.478 | -0.877 | 0.066 | 0.137 | 0.023 | 0 |
| 295 | 2 | 0.931 | -0.116 | 0.046 | 0.168 | 0.000 | 0 |
| 296 | 1 | 1.377 | 0.634 | 0.017 | 0.000 | 11.306 | 0 |
| 297 | 1 | 1.456 | 0.767 | 0.031 | 0.000 | 7.977 | 0 |
| 298 | 1 | 1.519 | 0.873 | 0.025 | 0.000 | 12.270 | 0 |
| 299 | 0 | 1.911 | 1.531 | 0.015 | 0.000 | 18.748 | 0 |
| 300 | 0 | 1.553 | 0.929 | 0.012 | 0.026 | 26.646 | 0 |
| 301 | 1 | 1.280 | 0.470 | 0.023 | 0.020 | 19.672 | 0 |
| 302 | 5 | 0.747 | -0.425 | 0.096 | 0.124 | 2.108 | 0 |
| 303 | 0 | 2.175 | 1.976 | 0.015 | 0.000 | 7.961 | 0 |
| 304 | 0 | 0.998 | -0.003 | 0.000 | 0.000 | 35.557 | 0 |
| 305 | 2 | 0.651 | -0.587 | 0.039 | 0.067 | 8.995 | 0 |
| 306 | 3 | 0.789 | -0.355 | 0.075 | 0.160 | 11.698 | 0 |
| 307 | 2 | 1.016 | 0.241 | 0.064 | 0.102 | 17.065 | 0 |
| 308 | 3 | 1.010 | 0.205 | 0.084 | 0.113 | 1.769 | 0 |
| 309 | 1 | 0.790 | -1.240 | 0.025 | 0.104 | 11.507 | 0 |
| 310 | 4 | 0.859 | -0.783 | 0.087 | 0.052 | 19.550 | 0 |
| 311 | 4 | 1.074 | 0.625 | 0.108 | 0.021 | 15.453 | 0 |
| 312 | 1 | 0.890 | -0.584 | 0.046 | 0.021 | 33.815 | 0 |
| 313 | 1 | 0.815 | -1.071 | 0.025 | 0.075 | 16.005 | 0 |
| 314 | 3 | 0.746 | -1.523 | 0.086 | 0.072 | 1.806 | 0 |
| 315 | 0 | 1.183 | 1.332 | 0.005 | 0.056 | 25.525 | 0 |
| 316 | 1 | 0.711 | -1.755 | 0.048 | 0.071 | 14.404 | 0 |
| 317 | 3 | 0.822 | -1.026 | 0.101 | 0.031 | 0.016 | 0 |
| 318 | 2 | 1.181 | 1.326 | 0.026 | 0.104 | 34.883 | 0 |
| 319 | 8 | 1.106 | 0.829 | 0.151 | 0.237 | 6.928 | 0 |
| 320 | 1 | 1.047 | 0.446 | 0.026 | 0.021 | 43.886 | 0 |
| 321 | 3 | 0.983 | 0.025 | 0.076 | 0.044 | 27.880 | 0 |
| 322 | 0 | 1.294 | 1.533 | 0.000 | 0.000 | 22.237 | 0 |
| 323 | 1 | 1.168 | 0.905 | 0.004 | 0.053 | 2.228 | 0 |
| 324 | 2 | 1.035 | 0.237 | 0.068 | 0.046 | 0.000 | 0 |
| 325 | 2 | 0.844 | -0.722 | 0.065 | 0.050 | 3.136 | 0 |
| 326 | 3 | 0.867 | -0.606 | 0.082 | 0.070 | 0.986 | 0 |
| 327 | 1 | 0.854 | -0.671 | 0.050 | 0.098 | 15.516 | 0 |
| 328 | 2 | 1.369 | 1.911 | 0.061 | 0.032 | 16.130 | 0 |
| 329 | 3 | 0.924 | -0.324 | 0.040 | 0.075 | 20.312 | 0 |
| 330 | 1 | 1.358 | 1.856 | 0.030 | 0.021 | 47.205 | 0 |
| 331 | 0 | 1.088 | 0.504 | 0.000 | 0.000 | 6.697 | 0 |
| 332 | 0 | 1.423 | 2.182 | 0.001 | 0.018 | 46.642 | 0 |
| 333 | 4 | 0.747 | -1.209 | 0.130 | 0.139 | 0.078 | 0 |
| 334 | 5 | 0.724 | -1.324 | 0.153 | 0.218 | 4.250 | 0 |
| 335 | 2 | 0.833 | -0.776 | 0.066 | 0.058 | 3.565 | 0 |
| 336 | 5 | 0.971 | -0.088 | 0.069 | 0.219 | 5.517 | 0 |
| 337 | 1 | 1.184 | 0.986 | 0.043 | 0.119 | 14.094 | 0 |
| 338 | 1 | 1.107 | 0.599 | 0.024 | 0.052 | 1.035 | 0 |
| 339 | 5 | 0.923 | -0.326 | 0.098 | 0.127 | 0.888 | 0 |
| 340 | 2 | 1.012 | 0.120 | 0.046 | 0.095 | 2.284 | 0 |
| 341 | 4 | 0.871 | -0.590 | 0.053 | 0.090 | 1.684 | 0 |
| 342 | 4 | 0.920 | -0.343 | 0.075 | 0.159 | 9.582 | 0 |
| 343 | 2 | 0.903 | -0.426 | 0.074 | 0.135 | 9.085 | 0 |
| 344 | 1 | 0.935 | -0.265 | 0.015 | 0.081 | 1.834 | 0 |
| 345 | 2 | 0.733 | -1.281 | 0.028 | 0.118 | 10.340 | 0 |
| 346 | 0 | 0.836 | -0.765 | 0.000 | 0.000 | 21.421 | 0 |
| 347 | 2 | 0.488 | -0.931 | 0.063 | 0.091 | 0.166 | 0 |
| 348 | 1 | 0.600 | -0.726 | 0.035 | 0.043 | 0.897 | 0 |
| 349 | 3 | 0.666 | -0.607 | 0.068 | 0.000 | 0.000 | 0 |
| 350 | 2 | 0.822 | -0.322 | 0.061 | 0.062 | 6.479 | 0 |
| 351 | 1 | 0.759 | -0.437 | 0.016 | 0.078 | 6.554 | 0 |
| 352 | 1 | 0.599 | -0.729 | 0.048 | 0.092 | 1.855 | 0 |
| 353 | 7 | 0.901 | -0.179 | 0.134 | 0.153 | 13.051 | 0 |
| 354 | 3 | 1.427 | 0.780 | 0.077 | 0.129 | 19.647 | 0 |
| 355 | 1 | 1.304 | 0.556 | 0.047 | 0.024 | 1.932 | 0 |
| 356 | 6 | 1.172 | 0.315 | 0.094 | 0.135 | 0.000 | 0 |
| 357 | 2 | 0.855 | -0.263 | 0.069 | 0.044 | 18.945 | 0 |
| 358 | 2 | 2.073 | 1.955 | 0.049 | 0.087 | 4.426 | 0 |
| 359 | 2 | 1.362 | 0.660 | 0.023 | 0.059 | 6.025 | 0 |
| 360 | 2 | 2.109 | 2.022 | 0.028 | 0.075 | 12.539 | 0 |
| 361 | 1 | 1.861 | 1.569 | 0.023 | 0.062 | 21.720 | 0 |
| 362 | 2 | 2.354 | 2.467 | 0.028 | 0.091 | 41.004 | 0 |
| 363 | 0 | 0.712 | -0.522 | 0.008 | 0.094 | 0.076 | 0 |
| 364 | 2 | 0.432 | -1.033 | 0.064 | 0.071 | 0.000 | 0 |
| 365 | 3 | 0.700 | -0.544 | 0.067 | 0.000 | 0.000 | 0 |
| 366 | 8 | 0.651 | -0.634 | 0.144 | 0.184 | 7.050 | 0 |
| 367 | 2 | 0.903 | -0.175 | 0.065 | 0.022 | 2.875 | 0 |
| 368 | 3 | 0.958 | -0.074 | 0.045 | 0.063 | 1.760 | 0 |
| 369 | 3 | 1.096 | 0.176 | 0.053 | 0.077 | 0.000 | 0 |
| 370 | 2 | 1.071 | 0.132 | 0.035 | 0.036 | 11.760 | 0 |
| 371 | 0 | 1.228 | 0.417 | 0.012 | 0.030 | 0.668 | 0 |
| 372 | 2 | 1.004 | 0.010 | 0.046 | 0.073 | 6.056 | 0 |
| 373 | 2 | 0.857 | -0.259 | 0.051 | 0.061 | 0.000 | 0 |
| 374 | 2 | 1.188 | 0.343 | 0.073 | 0.052 | 11.670 | 0 |
| 375 | 1 | 0.852 | -0.269 | 0.047 | 0.072 | 1.698 | 0 |
| 376 | 4 | 1.004 | 0.010 | 0.080 | 0.117 | 0.043 | 0 |
| 377 | 3 | 1.284 | 0.519 | 0.089 | 0.052 | 8.448 | 0 |
| 378 | 1 | 1.692 | 1.261 | 0.028 | 0.114 | 14.600 | 0 |
| 379 | 2 | 1.027 | 0.050 | 0.016 | 0.200 | 0.000 | 0 |
| 380 | 2 | 1.708 | 1.292 | 0.077 | 0.094 | 15.851 | 0 |
| 381 | 1 | 1.957 | 1.744 | 0.026 | 0.079 | 3.617 | 0 |
| 382 | 2 | 1.638 | 1.164 | 0.052 | 0.064 | 22.794 | 0 |
| 383 | 0 | 2.216 | 2.216 | 0.000 | 0.000 | 15.969 | 0 |
| 384 | 1 | 1.778 | 1.418 | 0.030 | 0.022 | 19.654 | 0 |
| 385 | 2 | 1.090 | 0.340 | 0.038 | 0.000 | 9.904 | 0 |
| 386 | 2 | 0.586 | -1.570 | 0.057 | 0.137 | 12.527 | 0 |
| 387 | 4 | 0.662 | -1.280 | 0.081 | 0.102 | 0.053 | 0 |
| 388 | 5 | 0.739 | -0.989 | 0.088 | 0.118 | 4.276 | 0 |
| 389 | 4 | 0.705 | -1.116 | 0.049 | 0.169 | 0.000 | 0 |
| 390 | 0 | 0.741 | -0.983 | 0.002 | 0.022 | 45.624 | 0 |
| 391 | 3 | 0.783 | -0.823 | 0.054 | 0.104 | 2.484 | 0 |
| 392 | 3 | 1.281 | 1.066 | 0.042 | 0.084 | 28.511 | 0 |
| 393 | 0 | 0.711 | -1.095 | 0.015 | 0.028 | 0.000 | 0 |
| 394 | 0 | 0.826 | -0.657 | 0.011 | 0.020 | 6.547 | 0 |
| 395 | 2 | 1.101 | 0.381 | 0.028 | 0.102 | 12.792 | 0 |
| 396 | 3 | 1.170 | 0.242 | 0.071 | 0.043 | 0.921 | 0 |
| 397 | 2 | 1.200 | 0.286 | 0.042 | 0.069 | 3.771 | 0 |
| 398 | 0 | 1.070 | 0.100 | 0.000 | 0.000 | 0.912 | 0 |
| 399 | 5 | 1.461 | 0.657 | 0.105 | 0.109 | 4.678 | 0 |
| 400 | 3 | 1.451 | 0.643 | 0.089 | 0.135 | 13.204 | 0 |
| 401 | 1 | 1.341 | 0.486 | 0.024 | 0.053 | 9.729 | 0 |
| 402 | 0 | 1.312 | 0.445 | 0.000 | 0.000 | 14.374 | 0 |
| 403 | 1 | 1.429 | 0.611 | 0.022 | 0.000 | 2.549 | 0 |
| 404 | 6 | 1.319 | 0.455 | 0.136 | 0.172 | 1.127 | 0 |
| 405 | 2 | 1.427 | 0.608 | 0.029 | 0.032 | 21.099 | 0 |
| 406 | 0 | 1.350 | 0.499 | 0.000 | 0.000 | 0.081 | 0 |
| 407 | 1 | 1.336 | 0.479 | 0.008 | 0.098 | 13.956 | 0 |
| 408 | 1 | 1.590 | 0.840 | 0.040 | 0.078 | 1.720 | 0 |
| 409 | 0 | 1.513 | 0.731 | 0.027 | 0.022 | 0.000 | 0 |
| 410 | 0 | 2.218 | 1.735 | 0.000 | 0.000 | 51.581 | 0 |
| 411 | 2 | 2.388 | 1.978 | 0.042 | 0.109 | 6.367 | 0 |
| 412 | 0 | 2.528 | 2.177 | 0.002 | 0.025 | 22.340 | 0 |
| 413 | 2 | 2.050 | 1.497 | 0.044 | 0.073 | 0.000 | 0 |
| 414 | 1 | 1.326 | 0.735 | 0.074 | 0.073 | 5.749 | 0 |
| 415 | 1 | 1.346 | 0.780 | 0.040 | 0.044 | 26.870 | 0 |
| 416 | 1 | 1.270 | 0.607 | 0.005 | 0.022 | 30.206 | 0 |
| 417 | 0 | 1.324 | 0.730 | 0.000 | 0.000 | 62.193 | 0 |
| 418 | 4 | 1.569 | 1.280 | 0.072 | 0.098 | 0.357 | 0 |
| 419 | 1 | 1.527 | 1.187 | 0.039 | 0.000 | 33.857 | 0 |
| 420 | 1 | 1.402 | 0.905 | 0.003 | 0.035 | 32.346 | 0 |
| 421 | 0 | 1.520 | 1.171 | 0.000 | 0.000 | 24.223 | 0 |
| 422 | 2 | 1.299 | 0.672 | 0.070 | 0.061 | 41.016 | 0 |
| 423 | 3 | 1.340 | 0.766 | 0.032 | 0.101 | 0.000 | 0 |
| 424 | 3 | 1.465 | 1.046 | 0.111 | 0.085 | 25.264 | 0 |
| 425 | 2 | 1.448 | 1.008 | 0.061 | 0.068 | 27.853 | 0 |
| 426 | 2 | 1.610 | 1.372 | 0.034 | 0.029 | 30.595 | 0 |
| 427 | 2 | 1.597 | 1.343 | 0.034 | 0.082 | 14.771 | 0 |
| 428 | 0 | 1.588 | 1.323 | 0.004 | 0.048 | 34.592 | 0 |
| 429 | 2 | 1.160 | 0.359 | 0.012 | 0.044 | 28.512 | 0 |
| 430 | 3 | 0.849 | -1.102 | 0.067 | 0.154 | 10.634 | 0 |
| 431 | 2 | 0.815 | -1.349 | 0.063 | 0.065 | 40.392 | 0 |
| 432 | 7 | 0.993 | -0.049 | 0.056 | 0.275 | 4.870 | 0 |
| 433 | 0 | 0.809 | -1.394 | 0.002 | 0.027 | 33.441 | 0 |
| 434 | 2 | 0.982 | -0.133 | 0.055 | 0.071 | 5.572 | 0 |
| 435 | 8 | 0.500 | -0.420 | 0.126 | 0.192 | 1.716 | 0 |
| 436 | 4 | 0.596 | -0.388 | 0.117 | 0.095 | 4.425 | 0 |
| 437 | 2 | 1.247 | 0.188 | 0.031 | 0.155 | 8.072 | 0 |
| 438 | 1 | 2.310 | 0.469 | 0.006 | 0.070 | 3.237 | 0 |
| 439 | 0 | 0.789 | -0.374 | 0.021 | 0.119 | 0.000 | 0 |
| 440 | 1 | 2.482 | 0.569 | 0.045 | 0.098 | 15.587 | 0 |
| 441 | 2 | 1.573 | 0.324 | 0.070 | 0.095 | 16.748 | 0 |
| 442 | 1 | 3.047 | 1.572 | 0.039 | 0.026 | 26.779 | 0 |
| 443 | 5 | 0.793 | -0.160 | 0.045 | 0.162 | 0.000 | 0 |
| 444 | 3 | 0.781 | -0.171 | 0.067 | 0.099 | 3.508 | 0 |
| 445 | 3 | 2.605 | 1.103 | 0.036 | 0.093 | 11.631 | 0 |
| 446 | 3 | 2.650 | 1.490 | 0.047 | 0.070 | 24.900 | 0 |
| 447 | 3 | 2.598 | 1.444 | 0.025 | 0.072 | 31.483 | 0 |
| 448 | 0 | 1.353 | 0.338 | 0.013 | 0.038 | 21.879 | 0 |
| 449 | 0 | 0.936 | -0.199 | 0.003 | 0.044 | 13.071 | 0 |
| 450 | 1 | 1.441 | 0.416 | 0.035 | 0.065 | 31.416 | 0 |
| 451 | 1 | 1.381 | 0.363 | 0.039 | 0.045 | 27.580 | 0 |
| 452 | 2 | 1.738 | 0.448 | 0.042 | 0.043 | 29.017 | 0 |
| 453 | 1 | 1.101 | 0.114 | 0.037 | 0.050 | 9.345 | 0 |
| 454 | 0 | 1.073 | -0.149 | 0.010 | 0.023 | 10.689 | 0 |
| 455 | 3 | 1.356 | 0.039 | 0.043 | 0.081 | 22.131 | 0 |
| 456 | 0 | 1.559 | 0.313 | 0.003 | 0.041 | 31.299 | 0 |
| 457 | 1 | 1.851 | 0.533 | 0.004 | 0.046 | 34.316 | 0 |
| 458 | 1 | 2.672 | 1.391 | 0.012 | 0.038 | 43.505 | 0 |
| 459 | 1 | 2.478 | 1.337 | 0.013 | 0.058 | 28.350 | 0 |
| 460 | 0 | 1.851 | 0.533 | 0.012 | 0.000 | 19.590 | 0 |
| 461 | 0 | 1.405 | 0.384 | 0.002 | 0.020 | 11.321 | 0 |
| 462 | 1 | 1.580 | 0.469 | 0.049 | 0.068 | 22.570 | 0 |
| 463 | 1 | 2.314 | 1.191 | 0.004 | 0.052 | 38.865 | 0 |
| 464 | 2 | 1.780 | 0.164 | 0.073 | 0.071 | 25.430 | 0 |
| 465 | 0 | 2.054 | 0.960 | 0.000 | 0.000 | 70.751 | 0 |
| 466 | 1 | 2.349 | 1.014 | 0.048 | 0.051 | 15.199 | 0 |
| 467 | 1 | 1.513 | 0.480 | 0.029 | 0.000 | 23.784 | 0 |
| 468 | 2 | 1.481 | 0.452 | 0.035 | 0.000 | 32.059 | 0 |
| 469 | 1 | 1.545 | -0.178 | 0.005 | 0.057 | 30.719 | 0 |
| 470 | 3 | 1.766 | 0.469 | 0.067 | 0.050 | 25.587 | 0 |
| 471 | 0 | 1.986 | 0.900 | 0.002 | 0.028 | 23.698 | 0 |
| 472 | 1 | 2.478 | 1.337 | 0.025 | 0.148 | 29.392 | 0 |
| 473 | 2 | 3.151 | 1.935 | 0.056 | 0.111 | 27.880 | 0 |
| 474 | 1 | 2.538 | 1.391 | 0.018 | 0.075 | 23.574 | 0 |
| 475 | 0 | 2.529 | 0.484 | 0.016 | 0.000 | 44.226 | 0 |
| 476 | 2 | 4.658 | 2.653 | 0.035 | 0.032 | 29.871 | 0 |
| 477 | 3 | 3.203 | 1.554 | 0.026 | 0.123 | 20.910 | 0 |
| 478 | 1 | 3.755 | 2.472 | 0.010 | 0.041 | 32.052 | 0 |
| 479 | 0 | 2.941 | 0.573 | 0.011 | 0.000 | 39.886 | 0 |
| 480 | 0 | 3.138 | 1.366 | 0.002 | 0.019 | 34.961 | 0 |
| 481 | 2 | 3.373 | 0.334 | 0.055 | 0.074 | 26.913 | 0 |
| 482 | 2 | 2.946 | 0.967 | 0.007 | 0.091 | 28.077 | 0 |
| 483 | 1 | 2.907 | 1.718 | 0.007 | 0.085 | 16.295 | 0 |

*A category of 0 represents the free water molecules and a category of 1 represents the conserved water molecules.

**Table S3.** Prediction results obtained using the optimal feature combination and the chosen prediction models in our method.

| **Model** | **ACC** | **SN** | **PPV** | **F-score** | **AUC** |
| --- | --- | --- | --- | --- | --- |
| EL | 0.770 | 0.787 | 0.751 | 0.768 | 0.870 |

**Table S4**. Prediction results obtained using the program Dowser++.

| **no.** | **PDB code** | **Sites of the experimentally determined water molecules** | **Categories** | **Nearest predicted sites to the experimentally determined water molecules** | **RMSDs**  **(Å)** | **Predicted Categories** |
| --- | --- | --- | --- | --- | --- | --- |
|  |  |  |  |  |  |  |
| 1 | 1a53 | (-4.113,-20.798,26.289 | 1 | (-4.106, -20.854, 26.226) | 0.085 | 1 |
| 2 | 1a53 | (-5.090,-24.427,25.070) | 1 | (-4.742,-24.475,25.188) | 0.371 | 1 |
| 3 | 1a53 | (-12.053,-14.577,34.138) | 1 | (-10.752,-14.65,34.341) | 1.319 | 1 |
| 4 | 1a53 | (-10.140,-13.538,35.715) | 1 | (-10.752,-14.65,34.341) | 1.871 | 1 |
| 5 | 1a53 | (-5.674,-17.468,42.698) | 1 | (-5.276,-16.965,43.288) | 0.872 | 1 |
| 6 | 1a53 | (-7.689,-24.636,23.617) | 0 | (-5.127,-23.938,24.438) | 2.779 | 0 |
| 7 | 1a53 | (-9.343,-24.333,26.023) | 0 | (-9.375,-24.937,29.126) | 3.161 | 0 |
| 8 | 1a53 | (-9.497,-21.864,29.218) | 0 | (-9.753,-22.621,31.035) | 1.985 | 1 |
| 9 | 1a53 | (-10.189,-26.048,29.270) | 1 | (-9.968,-25.212,29.883) | 1.060 | 1 |
| 10 | 1a53 | (-9.845,-23.587,32.257) | 1 | (-9.753,-22.621,31.035) | 1.560 | 1 |
| 11 | 1a53 | (-12.173,-24.411,31.953) | 0 | (-11.898,-23.917,32.527) | 0.806 | 1 |
| 12 | 1a53 | (-9.908,-20.971,32.718) | 0 | (-9.853,-19.266,32.032) | 1.839 | 1 |
| 13 | 1a53 | (-12.160,-22.157,33.380) | 0 | (-11.24,-23.72,33.254) | 1.818 | 1 |
| 14 | 1a53 | (-7.551,-27.252,22.892) | 0 | (-5.127,-23.938,24.438) | 4.387 | 0 |
| 15 | 1a53 | (-11.204,-27.068,26.999) | 0 | (-11.357,-27.602,28.294) | 1.409 | 1 |
| 16 | 1a5b | (46.582,19.987,9.976) | 0 | (45.344,21.027,10.008) | 1.617 | 1 |
| 17 | 1a5b | (47.681,25.360,10.792) | 0 | (47.299,25.696,10.376) | 0.657 | 1 |
| 18 | 1a5b | (45.743,32.590,8.772) | 1 | (45.865,32.5,8.342) | 0.456 | 1 |
| 19 | 1a5b | (55.108,31.247,18.371) | 1 | (55.267,31.234,17.757) | 0.634 | 1 |
| 20 | 1a5b | (51.575,17.406,16.024) | 1 | (49.958,15.766,16.667) | 2.391 | 0 |
| 21 | 1a5b | (51.121,17.882,12.405) | 1 | (49.958,15.766,16.667) | 4.898 | 0 |
| 22 | 1a5b | (52.583,20.207,12.337) | 1 | (48.462,24.55,10.372) | 6.301 | 0 |
| 23 | 1a69 | (-18.200,70.294,77.594) | 1 | (-18.277,70.568,77.823) | 0.365 | 1 |
| 24 | 1a69 | (-22.451,73.116,63.687) | 0 | (-24.459,72.461,63.849) | 2.118 | 0 |
| 25 | 1a69 | (-18.786,74.868,63.550) | 1 | (-19.414,74.136,64.142) | 1.132 | 1 |
| 26 | 1a69 | (-16.972,76.720,63.256) | 1 | (-16.972,76.663,63.658) | 0.406 | 1 |
| 27 | 1a69 | (-15.006,77.784,61.689) | 1 | (-14.518,78.722,62.439) | 1.296 | 1 |
| 28 | 1a69 | (-21.427,68.942,70.366) | 1 | (-23.129,70.08,71.979) | 2.606 | 0 |
| 29 | 1a69 | (-19.577,78.681,62.221) | 1 | (-18.504,76.541,64.211) | 3.113 | 0 |
| 30 | 1a69 | (-26.399,68.732,61.678) | 0 | (-24.198,70.645,61.589) | 2.918 | 0 |
| 31 | 1a69 | (-21.790,76.296,63.365) | 1 | (-20.014,74.91,63.939) | 2.325 | 0 |
| 32 | 1a69 | (-25.326,71.451,65.873) | 0 | (-24.238,72.062,66.801) | 1.555 | 1 |
| 33 | 1a69 | (-23.946,69.260,73.619) | 1 | (-24.061,70.26,72.294) | 1.664 | 1 |
| 34 | 1as3 | (42.162,26.269,36.045) | 1 | (42.924,27.186,35.743) | 1.230 | 1 |
| 35 | 1as3 | (42.507,27.281,32.690) | 1 | (43.414,27.86,33.181) | 1.183 | 1 |
| 36 | 1as3 | (35.462,28.070,35.524) | 1 | (37.852,29.078,34.735) | 2.711 | 0 |
| 37 | 1as3 | (45.942,19.054,36.178) | 1 | (46.367,18.421,36.828) | 1.002 | 1 |
| 38 | 1as3 | (52.329,19.837,30.578) | 1 | (50.13,18.864,30.479) | 2.407 | 0 |
| 39 | 1as3 | (47.105,23.329,24.167) | 1 | (47.296,21.048,24.73) | 2.357 | 0 |
| 40 | 1as3 | (48.702,20.736,24.020) | 0 | (47.296,21.048,24.73) | 1.606 | 1 |
| 41 | 1as3 | (52.909,19.639,27.173) | 0 | (49.992,19.177,29.539) | 3.784 | 0 |
| 42 | 1as3 | (48.234,20.650,30.511) | 1 | (48.411,20.727,30.645) | 0.235 | 1 |
| 43 | 1as3 | (39.184,15.148,35.799) | 0 | (40.739,15.002,35.87) | 1.563 | 1 |
| 44 | 1as3 | (45.612,15.994,37.026) | 0 | (46.3,16.473,36.681) | 0.907 | 1 |
| 45 | 1as3 | (37.795,29.488,34.094) | 1 | (37.877,29.699,33.952) | 0.267 | 1 |
| 46 | 1as3 | (50.440,18.258,25.242) | 0 | (49.545,17.178,25.211) | 1.403 | 1 |
| 47 | 1btu | (-7.326,22.588,32.832) | 0 | (-5.956,22.435,35.097) | 2.652 | 0 |
| 48 | 1btu | (-3.960,22.318,30.606) | 1 | (-3.521,20.412,32.499) | 2.722 | 0 |
| 49 | 1btu | (-16.316,22.282,34.375) | 1 | (-14.85,18.914,39.4) | 6.224 | 0 |
| 50 | 1btu | (-5.932,24.619,35.765) | 0 | (-5.065,22.876,34.992) | 2.095 | 0 |
| 51 | 1btu | (-12.298,19.704,33.337) | 0 | (-6.926,19.448,34.821) | 5.579 | 0 |
| 52 | 1btu | (-15.329,19.795,37.101) | 0 | (-14.85,18.914,39.4) | 2.508 | 0 |
| 53 | 1btu | (-14.955,19.047,34.648) | 0 | (-14.85,18.914,39.4) | 4.755 | 0 |
| 54 | 1btu | (-11.590,14.064,40.627) | 0 | (-13.975,18.575,39.747) | 5.178 | 0 |
| 55 | 1btu | (-5.884,21.780,35.792) | 1 | (-5.906,21.753,35.827) | 0.049 | 1 |
| 56 | 1btu | (-5.446,23.844,32.088) | 0 | (-5.065,22.876,34.992) | 3.085 | 0 |
| 57 | 1dcp | (46.649,32.235,41.777) | 0 | (47.033,31.605,41.878) | 0.745 | 1 |
| 58 | 1dcp | (57.509,41.357,38.828) | 0 | (55.063,39.172,40.901) | 3.880 | 0 |
| 59 | 1dcp | (53.564,30.771,38.632) | 0 | (47.358,30.447,38.49) | 6.216 | 0 |
| 60 | 1dcp | (50.795,31.868,38.394) | 0 | (47.358,30.447,38.49) | 3.720 | 0 |
| 61 | 1di8 | (-15.564,46.652,9.370) | 0 | (-17.975,46.251,8.78) | 2.514 | 0 |
| 62 | 1di8 | (-12.461,47.248,3.696) | 1 | (-13.471,46.613,2.435) | 1.736 | 1 |
| 63 | 1di8 | (-15.182,50.011,5.158) | 1 | (-15.955,49.841,6.496) | 1.555 | 1 |
| 64 | 1di8 | (-16.832,52.349,4.966) | 0 | (-15.955,49.841,6.496) | 3.066 | 0 |
| 65 | 1di8 | (-16.795,48.497,7.181) | 1 | (-16.776,49.317,6.723) | 0.939 | 1 |
| 66 | 1di8 | (-10.064,46.450,6.465) | 0 | (-6.137,43.761,6.797) | 4.771 | 0 |
| 67 | 1di8 | (-3.109,47.316,19.864) | 0 | (-3.005,47.232,19.474) | 0.412 | 1 |
| 68 | 1di8 | (-15.260,47.269,3.834) | 0 | (-14.226,47.269,2.439) | 1.736 | 1 |
| 69 | 1e2p | (19.537,14.102,12.661) | 1 | (19.825,13.835,13.527) | 0.951 | 1 |
| 70 | 1e2p | (15.464,25.643,10.107) | 0 | (14.677,24.601,10.295) | 1.319 | 1 |
| 71 | 1e2p | (16.901,15.680,9.209) | 0 | (18.064,14.447,10.906) | 2.398 | 0 |
| 72 | 1e2p | (22.372,17.682,10.424) | 1 | (24.094,12.98,9.996) | 5.026 | 0 |
| 73 | 1e2p | (16.280,28.203,9.584) | 0 | (14.619,25.927,11.247) | 3.272 | 0 |
| 74 | 1e2p | (25.554,19.821,9.823) | 1 | (27.061,21.428,7.308) | 3.343 | 0 |
| 75 | 1eow | (7.347,7.010,19.129) | 1 | (4.681,1.833,20.791) | 6.056 | 0 |
| 76 | 1eow | (11.616,4.575,10.475) | 1 | (11.584,4.742,10.213) | 0.312 | 1 |
| 77 | 1eow | (19.256,-0.642,16.875) | 0 | (18.963,-0.686,16.746) | 0.323 | 1 |
| 78 | 1fcm | (74.977,4.237,18.714) | 1 | (74.574,2.262,18.801) | 2.018 | 0 |
| 79 | 1fcm | (69.426,4.703,24.184) | 1 | (68.882,4.748,24.235) | 0.548 | 1 |
| 80 | 1fcm | (80.960,2.848,24.392) | 0 | (83.524,3.404,22.747) | 3.097 | 0 |
| 81 | 1fcm | (73.396,2.437,20.051) | 1 | (73.507,2.25,20.283) | 0.318 | 1 |
| 82 | 1fcm | (73.847,7.725,36.514) | 0 | (73.952,7.758,36.695) | 0.212 | 1 |
| 83 | 1fcm | (80.069,-1.430,29.049) | 0 | (80.44,-1.989,28.532) | 0.847 | 1 |
| 84 | 1fcm | (75.586,13.623,25.907) | 1 | (75.24,15.686,27.756) | 2.792 | 0 |
| 85 | 1fcm | (73.195,4.676,22.499) | 1 | (73.018,4.82,22.06) | 0.495 | 1 |
| 86 | 1fcm | (74.064,6.021,26.612) | 0 | (74.12,5.525,26.352) | 0.563 | 1 |
| 87 | 1fcm | (83.177,6.844,19.561) | 1 | (82.934,6.489,19.182) | 0.573 | 1 |
| 88 | 1fcm | (73.067,6.532,19.514) | 1 | (73.661,5.924,20.182) | 1.081 | 1 |
| 89 | 1fcm | (76.670,8.171,38.539) | 0 | (77.144,8.501,39.065) | 0.781 | 1 |
| 90 | 1fcm | (78.043,-2.248,33.852) | 0 | (78.002,-2.537,34.22) | 0.470 | 1 |
| 91 | 1fcm | (84.170,4.496,37.617) | 0 | (82.127,4.946,38.154) | 2.160 | 0 |
| 92 | 1fcm | (78.247,6.970,21.905) | 0 | (77.133,4.973,20.588) | 2.639 | 0 |
| 93 | 1fcm | (75.590,6.041,22.416) | 0 | (74.246,5.583,22.819) | 1.476 | 1 |
| 94 | 1fcm | (76.178,9.467,36.199) | 1 | (76.917,9.454,36.197) | 0.739 | 1 |
| 95 | 1fup | (80.693,125.183,60.065) | 1 | (80.872,126.405,58.653) | 1.876 | 1 |
| 96 | 1fup | (71.786,123.423,64.171) | 1 | (70.961,122.132,66.341) | 2.656 | 0 |
| 97 | 1fup | (73.320,126.582,58.598) | 1 | (80.347,127.034,59.226) | 7.069 | 0 |
| 98 | 1fup | (82.600,123.410,56.631) | 0 | (82.197,124.177,56.747) | 0.874 | 1 |
| 99 | 1gbt | (43.537,-0.378,32.302) | 0 | (45.385,0.932,33.073) | 2.393 | 0 |
| 100 | 1gbt | (46.387,-6.105,18.255) | 1 | (45.419,-4.903,18.69) | 1.603 | 1 |
| 101 | 1gbt | (46.781,-2.613,19.551) | 1 | (47.588,-2.598,19.542) | 0.807 | 1 |
| 102 | 1gbt | (43.262,-8.861,23.383) | 1 | (43.821,-8.178,20.329) | 3.179 | 0 |
| 103 | 1gbt | (44.281,0.106,23.605) | 1 | (43.643,-0.519,23.285) | 0.949 | 1 |
| 104 | 1gbt | (41.282,0.183,29.356) | 0 | (39.32,-2.796,29.143) | 3.573 | 0 |
| 105 | 1gbt | (44.933,-8.502,19.726) | 1 | (44.769,-8.111,20.018) | 0.515 | 1 |
| 106 | 1gbt | (46.683,1.766,32.233) | 1 | (46.832,1.52,33.547) | 1.345 | 1 |
| 107 | 1gbt | (43.151,2.885,34.464) | 0 | (42.599,5.356,36.522) | 3.263 | 0 |
| 108 | 1gbt | (41.761,-5.479,28.503) | 0 | (39.847,-4.112,28.332) | 2.358 | 0 |
| 109 | 1h0c | (35.142,40.028,-6.419) | 0 | (35.052,41.251,-6.347) | 1.228 | 1 |
| 110 | 1h0c | (32.697,33.405,-19.450) | 1 | (32.616,33.393,-19.119) | 0.341 | 1 |
| 111 | 1h0c | (29.363,31.125,-7.586) | 1 | (30.347,31.11,-8.715) | 1.498 | 1 |
| 112 | 1h0s | (34.334,69.712,16.621) | 0 | (33.174,69.62,17.377) | 1.388 | 1 |
| 113 | 1h0s | (33.230,69.672,13.566) | 0 | (32.635,71.934,15.611) | 3.107 | 0 |
| 114 | 1h0s | (32.600,68.149,16.908) | 0 | (33.174,69.62,17.377) | 1.647 | 1 |
| 115 | 1h0s | (36.349,67.252,18.468) | 0 | (33.174,69.62,17.377) | 4.108 | 0 |
| 116 | 1h0s | (23.730,69.727,24.871) | 1 | (23.739,69.734,24.822) | 0.050 | 1 |
| 117 | 1h0s | (36.797,65.457,21.127) | 1 | (32.418,63.871,24.81) | 5.938 | 0 |
| 118 | 1h0s | (38.024,69.165,21.826) | 1 | (33.174,69.62,17.377) | 6.597 | 0 |
| 119 | 1h0s | (32.165,73.989,14.833) | 1 | (31.219,72.72,15.819) | 1.865 | 1 |
| 120 | 1h0s | (31.730,71.726,16.666) | 0 | (32.023,72.349,16.284) | 0.787 | 1 |
| 121 | 1h0s | (26.039,73.763,15.481) | 1 | (27.841,77.154,18.079) | 4.636 | 0 |
| 122 | 1i2l | (17.777,16.547,16.448) | 1 | (17.821,16.733,16.334) | 0.223 | 1 |
| 123 | 1i2l | (13.358,10.411,11.969) | 0 | (13.979,11.726,13.134) | 1.863 | 1 |
| 124 | 1i2l | (20.897,8.258,23.491) | 1 | (21.802,8.482,24.091) | 1.109 | 1 |
| 125 | 1i2l | (15.560,15.761,29.035) | 1 | (17.25,15.692,30.139) | 2.020 | 0 |
| 126 | 1i2l | (13.251,8.383,24.955) | 1 | (13.057,8.575,25.052) | 0.290 | 1 |
| 127 | 1i2l | (17.259,8.028,24.624) | 0 | (17.162,7.962,24.425) | 0.231 | 1 |
| 128 | 1i2l | (16.486,24.066,15.742) | 1 | (15.729,24.124,15.982) | 0.796 | 1 |
| 129 | 1i2l | (16.446,19.191,22.885) | 0 | (16.164,19.04,23.012) | 0.344 | 1 |
| 130 | 1i2l | (7.318,11.330,19.224) | 1 | (7.204,13.015,19.219) | 1.689 | 1 |
| 131 | 1if2 | (11.364,-0.986,18.695) | 1 | (11.408,-1.051,18.793) | 0.126 | 1 |
| 132 | 1if2 | (4.237,2.571,15.207) | 1 | (3.778,-0.58,16.089) | 3.304 | 0 |
| 133 | 1if2 | (10.631,-6.702,11.381) | 1 | (10.79,-6.578,11.265) | 0.233 | 1 |
| 134 | 1if2 | (3.350,-6.742,14.780) | 1 | (3.594,-7.088,14.943) | 0.454 | 1 |
| 135 | 1if2 | (4.267,-2.053,16.959) | 1 | (4.459,-0.797,16.788) | 1.282 | 1 |
| 136 | 1if2 | (8.541,1.016,20.752) | 1 | (8.127,0.989,20.824) | 0.421 | 1 |
| 137 | 1if2 | (3.043,0.522,16.598) | 1 | (3.778,-0.58,16.089) | 1.419 | 1 |
| 138 | 1if2 | (0.517,-0.005,15.780) | 1 | (3.778,-0.58,16.089) | 3.326 | 0 |
| 139 | 1if2 | (8.701,-1.074,25.337) | 0 | (7.24,-3.128,22.3) | 3.947 | 0 |
| 140 | 1if2 | (5.009,-1.255,21.027) | 1 | (6.418,-2.588,22.118) | 2.225 | 0 |
| 141 | 1if2 | (1.899,-3.113,18.270) | 0 | (2.816,-5.583,16.17) | 3.369 | 0 |
| 142 | 1if2 | (6.338,-2.791,22.698) | 0 | (5.85,-2.559,22.94) | 0.592 | 1 |
| 143 | 1if2 | (2.431,-5.919,17.249) | 0 | (2.816,-5.583,16.17) | 1.194 | 1 |
| 144 | 1jwb | (35.748,27.067,16.043) | 1 | (36.82,26.901,16.58) | 1.210 | 1 |
| 145 | 1jwb | (40.201,12.758,22.433) | 1 | (40.297,12.552,22.467) | 0.230 | 1 |
| 146 | 1jwb | (37.373,22.788,22.011) | 1 | (37.338,23.165,21.806) | 0.431 | 1 |
| 147 | 1jwb | (32.747,17.232,16.247) | 0 | (36.011,18.649,15.912) | 3.574 | 0 |
| 148 | 1jwb | (31.556,12.718,25.043) | 1 | (28.617,15.701,19.525) | 6.927 | 0 |
| 149 | 1jwb | (40.743,16.515,14.430) | 1 | (36.811,17.249,16.174) | 4.364 | 0 |
| 150 | 1jwb | (36.122,19.158,15.253) | 0 | (36.011,18.649,15.912) | 0.840 | 1 |
| 151 | 1jwb | (38.568,20.329,15.951) | 1 | (36.887,18.177,15.809) | 2.734 | 0 |
| 152 | 1jwb | (38.041,26.146,17.350) | 0 | (37.11,26.036,16.17) | 1.507 | 1 |
| 153 | 1jwb | (34.160,24.522,15.115) | 0 | (34.525,24.798,15.401) | 0.540 | 1 |
| 154 | 1jwb | (35.644,16.741,16.099) | 0 | (36.811,17.249,16.174) | 1.275 | 1 |
| 155 | 1jwb | (27.952,15.505,19.938) | 0 | (28.617,15.701,19.525) | 0.807 | 1 |
| 156 | 1jwb | (31.051,25.072,13.259) | 0 | (29.115,24.417,14.738) | 2.523 | 0 |
| 157 | 1k6r | (9.411,-13.279,81.398) | 1 | (9.567,-11.494,80.717) | 1.917 | 1 |
| 158 | 1k6r | (11.820,-19.561,87.155) | 1 | (11.405,-22.791,87.511) | 3.276 | 0 |
| 159 | 1k6r | (9.099,-17.915,85.233) | 0 | (11.405,-22.791,87.511) | 5.855 | 0 |
| 160 | 1k6r | (11.559,-24.894,87.403) | 0 | (11.665,-24.151,86.645) | 1.067 | 1 |
| 161 | 1k6r | (2.816,-16.581,84.031) | 0 | (2.469,-17.221,84.415) | 0.823 | 1 |
| 162 | 1k7e | (53.700,30.206,9.645) | 1 | (53.847,30.451,9.459) | 0.341 | 1 |
| 163 | 1k7e | (54.749,31.308,18.440) | 1 | (55.026,31.132,17.768) | 0.748 | 1 |
| 164 | 1k7e | (44.624,28.303,9.288) | 1 | (44.887,28.649,9.651) | 0.566 | 1 |
| 165 | 1k7e | (46.179,31.954,8.783) | 1 | (45.935,31.739,9.109) | 0.460 | 1 |
| 166 | 1k7e | (51.674,17.561,15.707) | 1 | (51.496,17.419,17.52) | 1.827 | 1 |
| 167 | 1k7e | (57.117,28.536,19.125) | 1 | (57.261,29.082,19.352) | 0.609 | 1 |
| 168 | 1k7e | (55.375,28.956,17.135) | 0 | (54.571,30.353,17.336) | 1.624 | 1 |
| 169 | 1k7e | (51.117,17.357,12.618) | 1 | (51.757,19.659,11.126) | 2.817 | 0 |
| 170 | 1k7e | (51.260,19.777,11.285) | 0 | (51.757,19.659,11.126) | 0.535 | 1 |
| 171 | 1k97 | (39.626,18.579,45.380) | 1 | (39.392,18.398,45.592) | 0.364 | 1 |
| 172 | 1k97 | (28.694,20.959,47.451) | 1 | (28.328,21.109,46.307) | 1.210 | 1 |
| 173 | 1k97 | (37.800,16.141,42.869) | 1 | (37.881,16.296,43.922) | 1.067 | 1 |
| 174 | 1k97 | (38.428,19.138,39.499) | 0 | (37.49,17.823,40.464) | 1.882 | 1 |
| 175 | 1k97 | (31.954,26.090,37.555) | 0 | (33.291,28.501,39.19) | 3.205 | 0 |
| 176 | 1k97 | (36.227,20.725,38.670) | 0 | (36.606,20.5,38.902) | 0.498 | 1 |
| 177 | 1k97 | (33.506,28.722,39.458) | 0 | (33.291,28.501,39.19) | 0.409 | 1 |
| 178 | 1k97 | (38.636,17.486,37.523) | 0 | (38.755,16.522,38.104) | 1.132 | 1 |
| 179 | 1k97 | (28.791,20.611,38.549) | 0 | (28.676,19.951,37.813) | 0.995 | 1 |
| 180 | 1k97 | (29.391,26.215,38.451) | 0 | (28.897,26.047,41.444) | 3.038 | 0 |
| 181 | 1k97 | (36.685,20.804,35.685) | 0 | (34.538,20.879,37.837) | 3.041 | 0 |
| 182 | 1lbf | (-4.098,-20.645,26.236) | 1 | (-4.037,-20.675,26.107) | 0.146 | 1 |
| 183 | 1lbf | (-5.018,-24.217,24.922) | 1 | (-4.789,-24.296,24.947) | 0.244 | 1 |
| 184 | 1lbf | (-12.230,-14.296,34.044) | 1 | (-11.354,-14.481,34.262) | 0.921 | 1 |
| 185 | 1lbf | (-4.949,-15.895,25.368) | 0 | (-4.804,-15.904,25.573) | 0.251 | 1 |
| 186 | 1lbf | (-18.412,-24.479,35.979) | 0 | (-19.749,-18.676,38.279) | 6.384 | 0 |
| 187 | 1lbf | (-7.480,-24.642,23.385) | 0 | (-5.162,-23.811,24.156) | 2.58 | 0 |
| 188 | 1lbf | (-9.150,-23.857,25.587) | 0 | (-8.982,-23.949,26.489) | 0.922 | 1 |
| 189 | 1lbf | (-9.558,-21.922,29.141) | 0 | (-9.252,-21.282,28.89) | 0.752 | 1 |
| 190 | 1lbf | (-13.482,-23.601,25.105) | 0 | (-10.401,-24.755,26.422) | 3.544 | 0 |
| 191 | 1lbf | (-7.856,-16.483,23.944) | 0 | (-5.767,-15.484,22.838) | 2.566 | 0 |
| 192 | 1lbf | (-7.186,-18.582,35.836) | 0 | (-7.63,-18.764,36.505) | 0.823 | 1 |
| 193 | 1lbf | (-7.187,-20.211,38.558) | 0 | (-6.813,-21.045,39.145) | 1.086 | 1 |
| 194 | 1lbf | (-9.996,-26.126,29.622) | 0 | (-9.964,-25.868,29.441) | 0.317 | 1 |
| 195 | 1lbf | (-11.124,-26.253,26.903) | 0 | (-10.401,-24.755,26.422) | 1.732 | 1 |
| 196 | 1lbf | (-9.860,-19.250,32.292) | 0 | (-9.628,-19.29,31.758) | 0.584 | 1 |
| 197 | 1lbf | (-7.456,-27.597,22.581) | 0 | (-5.162,-23.811,24.156) | 4.699 | 0 |
| 198 | 1lbl | (-4.191,-20.819,26.334) | 1 | (-3.968,-20.771,26.222) | 0.254 | 1 |
| 199 | 1lbl | (-5.218,-24.375,25.029) | 1 | (-4.646,-23.9,24.617) | 0.850 | 1 |
| 200 | 1lbl | (-5.053,-16.034,25.356) | 0 | (-5.879,-15.799,24.949) | 0.950 | 1 |
| 201 | 1lbl | (-17.833,-25.464,36.455) | 0 | (-15.837,-29.604,36.646) | 4.600 | 0 |
| 202 | 1lbl | (-7.548,-24.931,23.242) | 0 | (-7.283,-23.613,22.595) | 1.492 | 1 |
| 203 | 1lbl | (-13.006,-23.494,25.826) | 0 | (-12.608,-27.749,27.546) | 4.607 | 0 |
| 204 | 1lbl | (-10.515,-26.189,29.141) | 0 | (-10.41,-26.418,29.125) | 0.252 | 1 |
| 205 | 1lbl | (-9.066,-23.647,25.627) | 0 | (-7.283,-23.613,22.595) | 3.518 | 0 |
| 206 | 1lbl | (-11.360,-28.058,26.251) | 0 | (-12.608,-27.749,27.546) | 1.825 | 1 |
| 207 | 1lbl | (-13.764,-21.999,28.539) | 0 | (-11.378,-26.354,29.368) | 5.035 | 0 |
| 208 | 1lbl | (-8.030,-16.701,24.074) | 0 | (-5.879,-15.799,24.949) | 2.491 | 0 |
| 209 | 1mqh | (18.758,48.245,14.389) | 1 | (18.656,48.17,14.364) | 0.129 | 1 |
| 210 | 1mqh | (20.481,39.959,11.805) | 0 | (20.76,37.632,11.05) | 2.462 | 0 |
| 211 | 1mqh | (13.010,47.781,15.083) | 1 | (10.824,44.307,17.413) | 4.720 | 0 |
| 212 | 1mqh | (13.541,46.564,11.126) | 1 | (13.469,45.963,10.621) | 0.788 | 1 |
| 213 | 1mqh | (10.830,43.830,20.095) | 1 | (11.003,44.143,20.191) | 0.370 | 1 |
| 214 | 1mqh | (17.400,50.364,22.834) | 1 | (17.086,50.844,22.665) | 0.598 | 1 |
| 215 | 1mqh | (13.548,42.481,10.495) | 1 | (13.549,42.448,10.557) | 0.070 | 1 |
| 216 | 1mqh | (9.631,40.590,16.143) | 1 | (9.763,40.587,15.788) | 0.379 | 1 |
| 217 | 1mqh | (10.970,39.325,14.303) | 1 | (11.296,39.657,13.929) | 0.597 | 1 |
| 218 | 1mqh | (13.350,35.669,11.378) | 1 | (13.926,35.651,10.9) | 0.749 | 1 |
| 219 | 1mqh | (10.560,45.731,22.951) | 0 | (11.306,45.902,22.926) | 0.766 | 1 |
| 220 | 1mqh | (15.107,49.695,24.188) | 1 | (15.081,49.586,21.783) | 2.408 | 0 |
| 221 | 1mqh | (12.558,46.539,21.472) | 1 | (12.902,46.251,21.537) | 0.453 | 1 |
| 222 | 1mqh | (10.356,43.778,17.609) | 1 | (10.361,43.547,17.87) | 0.349 | 1 |
| 223 | 1mqh | (13.546,49.300,21.389) | 1 | (15.081,49.586,21.783) | 1.610 | 1 |
| 224 | 1nc3 | (49.185,51.513,45.411) | 0 | (50.746,51.479,46.663) | 2.001 | 0 |
| 225 | 1nc3 | (34.740,53.325,43.781) | 1 | (34.584,53.361,44.411) | 0.650 | 1 |
| 226 | 1nc3 | (53.884,48.982,45.204) | 0 | (54.207,49.356,44.228) | 1.094 | 1 |
| 227 | 1nc3 | (51.761,50.226,46.754) | 1 | (51.228,50.603,46.64) | 0.663 | 1 |
| 228 | 1nc3 | (45.057,47.180,37.291) | 1 | (50.936,43.774,38.612) | 6.922 | 0 |
| 229 | 1ne4 | (37.700,44.751,81.291) | 1 | (37.818,44.918,81.304) | 0.205 | 1 |
| 230 | 1ne4 | (46.419,41.463,89.286) | 1 | (46.875,41.875,89.521) | 0.658 | 1 |
| 231 | 1ne4 | (47.307,52.811,83.321) | 1 | (46.865,51.917,84.194) | 1.325 | 1 |
| 232 | 1ne4 | (45.985,51.210,85.229) | 1 | (46.528,51.382,84.969) | 0.626 | 1 |
| 233 | 1ne4 | (48.629,50.889,88.697) | 1 | (48.47,50.974,88.064) | 0.658 | 1 |
| 234 | 1ne4 | (48.634,36.087,84.777) | 1 | (46.154,39.508,85.261) | 4.253 | 0 |
| 235 | 1np0 | (60.769,42.075,25.353) | 1 | (60.934,42.363,25.145) | 0.392 | 1 |
| 236 | 1np0 | (58.298,51.509,19.388) | 1 | (57.205,53.445,20.678) | 2.57 | 0 |
| 237 | 1np0 | (60.670,47.754,15.713) | 1 | (60.38,47.691,15.047) | 0.729 | 1 |
| 238 | 1np0 | (66.613,43.235,20.937) | 1 | (61.253,45.316,18.712) | 6.165 | 0 |
| 239 | 1nrr | (8.984,-15.778,27.517) | 0 | (8.919,-16.247,27.139) | 0.606 | 1 |
| 240 | 1nrr | (12.442,-8.058,25.047) | 0 | (8.562,-8.813,27.267) | 4.534 | 0 |
| 241 | 1nrr | (12.842,-14.977,23.012) | 0 | (9.013,-16.621,26.216) | 5.256 | 0 |
| 242 | 1nrr | (8.432,-8.134,27.706) | 1 | (8.646,-7.968,27.794) | 0.285 | 1 |
| 243 | 1nrr | (25.713,-15.653,23.214) | 1 | (25.864,-13.655,23.788) | 2.084 | 0 |
| 244 | 1nrr | (15.876,-15.893,20.416) | 0 | (15.831,-10.201,15.556) | 7.485 | 0 |
| 245 | 1nrr | (13.126,-12.621,12.679) | 0 | (15.026,-11.589,11.255) | 2.589 | 0 |
| 246 | 1nrr | (15.385,-12.303,15.693) | 1 | (15.15,-10.694,15.014) | 1.762 | 1 |
| 247 | 1nrr | (6.776,-10.472,29.122) | 1 | (6.592,-10.472,29.451) | 0.377 | 1 |
| 248 | 1nrr | (13.861,-18.179,23.183) | 0 | (9.013,-16.621,26.216) | 5.927 | 0 |
| 249 | 1nrr | (11.977,-14.126,28.553) | 0 | (12.11,-13.705,29.126) | 0.723 | 1 |
| 250 | 1nrr | (25.099,-15.396,31.336) | 1 | (24.786,-15.26,31.132) | 0.398 | 1 |
| 251 | 1nrr | (16.071,-19.191,24.970) | 1 | (18.218,-18.649,30.823) | 6.258 | 0 |
| 252 | 1nrr | (9.755,-13.598,27.893) | 1 | (8.061,-12.968,27.947) | 1.808 | 1 |
| 253 | 1nrr | (20.680,-12.479,16.285) | 0 | (18.146,-10.349,13.345) | 4.427 | 0 |
| 254 | 1nrr | (10.626,-18.121,21.303) | 0 | (9.013,-16.621,26.216) | 5.384 | 0 |
| 255 | 1nrr | (24.813,-17.328,28.251) | 0 | (24.767,-16.13,30.64) | 2.673 | 0 |
| 256 | 1oeo | (51.826,20.624,5.102) | 0 | (51.797,20.639,5.181) | 0.085 | 1 |
| 257 | 1oeo | (44.031,18.867,5.042) | 0 | (43.974,20.577,3.997) | 2.005 | 0 |
| 258 | 1oeo | (44.591,16.209,4.607) | 0 | (46.198,14.752,6.491) | 2.873 | 0 |
| 259 | 1oeo | (49.032,20.183,4.434) | 0 | (48.614,18.227,5.195) | 2.140 | 0 |
| 260 | 1oeo | (46.344,14.805,6.416) | 0 | (46.198,14.752,6.491) | 0.172 | 1 |
| 261 | 1oyf | (9.883,32.243,35.332) | 0 | (9.755,32.249,35.3) | 0.132 | 1 |
| 262 | 1oyf | (21.422,26.946,26.804) | 0 | (23.954,27.495,27.898) | 2.812 | 0 |
| 263 | 1qcp | (44.220,24.757,56.052) | 0 | (42.86,23.166,55.508) | 2.163 | 0 |
| 264 | 1qcp | (46.274,21.714,53.747) | 1 | (42.86,23.166,55.508) | 4.107 | 0 |
| 265 | 1qcp | (38.292,24.767,55.312) | 0 | (36.933,24.929,54.702) | 1.498 | 1 |
| 266 | 1qcp | (45.629,11.890,54.006) | 1 | (46.763,11.145,53.928) | 1.359 | 1 |
| 267 | 1qcp | (50.155,24.350,44.488) | 0 | (50.867,20.644,42.131) | 4.449 | 0 |
| 268 | 1qcp | (35.507,28.005,46.045) | 0 | (36.753,24.554,53.793) | 8.573 | 0 |
| 269 | 1qcp | (45.526,27.231,52.317) | 0 | (42.105,23.725,55.165) | 5.666 | 0 |
| 270 | 1qcp | (49.021,25.990,46.565) | 0 | (51.23,23.132,50.834) | 5.592 | 0 |
| 271 | 1qcp | (47.415,16.509,41.569) | 1 | (47.213,16.599,41.622) | 0.227 | 1 |
| 272 | 1qcp | (48.142,14.317,58.195) | 1 | (48.587,14.906,58.215) | 0.738 | 1 |
| 273 | 1qcp | (45.487,25.054,53.766) | 0 | (42.86,23.166,55.508) | 3.674 | 0 |
| 274 | 1qcp | (45.544,14.787,42.714) | 1 | (45.722,15.085,42.548) | 0.385 | 1 |
| 275 | 1qcp | (48.436,18.048,58.162) | 1 | (47.883,15.339,58.778) | 2.833 | 0 |
| 276 | 1qcp | (45.641,26.740,49.802) | 0 | (51.23,23.132,50.834) | 6.732 | 0 |
| 277 | 1qcp | (48.459,24.358,42.523) | 0 | (50.867,20.644,42.131) | 4.444 | 0 |
| 278 | 1qcp | (42.506,16.075,52.002) | 1 | (45.848,11.897,51.847) | 5.352 | 0 |
| 279 | 1qcp | (47.350,11.922,57.028) | 1 | (46.722,11.618,56.494) | 0.879 | 1 |
| 280 | 1qcp | (47.218,24.554,55.433) | 0 | (42.86,23.166,55.508) | 4.574 | 0 |
| 281 | 1qcp | (38.677,26.711,53.693) | 0 | (36.481,25.816,54.792) | 2.614 | 0 |
| 282 | 1qcp | (43.932,28.291,40.616) | 0 | (50.06,20.29,41.658) | 10.132 | 0 |
| 283 | 1qcp | (49.167,27.594,43.770) | 0 | (50.867,20.644,42.131) | 7.340 | 0 |
| 284 | 1qcp | (41.484,28.358,41.543) | 0 | (50.06,20.29,41.658) | 11.775 | 0 |
| 285 | 1qcp | (49.489,23.624,54.403) | 0 | (51.23,23.132,50.834) | 4.001 | 0 |
| 286 | 1qcp | (43.483,23.008,52.594) | 0 | (42.86,23.166,55.508) | 2.984 | 0 |
| 287 | 1qj6 | (-2.835,16.376,41.929) | 1 | / | / | / |
| 288 | 1qj6 | (7.381,22.987,57.155) | 0 | / | / | / |
| 289 | 1qj6 | (3.091,24.404,59.276) | 1 | / | / | / |
| 290 | 1qj6 | (1.447,16.092,49.279) | 1 | / | / | / |
| 291 | 1qj6 | (-3.258,18.802,48.186) | 1 | / | / | / |
| 292 | 1qj6 | (-3.109,18.395,39.967) | 1 | / | / | / |
| 293 | 1qj6 | (15.648,23.233,42.787) | 1 | / | / | / |
| 294 | 1qj6 | (14.566,20.463,43.417) | 1 | / | / | / |
| 295 | 1qj6 | (13.546,25.616,44.080) | 1 | / | / | / |
| 296 | 1qj6 | (4.576,14.222,39.759) | 1 | / | / | / |
| 297 | 1qj6 | (13.052,18.161,44.306) | 1 | / | / | / |
| 298 | 1qj6 | (9.564,25.708,46.431) | 1 | / | / | / |
| 299 | 1qj6 | (3.918,23.395,56.275) | 0 | / | / | / |
| 300 | 1qj6 | (11.613,20.488,44.588) | 1 | / | / | / |
| 301 | 1spa | (17.839,2.710,-15.340) | 1 | (18.371,3.03,-14.675) | 0.910 | 1 |
| 302 | 1spa | (23.421,2.694,-20.124) | 1 | (24.159,2.711,-20.257) | 0.750 | 1 |
| 303 | 1spa | (24.201,11.892,-13.702) | 0 | (24.544,11.103,-14.353) | 1.079 | 1 |
| 304 | 1spa | (25.992,5.727,-19.293) | 1 | (25.733,5.682,-20.162) | 0.908 | 1 |
| 305 | 1spa | (21.833,10.076,-14.657) | 0 | (24.544,11.103,-14.353) | 2.915 | 0 |
| 306 | 1spa | (23.542,8.099,-8.342) | 0 | (24.248,7.515,-8.162) | 0.934 | 1 |
| 307 | 1spa | (28.587,13.773,-16.529) | 1 | (28.117,13.644,-16.503) | 0.488 | 1 |
| 308 | 1spa | (23.117,11.195,-8.080) | 0 | (25.231,12.531,-10.406) | 3.415 | 0 |
| 309 | 1spa | (20.581,5.018,-13.431) | 0 | (20.989,4.901,-13.278) | 0.451 | 1 |
| 310 | 1syi | (15.295,12.357,85.877) | 1 | (15.119,12.297,85.832) | 0.191 | 1 |
| 311 | 1syi | (14.361,12.720,91.928) | 0 | (16.054,9.128,91.801) | 3.973 | 0 |
| 312 | 1syi | (13.195,21.389,89.403) | 1 | (14.312,20.442,90.102) | 1.623 | 1 |
| 313 | 1syi | (13.380,12.361,83.969) | 0 | (14.389,10.756,82.772) | 2.242 | 0 |
| 314 | 1syi | (16.353,19.067,92.984) | 0 | (17.02,18.602,93.528) | 0.978 | 1 |
| 315 | 1syi | (12.752,20.317,87.054) | 1 | (12.515,20.643,87.307) | 0.476 | 1 |
| 316 | 1syi | (23.651,9.682,86.708) | 0 | (22.837,9.349,86.308) | 0.966 | 1 |
| 317 | 1syi | (26.827,9.024,93.905) | 1 | (26.551,8.828,93.049) | 0.921 | 1 |
| 318 | 1syi | (15.728,9.299,91.716) | 1 | (16.054,9.128,91.801) | 0.378 | 1 |
| 319 | 1thn | (-2.539,28.328,48.977) | 0 | (-2.415,28.266,49.231) | 0.289 | 1 |
| 320 | 1thn | (-5.304,34.523,44.910) | 0 | (-3.811,32.445,44.746) | 2.564 | 0 |
| 321 | 1thn | (6.625,31.865,38.760) | 1 | (7.853,30.502,38.601) | 1.841 | 1 |
| 322 | 1thn | (9.613,28.002,39.488) | 0 | (10.079,27.825,39.39) | 0.508 | 1 |
| 323 | 1thn | (4.456,30.307,47.810) | 1 | (3.016,28.262,51.906) | 4.799 | 0 |
| 324 | 1tnj | (32.142,13.950,10.624) | 1 | (31.903,13.838,10.821) | 0.329 | 1 |
| 325 | 1tnj | (29.517,8.817,15.034) | 0 | (26.458,9.407,12.931) | 3.759 | 0 |
| 326 | 1tnj | (29.454,21.047,13.196) | 1 | (29.647,20.527,11.416) | 1.864 | 1 |
| 327 | 1tnj | (26.550,17.402,16.745) | 1 | (28.741,14.731,21.589) | 5.950 | 0 |
| 328 | 1tnj | (29.440,13.390,24.554) | 0 | (29.59,11.868,22.796) | 2.330 | 0 |
| 329 | 1tnj | (32.227,17.549,9.511) | 1 | (32.448,17.092,9.593) | 0.514 | 1 |
| 330 | 1tnj | (31.243,20.277,10.118) | 1 | (30.442,20.437,10.816) | 1.074 | 1 |
| 331 | 1tnj | (25.011,11.090,19.500) | 1 | (23.75,11.222,20.109) | 1.407 | 1 |
| 332 | 1tph | (-6.011,21.730,30.552) | 1 | (-6.483,20.893,32.812) | 2.456 | 0 |
| 333 | 1tph | (-14.217,22.102,26.289) | 1 | (-14.362,22.052,26.374) | 0.175 | 1 |
| 334 | 1tph | (-5.200,16.532,24.431) | 1 | (-6.361,15.357,25.252) | 1.845 | 1 |
| 335 | 1tph | (-15.035,21.218,22.431) | 1 | (-14.362,22.052,26.374) | 4.086 | 0 |
| 336 | 1tph | (-9.077,19.132,21.734) | 1 | (-9.44,19.465,20.61) | 1.227 | 1 |
| 337 | 1tph | (-8.880,23.354,19.333) | 1 | (-9.166,21.068,20.461) | 2.565 | 0 |
| 338 | 1tph | (-4.799,16.250,27.449) | 1 | (-6.075,15.848,26.075) | 1.918 | 1 |
| 339 | 1tph | (-12.953,17.957,21.348) | 1 | (-9.44,19.465,20.61) | 3.894 | 0 |
| 340 | 1tph | (-15.675,18.843,20.677) | 1 | (-19.168,15.998,24.187) | 5.711 | 0 |
| 341 | 1tph | (-8.526,16.637,20.579) | 1 | (-9.44,19.465,20.61) | 2.972 | 0 |
| 342 | 1tph | (-8.751,20.568,19.254) | 1 | (-9.166,21.068,20.461) | 1.371 | 1 |
| 343 | 1ype | (7.070,8.826,-0.321) | 0 | (6.65,6.651,-0.431) | 2.218 | 0 |
| 344 | 1ype | (12.861,12.664,2.974) | 0 | (15.653,9.384,2.34) | 4.354 | 0 |
| 345 | 1ype | (5.623,7.643,-6.863) | 0 | (6.994,6.052,-1.154) | 6.083 | 0 |
| 346 | 1ype | (6.166,5.340,-6.528) | 0 | (6.994,6.052,-1.154) | 5.484 | 0 |
| 347 | 1zui | (39.607,6.654,-0.510) | 1 | (39.659,6.349,0.24) | 0.811 | 1 |
| 348 | 1zui | (43.653,10.508,2.828) | 1 | (43.497,10.34,3.225) | 0.458 | 1 |
| 349 | 1zui | (33.883,8.304,3.110) | 1 | (34.3,8,2.899) | 0.558 | 1 |
| 350 | 1zui | (47.140,9.054,-4.779) | 1 | (43.453,6.229,0.552) | 7.071 | 0 |
| 351 | 1zui | (38.701,10.608,4.969) | 0 | (37.538,9.819,4.137) | 1.633 | 1 |
| 352 | 1zui | (35.742,5.838,0.096) | 1 | (35.291,5.267,-0.182) | 0.779 | 1 |
| 353 | 1zui | (36.222,8.026,6.213) | 0 | (35.44,7.17,6.756) | 1.280 | 1 |
| 354 | 1zui | (46.848,5.000,-2.507) | 0 | (43.453,6.229,0.552) | 4.732 | 0 |
| 355 | 1zui | (34.910,10.463,4.906) | 0 | (33.971,10.404,4.052) | 1.271 | 1 |
| 356 | 2dhn | (45.855,49.095,73.903) | 1 | (44.909,50.4,73.496) | 1.662 | 1 |
| 357 | 2dhn | (50.073,45.198,70.518) | 0 | (47.502,45.353,73.872) | 4.229 | 0 |
| 358 | 2hmv | (120.195,43.011,33.202) | 1 | (117.189,45.787,36.297) | 5.130 | 0 |
| 359 | 2hmv | (112.916,40.904,33.657) | 1 | (112.908,40.932,33.244) | 0.414 | 1 |
| 360 | 2hmv | (123.445,43.378,35.179) | 1 | (126.659,39.445,37.887) | 5.756 | 0 |
| 361 | 2hmv | (122.460,46.120,39.929) | 0 | (117.646,49.453,40.958) | 5.945 | 0 |
| 362 | 2hmv | (125.383,44.120,40.835) | 1 | (126.659,39.445,37.887) | 5.672 | 0 |
| 363 | 2hmv | (114.173,44.860,29.007) | 0 | (112.686,43.984,31.33) | 2.894 | 0 |
| 364 | 2hmv | (118.410,46.320,38.931) | 0 | (117.189,45.787,36.297) | 2.952 | 0 |
| 365 | 2hmv | (111.304,47.222,29.039) | 0 | (111.518,43.38,30.363) | 4.069 | 0 |
| 366 | 2hmv | (122.577,45.568,42.518) | 1 | (117.646,49.453,40.958) | 6.469 | 0 |
| 367 | 2hmv | (112.237,43.189,31.376) | 1 | (112.131,43.178,31.127) | 0.271 | 1 |
| 368 | 2hmv | (117.028,46.414,29.479) | 0 | (112.686,43.984,31.33) | 5.309 | 0 |
| 369 | 2hmv | (120.874,45.235,34.967) | 0 | (117.569,47.244,35.663) | 3.930 | 0 |
| 370 | 2ica | (-11.212,1.791,32.930) | 0 | (-10.625,-0.955,34.418) | 3.178 | 0 |
| 371 | 2ica | (-12.388,6.298,28.891) | 0 | (-10.625,-0.955,34.418) | 9.288 | 0 |
| 372 | 2ica | (-12.787,1.956,35.711) | 0 | (-10.966,-1.354,35.269) | 3.804 | 0 |
| 373 | 2ica | (-14.443,3.662,25.989) | 0 | (-13.321,6.194,18.506) | 7.979 | 0 |
| 374 | 2ica | (-14.866,3.102,31.935) | 0 | (-10.625,-0.955,34.418) | 6.373 | 0 |
| 375 | 2ica | (-8.116,11.600,22.797) | 0 | (-7.436,11.5,15.916) | 6.915 | 0 |
| 376 | 2ica | (-13.745,0.941,21.976) | 0 | (-12.774,0.297,17.502) | 4.623 | 0 |
| 377 | 2is0 | (21.549,39.443,-4.225) | 1 | / | / | / |
| 378 | 2is0 | (23.459,37.753,-1.656) | 0 | / | / | / |
| 379 | 2is0 | (38.601,48.746,6.887) | 1 | / | / | / |
| 380 | 2is0 | (34.600,43.656,-2.158) | 0 | / | / | / |
| 381 | 2is0 | (34.487,48.779,9.103) | 0 | / | / | / |
| 382 | 2is0 | (34.080,29.547,2.631) | 1 | / | / | / |
| 383 | 2is0 | (21.865,44.295,-0.705) | 1 | / | / | / |
| 384 | 2is0 | (26.205,39.727,1.998) | 1 | / | / | / |
| 385 | 2is0 | (34.343,42.814,9.289) | 1 | / | / | / |
| 386 | 2is0 | (21.395,50.518,0.586) | 0 | / | / | / |
| 387 | 2is0 | (28.619,35.507,-0.243) | 0 | / | / | / |
| 388 | 2is0 | (23.629,35.415,1.631) | 1 | / | / | / |
| 389 | 2is0 | (26.325,36.945,1.271) | 0 | / | / | / |
| 390 | 2is0 | (26.377,50.092,2.479) | 0 | / | / | / |
| 391 | 2is0 | (29.606,52.622,11.476) | 0 | / | / | / |
| 392 | 2is0 | (29.513,48.456,1.833) | 0 | / | / | / |
| 393 | 2is0 | (23.600,48.752,-0.558) | 1 | / | / | / |
| 394 | 2is0 | (29.661,47.464,-0.922) | 0 | / | / | / |
| 395 | 2is0 | (32.707,48.389,0.928) | 0 | / | / | / |
| 396 | 2is0 | (27.123,42.284,-5.005) | 0 | / | / | / |
| 397 | 2is0 | (34.205,46.760,-3.093) | 0 | / | / | / |
| 398 | 2is0 | (26.584,50.659,10.623) | 0 | / | / | / |
| 399 | 2is0 | (28.243,50.175,-1.708) | 0 | / | / | / |
| 400 | 2is0 | (36.363,32.937,3.334) | 1 | / | / | / |
| 401 | 2jfh | (13.881,-21.160,9.083) | 0 | (11.633,-20.171,10.306) | 2.744 | 0 |
| 402 | 2jfh | (26.911,-7.647,15.668) | 1 | (26.842,-7.348,15.593) | 0.316 | 1 |
| 403 | 2jfh | (20.768,-9.118,16.510) | 1 | (20.905,-9.407,16.402) | 0.338 | 1 |
| 404 | 2jfh | (26.456,-11.017,16.614) | 1 | (26.676,-11.346,16.357) | 0.472 | 1 |
| 405 | 2jfh | (13.860,-17.407,17.127) | 1 | (14.037,-15.5,17.926) | 2.075 | 0 |
| 406 | 2jfh | (14.280,-20.266,11.615) | 1 | (11.633,-20.171,10.306) | 2.955 | 0 |
| 407 | 2jfh | (12.955,-16.730,14.836) | 0 | (14.037,-15.5,17.926) | 3.497 | 0 |
| 408 | 2jfh | (23.605,-8.845,15.719) | 1 | (23.671,-7.918,15.306) | 1.017 | 1 |
| 409 | 2jfh | (14.269,-13.682,6.346) | 0 | (13.77,-13.607,7.839) | 1.576 | 1 |
| 410 | 2jfh | (26.814,-7.910,18.952) | 1 | (26.728,-7.668,19.216) | 0.368 | 1 |
| 411 | 2jfh | (29.597,-8.824,13.260) | 1 | (29.811,-9.153,14.15) | 0.973 | 1 |
| 412 | 2p4t | (47.932,29.551,30.285) | 1 | (48.174,29.469,30.296) | 0.256 | 1 |
| 413 | 2p4t | (48.139,27.116,26.656) | 1 | (46.627,27.266,27.641) | 1.811 | 1 |
| 414 | 2p4t | (44.505,24.871,33.102) | 1 | (48.955,26.077,32.089) | 4.720 | 0 |
| 415 | 2p4t | (45.630,28.751,26.772) | 0 | (46.755,28.256,27.7) | 1.540 | 1 |
| 416 | 2p4t | (34.863,29.788,29.814) | 0 | (36.45,29.173,30.962) | 2.053 | 0 |
| 417 | 2p4t | (34.748,29.922,27.247) | 0 | (36.45,29.173,30.962) | 4.154 | 0 |
| 418 | 2p4t | (37.774,29.059,30.814) | 1 | (37.275,28.973,31.491) | 0.845 | 1 |
| 419 | 2p4t | (33.230,41.225,30.726) | 1 | (43.767,41.85,29.817) | 10.595 | 0 |
| 420 | 2p4t | (35.499,33.817,30.303) | 1 | (36.45,29.173,30.962) | 4.786 | 0 |
| 421 | 2p4t | (36.913,26.012,33.788) | 1 | (37.275,28.973,31.491) | 3.765 | 0 |
| 422 | 2p4t | (45.035,29.632,24.338) | 1 | (44.863,29.464,25.039) | 0.741 | 1 |
| 423 | 2p4t | (36.886,26.469,30.999) | 0 | (37.275,28.973,31.491) | 2.581 | 0 |
| 424 | 2p4t | (42.440,35.481,26.296) | 0 | (43.735,40.754,28.607) | 5.901 | 0 |
| 425 | 2p4t | (32.913,38.918,31.555) | 0 | (37.765,29.824,31.679) | 10.308 | 0 |
| 426 | 2p4t | (39.148,26.758,30.713) | 1 | (37.275,28.973,31.491) | 3.003 | 0 |
| 427 | 2p4t | (44.109,26.225,30.783) | 1 | (46.627,27.266,27.641) | 4.159 | 0 |
| 428 | 2ph6 | (33.977,42.538,9.063) | 1 | / | / | / |
| 429 | 2ph6 | (21.183,39.045,-4.189) | 0 | / | / | / |
| 430 | 2ph6 | (33.956,48.485,8.868) | 1 | / | / | / |
| 431 | 2ph6 | (28.959,34.306,-0.134) | 0 | / | / | / |
| 432 | 2ph6 | (25.457,39.257,1.724) | 1 | / | / | / |
| 433 | 2ph6 | (34.227,43.572,-2.282) | 1 | / | / | / |
| 434 | 2ph6 | (36.201,32.957,3.136) | 1 | / | / | / |
| 435 | 2ph6 | (25.880,50.300,10.729) | 0 | / | / | / |
| 436 | 2ph6 | (25.326,36.613,1.247) | 0 | / | / | / |
| 437 | 2ph6 | (28.783,48.300,1.564) | 0 | / | / | / |
| 438 | 2ph6 | (32.069,48.096,1.055) | 0 | / | / | / |
| 439 | 2ph6 | (31.588,49.979,9.827) | 0 | / | / | / |
| 440 | 2ph6 | (26.472,41.764,-4.399) | 0 | / | / | / |
| 441 | 2ph6 | (22.841,48.799,-0.512) | 1 | / | / | / |
| 442 | 2ph6 | (30.834,50.218,3.536) | 0 | / | / | / |
| 443 | 2ph6 | (20.624,50.156,0.448) | 0 | / | / | / |
| 444 | 2ph6 | (29.032,52.528,11.456) | 0 | / | / | / |
| 445 | 2ph6 | (25.104,33.587,1.399) | 1 | / | / | / |
| 446 | 2ph6 | (38.175,48.702,6.746) | 1 | / | / | / |
| 447 | 2ph6 | (33.916,46.036,-3.499) | 1 | / | / | / |
| 448 | 2ph6 | (24.506,35.355,-3.335) | 0 | / | / | / |
| 449 | 2ph6 | (19.828,47.110,-3.182) | 0 | / | / | / |
| 450 | 2ph6 | (26.803,36.164,3.147) | 0 | / | / | / |
| 451 | 2ph6 | (28.698,46.782,-1.395) | 0 | / | / | / |
| 452 | 2ph6 | (21.544,44.067,-0.904) | 1 | / | / | / |
| 453 | 2ph6 | (34.714,50.797,12.812) | 0 | / | / | / |
| 454 | 2ph6 | (22.457,37.723,-2.303) | 0 | / | / | / |
| 455 | 2q9c | (52.245,58.068,55.019) | 1 | (52.203,58.066,55.207) | 0.193 | 1 |
| 456 | 2q9c | (55.608,61.565,48.902) | 1 | (54.383,61.263,48.608) | 1.295 | 1 |
| 457 | 2q9c | (53.184,76.285,56.432) | 0 | (53.396,77.172,56.676) | 0.944 | 1 |
| 458 | 2q9c | (45.778,75.619,51.383) | 0 | (48.365,73.659,51.266) | 3.248 | 0 |
| 459 | 2q9c | (43.856,68.851,60.745) | 0 | (48.676,66.354,62.033) | 5.579 | 0 |
| 460 | 2q9c | (53.890,73.904,56.204) | 0 | (53.94,74.462,56.403) | 0.595 | 1 |
| 461 | 2q9c | (53.927,62.998,53.333) | 1 | (53.626,62.638,53.127) | 0.512 | 1 |
| 462 | 2q9c | (49.560,74.783,53.690) | 0 | (49.04,74.73,53.228) | 0.698 | 1 |
| 463 | 2q9c | (46.187,65.943,53.530) | 1 | (47.603,65.987,51.362) | 2.590 | 0 |
| 464 | 2q9c | (48.422,61.442,50.910) | 0 | (48.592,62.238,50.266) | 1.038 | 1 |
| 465 | 2q9c | (45.844,63.554,53.994) | 0 | (45.49,61.278,53.1) | 2.471 | 0 |
| 466 | 2q9c | (53.647,61.215,55.694) | 0 | (53.098,60.538,55.807) | 0.879 | 1 |
| 467 | 2q9c | (45.727,69.436,61.929) | 0 | (48.987,67.06,62.669) | 4.101 | 0 |
| 468 | 2q9c | (48.402,68.144,50.073) | 1 | (49.23,68.028,49.75) | 0.896 | 1 |
| 469 | 2q9c | (48.604,65.026,50.454) | 0 | (49.656,64.522,50.342) | 1.172 | 1 |
| 470 | 2q9c | (52.079,63.386,48.638) | 0 | (52.063,63.255,48.713) | 0.152 | 1 |
| 471 | 2q9c | (46.755,60.032,54.504) | 0 | (47.139,59.741,54.758) | 0.545 | 1 |
| 472 | 2qu9 | (8.587,29.148,-7.044) | 0 | (8.826,28.545,-7.424) | 0.752 | 1 |
| 473 | 2qu9 | (6.181,23.769,-10.010) | 0 | (7.868,26.156,-9.759) | 2.934 | 0 |
| 474 | 2qu9 | (14.988,28.554,-7.122) | 1 | (12.22,26.027,-4.302) | 4.690 | 0 |
| 475 | 2rg5 | (-2.053,1.609,17.519) | 0 | (-1.817,2.568,18.413) | 1.332 | 1 |
| 476 | 2rg5 | (-1.949,3.430,19.823) | 0 | (-1.817,2.568,18.413) | 1.658 | 1 |
| 477 | 2rg5 | (2.075,0.501,20.945) | 0 | (1.098,-1.788,21.996) | 2.702 | 0 |
| 478 | 2rg5 | (1.804,-9.512,29.605) | 0 | (3.549,-12.597,25.051) | 5.771 | 0 |
| 479 | 2rg5 | (-0.693,8.509,14.141) | 0 | (2.245,8.341,14.436) | 2.958 | 0 |
| 480 | 2rhg | (44.575,28.751,9.636) | 1 | (44.68,28.907,9.514) | 0.224 | 1 |
| 481 | 2rhg | (46.208,32.496,8.616) | 1 | (45.869,32.64,9.167) | 0.663 | 1 |
| 482 | 2rhg | (55.179,28.145,20.387) | 1 | (55.288,28.473,20.399) | 0.346 | 1 |
| 483 | 2rhg | (54.968,29.332,17.901) | 1 | (54.985,29.06,17.232) | 0.722 | 1 |
| 484 | 2rhg | (57.654,29.272,19.365) | 1 | (57.38,29.319,18.846) | 0.589 | 1 |
| 485 | 2rhg | (51.711,17.596,15.646) | 1 | (52.193,17.299,17.633) | 2.066 | 0 |
| 486 | 2rhg | (51.431,17.554,12.730) | 1 | (50.129,14.863,16.309) | 4.663 | 0 |
| 487 | 2rhg | (52.690,20.331,11.982) | 1 | (54.186,24.764,9.739) | 5.189 | 0 |
| 488 | 2sli | (9.396,11.059,39.361) | 1 | (9.294,11.124,39.4) | 0.127 | 1 |
| 489 | 2sli | (6.333,10.970,38.109) | 1 | (8.812,10.634,38.674) | 2.565 | 0 |
| 490 | 2sli | (2.854,1.755,33.485) | 1 | (3.425,2.078,34.854) | 1.518 | 1 |
| 491 | 2sli | (8.138,4.306,41.615) | 1 | (5.62,2.851,42.721) | 3.111 | 0 |
| 492 | 2sli | (5.282,-0.189,41.303) | 1 | (6.737,1.73,43.123) | 3.019 | 0 |
| 493 | 2sli | (5.920,2.635,43.553) | 1 | (6.142,2.452,43.475) | 0.298 | 1 |
| 494 | 2sli | (-1.562,1.135,36.161) | 1 | (-1.177,1.02,34.969) | 1.258 | 1 |
| 495 | 2sli | (-2.150,11.782,42.659) | 1 | (-1.934,13.345,40.431) | 2.730 | 0 |
| 496 | 2sli | (1.876,10.518,44.278) | 1 | (1.338,10.241,44.275) | 0.605 | 1 |
| 497 | 2sli | (-0.848,8.680,40.886) | 0 | (1.381,10.579,43.335) | 3.817 | 0 |
| 498 | 2sli | (3.286,0.496,38.174) | 1 | (1.225,1.509,37.245) | 2.477 | 0 |
| 499 | 2sli | (5.666,9.002,42.440) | 1 | (1.381,10.579,43.335) | 4.653 | 0 |
| 500 | 2sli | (-3.352,10.244,40.688) | 0 | (-1.934,13.345,40.431) | 3.419 | 0 |
| 501 | 2sli | (3.499,8.276,44.161) | 1 | (1.338,10.241,44.275) | 2.923 | 0 |
| 502 | 2sli | (2.916,1.784,40.764) | 1 | (5.62,2.851,42.721) | 3.504 | 0 |
| 503 | 2sli | (0.763,1.813,35.209) | 1 | (-0.515,1.25,34.256) | 1.691 | 1 |
| 504 | 2sli | (1.956,3.790,36.533) | 0 | (1.928,3.987,36.035) | 0.536 | 1 |
| 505 | 2sli | (-5.209,8.448,41.939) | 0 | (-1.934,13.345,40.431) | 6.081 | 0 |
| 506 | 2uuo | (-27.433,8.069,16.382) | 1 | (-27.995,7.656,16.39) | 0.697 | 1 |
| 507 | 2uuo | (-21.049,8.990,16.752) | 1 | (-20.976,9.257,16.393) | 0.453 | 1 |
| 508 | 2uuo | (-27.119,8.160,19.369) | 1 | (-27.647,7.754,19.196) | 0.688 | 1 |
| 509 | 2uuo | (-26.079,10.730,16.764) | 1 | (-26.183,10.406,17.221) | 0.570 | 1 |
| 510 | 2uuo | (-23.742,8.591,15.620) | 1 | (-21.516,9.296,17.234) | 2.839 | 0 |
| 511 | 2uuo | (-14.093,17.143,17.487) | 1 | (-13.704,12.096,19.376) | 5.403 | 0 |
| 512 | 2uuo | (-14.246,20.630,11.704) | 1 | (-14.308,20.973,8.181) | 3.540 | 0 |
| 513 | 2uuo | (-29.659,9.080,13.499) | 1 | (-29.574,9.267,14.264) | 0.792 | 1 |
| 514 | 2uy4 | (39.670,16.154,21.033) | 0 | (37.622,17.151,23.063) | 3.051 | 0 |
| 515 | 2uy4 | (42.430,17.549,18.227) | 1 | (43.629,13.918,19.331) | 3.980 | 0 |
| 516 | 2uy4 | (41.496,22.401,18.375) | 1 | (42.304,22.417,16.968) | 1.623 | 1 |
| 517 | 2uy4 | (37.233,13.931,24.911) | 1 | (36.926,13.359,24.017) | 1.105 | 1 |
| 518 | 2uy4 | (37.655,16.428,22.969) | 1 | (37.622,17.151,23.063) | 0.730 | 1 |
| 519 | 2uy4 | (32.815,22.913,29.677) | 0 | (32.069,22.54,30.309) | 1.046 | 1 |
| 520 | 2uy4 | (35.355,23.279,24.217) | 0 | (36.271,22.973,24.355) | 0.976 | 1 |
| 521 | 2uy4 | (31.876,20.904,23.431) | 0 | (32.464,21.843,24.396) | 1.469 | 1 |
| 522 | 2uy4 | (39.306,25.585,29.843) | 0 | (39.407,25.595,29.632) | 0.234 | 1 |
| 523 | 2uy4 | (34.382,27.058,24.245) | 0 | (34.051,26.947,24.21) | 0.351 | 1 |
| 524 | 2uy4 | (40.721,27.173,28.086) | 1 | (41.519,27.836,27.597) | 1.147 | 1 |
| 525 | 2uy4 | (33.506,25.018,22.879) | 0 | (33.124,24.388,23.105) | 0.771 | 1 |
| 526 | 2uy4 | (39.118,25.202,14.921) | 0 | (41.491,23.215,15.798) | 3.217 | 0 |
| 527 | 2uyq | (-5.640,-18.247,-2.689) | 0 | (-6.04,-17.547,-1.656) | 1.310 | 1 |
| 528 | 2uyq | (-9.618,-23.758,-6.490) | 1 | (-8.231,-22.903,-3.637) | 3.285 | 0 |
| 529 | 2uyq | (-15.914,-23.763,-3.536) | 1 | (-15.323,-25.123,-2.013) | 2.126 | 0 |
| 530 | 2uyq | (-14.340,-29.185,-7.807) | 1 | (-14.132,-28.854,-7.378) | 0.580 | 1 |
| 531 | 2uyq | (-14.100,-27.694,-1.275) | 1 | (-14.013,-27.524,-0.996) | 0.338 | 1 |
| 532 | 2uyq | (-11.502,-28.983,-2.596) | 1 | (-10.365,-26.843,-1.668) | 2.595 | 0 |
| 533 | 2uyq | (-13.947,-25.153,-2.754) | 1 | (-14.585,-25.656,-2.426) | 0.876 | 1 |
| 534 | 2uyq | (1.752,-25.628,4.137) | 1 | (1.758,-24.774,3.17) | 1.290 | 1 |
| 535 | 2uyq | (1.289,-31.415,-6.584) | 1 | (0.926,-30.761,-6.758) | 0.768 | 1 |
| 536 | 2uyq | (-12.478,-28.256,-6.292) | 0 | (-13.43,-28.324,-6.903) | 1.133 | 1 |
| 537 | 2uyq | (-3.263,-29.411,-8.872) | 0 | (-3.899,-31.765,-11.289) | 3.433 | 0 |
| 538 | 2uyq | (-1.310,-31.033,-6.006) | 0 | (-0.041,-31.004,-6.68) | 1.437 | 1 |
| 539 | 2uyq | (-0.440,-28.714,-8.960) | 1 | (-0.041,-31.004,-6.68) | 3.256 | 0 |
| 540 | 2uyq | (-8.301,-22.580,-3.575) | 1 | (-8.231,-22.903,-3.637) | 0.336 | 1 |
| 541 | 2vbw | (4.283,9.751,3.351) | 1 | (3.801,10.025,3.631) | 0.621 | 1 |
| 542 | 2vbw | (3.930,7.067,0.314) | 0 | (3.544,6.587,1.181) | 1.064 | 1 |
| 543 | 2vbw | (-0.074,14.225,1.050) | 0 | (-2.059,17.267,1.293) | 3.640 | 0 |
| 544 | 2vdj | (-33.158,-7.409,2.163) | 0 | (-34.372,-8.589,1.642) | 1.771 | 1 |
| 545 | 2vdj | (-32.613,-9.953,2.679) | 0 | (-32.492,-10.886,3.182) | 1.067 | 1 |
| 546 | 2vdj | (-28.476,-5.170,11.998) | 1 | (-28.291,-4.599,11.054) | 1.119 | 1 |
| 547 | 2vdj | (-26.565,-3.274,9.085) | 0 | (-26.422,-4.75,9.11) | 1.483 | 1 |
| 548 | 2vdj | (-26.201,-1.250,12.076) | 0 | (-27.378,-4.202,11.148) | 3.311 | 0 |
| 549 | 2vdj | (-33.323,-1.134,2.038) | 1 | (-33.589,-4.599,0.617) | 3.754 | 0 |
| 550 | 2vdj | (-24.996,-8.177,11.847) | 1 | (-25.398,-8.127,11.952) | 0.418 | 1 |
| 551 | 2vdj | (-26.751,0.161,7.460) | 1 | (-27.582,0.448,7.729) | 0.919 | 1 |
| 552 | 2vdj | (-23.308,-9.275,6.363) | 1 | (-23.17,-9.415,6.215) | 0.246 | 1 |
| 553 | 2vdj | (-23.024,-10.876,10.622) | 1 | (-22.921,-11.015,10.461) | 0.236 | 1 |
| 554 | 2vdj | (-32.772,-3.465,0.717) | 0 | (-33.589,-4.599,0.617) | 1.401 | 1 |
| 555 | 2vdj | (-31.975,-5.067,2.819) | 1 | (-31.693,-5.856,3.104) | 0.885 | 1 |
| 556 | 2vdj | (-30.376,2.443,8.009) | 0 | (-30.486,1.727,7.894) | 0.733 | 1 |
| 557 | 2vdj | (-25.034,3.212,7.358) | 0 | (-24.976,3.599,6.722) | 0.747 | 1 |
| 558 | 2vdj | (-26.012,0.671,3.720) | 1 | (-26.333,0.554,3.669) | 0.345 | 1 |
| 559 | 2vdj | (-27.079,-7.058,10.695) | 0 | (-25.724,-8.035,11.011) | 1.700 | 1 |
| 560 | 2vdj | (-28.614,-8.963,10.148) | 1 | (-28.805,-8.338,10.052) | 0.661 | 1 |
| 561 | 2vk6 | (-0.784,36.850,25.527) | 1 | / | / | / |
| 562 | 2vk6 | (6.073,39.240,24.510) | 0 | / | / | / |
| 563 | 2vk6 | (7.813,38.135,26.254) | 0 | / | / | / |
| 564 | 2vk6 | (9.814,36.428,25.329) | 0 | / | / | / |
| 565 | 2vk6 | (6.287,28.926,18.889) | 1 | / | / | / |
| 566 | 2vk6 | (4.123,31.580,17.703) | 1 | / | / | / |
| 567 | 2vk6 | (11.980,40.564,15.594) | 1 | / | / | / |
| 568 | 2vk6 | (4.984,37.255,14.910) | 1 | / | / | / |
| 569 | 2vk6 | (6.969,40.420,17.233) | 0 | / | / | / |
| 570 | 2vk6 | (-2.167,42.415,16.221) | 1 | / | / | / |
| 571 | 2vk6 | (-3.411,38.640,22.203) | 1 | / | / | / |
| 572 | 2vk6 | (-5.502,36.594,22.271) | 1 | / | / | / |
| 573 | 2vk6 | (4.058,38.485,21.320) | 1 | / | / | / |
| 574 | 2vk6 | (7.993,33.429,24.245) | 1 | / | / | / |
| 575 | 2w6p | (8.051,-27.360,19.169) | 0 | (8.453,-27.376,18.41) | 0.859 | 1 |
| 576 | 2w6p | (7.015,-29.864,22.122) | 1 | (6.836,-29.55,22.162) | 0.364 | 1 |
| 577 | 2w6p | (-0.455,-20.931,27.175) | 1 | (-2.672,-19.482,27.626) | 2.687 | 0 |
| 578 | 2w6p | (10.082,-17.699,27.277) | 1 | (10.904,-18.606,25.326) | 2.303 | 0 |
| 579 | 2w6p | (10.343,-20.005,23.001) | 0 | (10.665,-19.848,23.394) | 0.532 | 1 |
| 580 | 2w6p | (11.527,-18.073,24.524) | 0 | (11.208,-17.886,24.702) | 0.410 | 1 |
| 581 | 2w6p | (10.271,-22.669,23.326) | 0 | (10.825,-22.595,22.983) | 0.656 | 1 |
| 582 | 2w6p | (6.180,-32.912,25.069) | 1 | (5.39,-33.81,24.646) | 1.269 | 1 |
| 583 | 2w6p | (7.393,-26.364,21.616) | 1 | (7.18,-25.904,22.253) | 0.814 | 1 |
| 584 | 2w6p | (3.742,-12.390,27.481) | 1 | (3.848,-12.186,27.848) | 0.433 | 1 |
| 585 | 2w6p | (3.635,-15.399,23.534) | 1 | (4.635,-17.096,23.855) | 1.996 | 1 |
| 586 | 2xda | (5.329,15.765,21.514) | 1 | (5.152,15.789,21.617) | 0.206 | 1 |
| 587 | 2xda | (4.654,22.965,23.314) | 1 | (6.991,21.433,23.662) | 2.816 | 0 |
| 588 | 2xda | (-2.660,19.957,29.212) | 0 | (-2.638,19.803,29.203) | 0.156 | 1 |
| 589 | 2xda | (-4.998,12.762,28.538) | 0 | (-4.377,13.272,30.139) | 1.791 | 1 |
| 590 | 2xda | (-0.869,16.243,20.436) | 0 | (4.223,15.534,21.884) | 5.341 | 0 |
| 591 | 2xda | (-3.640,13.541,30.475) | 0 | (-3.447,13.411,30.479) | 0.233 | 1 |
| 592 | 2xda | (-1.147,6.985,30.980) | 1 | (-1.201,7.293,30.989) | 0.313 | 1 |
| 593 | 2xda | (-5.785,10.249,28.837) | 1 | (-4.377,13.272,30.139) | 3.580 | 0 |
| 594 | 2xda | (-6.724,8.757,24.877) | 1 | (-6.499,8.866,23.184) | 1.711 | 1 |
| 595 | 2xgs | (-15.096,11.370,26.596) | 1 | (-15.311,11.259,26.394) | 0.315 | 1 |
| 596 | 2xgs | (-25.152,10.708,29.957) | 1 | (-22.583,9.56,31.088) | 3.033 | 0 |
| 597 | 2ypi | (95.875,39.951,46.767) | 0 | (81.207,39.803,45.224) | 14.75 | 0 |
| 598 | 2ypi | (52.365,38.542,56.413) | 0 | (51.817,38.556,56.195) | 0.590 | 1 |
| 599 | 2ypi | (52.621,41.397,53.173) | 0 | (52.659,41.1,52.627) | 0.623 | 1 |
| 600 | 2zdv | (6.950,8.955,-0.245) | 0 | (6.597,6.518,-0.437) | 2.470 | 0 |
| 601 | 2zdv | (22.164,1.487,3.180) | 1 | (23.954,1.352,3.184) | 1.795 | 1 |
| 602 | 2zdv | (23.668,2.547,0.126) | 0 | (24.876,0.891,-1.317) | 2.507 | 0 |
| 603 | 2zdv | (12.664,12.604,2.994) | 1 | (14.933,12.028,2.867) | 2.344 | 0 |
| 604 | 2zdv | (6.254,5.164,-6.660) | 0 | (7.304,6.201,-1.068) | 5.783 | 0 |
| 605 | 2zjl | (82.708,30.448,19.798) | 1 | (82.947,31.569,19.193) | 1.296 | 1 |
| 606 | 2zjl | (78.989,36.077,28.487) | 1 | (78.751,36.006,28.986) | 0.557 | 1 |
| 607 | 2zjl | (73.241,27.429,24.531) | 1 | (73.066,27.421,24.364) | 0.242 | 1 |
| 608 | 2zjl | (76.487,39.309,24.942) | 0 | (75.855,38.679,25.08) | 0.903 | 1 |
| 609 | 2zjl | (82.130,31.592,17.323) | 1 | (82.359,31.193,18.477) | 1.242 | 1 |
| 610 | 2zjl | (70.757,39.342,21.511) | 0 | (70.684,39.363,23.084) | 1.575 | 1 |
| 611 | 2zjl | (80.362,41.809,19.038) | 0 | (80.424,40.538,14.355) | 4.853 | 0 |
| 612 | 2zjl | (75.578,38.263,27.557) | 0 | (75.563,38.495,26.676) | 0.911 | 1 |
| 613 | 2zjl | (81.017,29.378,16.353) | 1 | (80.409,28.732,16.738) | 0.967 | 1 |
| 614 | 2zjl | (71.380,36.355,20.290) | 1 | (70.684,39.363,23.084) | 4.164 | 0 |
| 615 | 2zjl | (76.524,29.200,17.845) | 0 | (75.445,29.334,17.593) | 1.116 | 1 |
| 616 | 2zjl | (75.600,40.737,28.553) | 1 | (76.562,41.674,30.508) | 2.372 | 0 |
| 617 | 2zjl | (74.569,38.484,21.308) | 0 | (75.844,38.895,21.512) | 1.355 | 1 |
| 618 | 2zjl | (77.303,31.385,16.411) | 0 | (76.205,32.013,14.166) | 2.577 | 0 |
| 619 | 2zjl | (73.528,37.685,14.635) | 0 | (75.608,32.553,14.759) | 5.539 | 0 |
| 620 | 2zjl | (78.522,38.763,12.605) | 0 | (80.553,39.614,13.995) | 2.604 | 0 |
| 621 | 2zjl | (82.579,42.828,27.764) | 1 | (82.28,42.209,27.722) | 0.689 | 1 |
| 622 | 2zjl | (78.775,27.898,17.411) | 1 | (79.658,28.116,16.976) | 1.008 | 1 |
| 623 | 2zpu | (12.286,-9.008,12.522) | 0 | (12.455,-8.409,11.056) | 1.593 | 1 |
| 624 | 2zpu | (11.332,1.833,12.523) | 1 | (11.519,1.688,12.878) | 0.427 | 1 |
| 625 | 2zpu | (11.691,-6.244,17.820) | 1 | (11.695,-8.264,17.378) | 2.068 | 0 |
| 626 | 2zpu | (12.802,-4.648,11.515) | 0 | (12.738,-4.659,11.352) | 0.175 | 1 |
| 627 | 2zpu | (17.280,0.858,10.469) | 1 | (12.235,2.384,12.931) | 5.817 | 0 |
| 628 | 2zpu | (15.984,-5.873,5.992) | 1 | (15.484,-5.015,5.591) | 1.071 | 1 |
| 629 | 2zpu | (11.065,-6.522,12.539) | 0 | (9.802,-5.634,11.874) | 1.681 | 1 |
| 630 | 2zpu | (8.191,3.054,16.253) | 1 | (8.253,3.181,16.291) | 0.146 | 1 |
| 631 | 2zpu | (8.671,-5.484,12.179) | 0 | (9.066,-6.076,12.387) | 0.741 | 1 |
| 632 | 2zpu | (9.985,-10.154,11.808) | 1 | (9.152,-10.768,11.587) | 1.058 | 1 |
| 633 | 2zpu | (6.752,-0.793,7.993) | 1 | (6.63,-0.849,7.909) | 0.158 | 1 |
| 634 | 2zpu | (16.991,-8.774,19.245) | 1 | (17.143,-8.983,19.271) | 0.260 | 1 |
| 635 | 3a1t | (23.901,24.643,29.368) | 1 | (27.33,25.387,26.681) | 4.419 | 0 |
| 636 | 3a1t | (24.626,23.099,21.248) | 1 | (24.59,23.014,21.236) | 0.093 | 1 |
| 637 | 3a1t | (28.850,23.495,28.430) | 0 | (27.33,25.387,26.681) | 2.991 | 0 |
| 638 | 3a1t | (25.983,22.855,28.523) | 0 | (27.33,25.387,26.681) | 3.409 | 0 |
| 639 | 3a1t | (20.751,16.482,27.867) | 0 | (20.752,15.199,27.644) | 1.302 | 1 |
| 640 | 3a1t | (22.433,14.856,25.798) | 1 | (23.538,13.685,24.496) | 2.071 | 0 |
| 641 | 3a1t | (24.382,12.280,24.947) | 1 | (23.41,13.074,25.277) | 1.298 | 1 |
| 642 | 3a1t | (23.892,13.779,22.853) | 1 | (24.047,13.208,23.78) | 1.100 | 1 |
| 643 | 3a1t | (26.904,12.490,25.695) | 0 | (24.047,13.208,23.78) | 3.514 | 0 |
| 644 | 3a1t | (32.093,16.942,26.508) | 0 | (29.481,20.574,20.509) | 7.483 | 0 |
| 645 | 3a1t | (33.785,29.538,19.334) | 0 | (33.059,23.692,19.111) | 5.895 | 0 |
| 646 | 3a1t | (31.451,11.881,27.038) | 0 | (25.417,7.068,25.427) | 7.885 | 0 |
| 647 | 3a1t | (22.654,10.069,26.726) | 1 | (23.41,13.074,25.277) | 3.421 | 0 |
| 648 | 3a1t | (25.680,13.907,27.637) | 0 | (23.41,13.074,25.277) | 3.379 | 0 |
| 649 | 3a1t | (28.186,14.390,26.698) | 0 | (23.41,13.074,25.277) | 5.154 | 0 |
| 650 | 3a1t | (29.439,20.190,18.990) | 1 | (28.926,20.148,19.795) | 0.955 | 1 |
| 651 | 3a1t | (31.427,17.167,29.123) | 0 | (27.64,24.787,25.944) | 9.084 | 0 |
| 652 | 3a1t | (29.656,16.239,26.998) | 0 | (23.538,13.685,24.496) | 7.086 | 0 |
| 653 | 3a1t | (33.173,14.015,26.931) | 0 | (29.536,13.426,19.534) | 8.264 | 0 |
| 654 | 3a1t | (27.645,16.139,28.448) | 0 | (23.41,13.074,25.277) | 6.114 | 0 |
| 655 | 3a1t | (31.484,20.228,29.477) | 0 | (27.64,24.787,25.944) | 6.931 | 0 |
| 656 | 3bmy | (-0.066,0.416,12.138) | 0 | (0.358,0.499,12.102) | 0.434 | 1 |
| 657 | 3bmy | (0.967,-0.820,14.740) | 1 | (0.451,1.278,12.722) | 2.956 | 0 |
| 658 | 3bmy | (9.850,4.001,18.730) | 1 | (10.248,4.704,18.583) | 0.821 | 1 |
| 659 | 3bmy | (2.447,1.343,11.091) | 1 | (2.328,2.026,10.849) | 0.734 | 1 |
| 660 | 3bmy | (1.136,4.277,12.122) | 0 | (-0.371,3.245,11.296) | 2.005 | 0 |
| 661 | 3bmy | (5.935,7.155,22.361) | 1 | (9.457,5.312,18.653) | 5.436 | 0 |
| 662 | 3bmy | (-2.114,10.309,17.065) | 1 | (-0.74,16.174,17.862) | 6.076 | 0 |
| 663 | 3bmy | (1.178,8.299,10.742) | 1 | (2.325,6.508,7.952) | 3.508 | 0 |
| 664 | 3bmy | (8.602,2.889,16.380) | 1 | (8.977,3.026,16.354) | 0.400 | 1 |
| 665 | 3bmy | (-0.509,5.945,25.470) | 1 | (-1.26,7.1,27.46) | 2.420 | 0 |
| 666 | 3bmy | (-2.084,2.420,11.640) | 0 | (-1.88,3.315,10.676) | 1.331 | 1 |
| 667 | 3bmy | (1.148,2.570,8.282) | 0 | (1.977,2.29,8.392) | 0.882 | 1 |
| 668 | 3bmy | (-0.562,4.417,9.738) | 0 | (-1.88,3.315,10.676) | 1.957 | 1 |
| 669 | 3bmy | (-1.266,-5.074,18.857) | 0 | (-2.546,-5.177,19.958) | 1.692 | 1 |
| 670 | 3brf | (-27.824,-19.733,42.883) | 0 | (-28.027,-21.181,45.694) | 3.169 | 0 |
| 671 | 3brf | (-33.896,-20.515,46.924) | 0 | (-33.338,-20.322,46.459) | 0.752 | 1 |
| 672 | 3brf | (-30.920,-15.137,49.415) | 0 | (-31.227,-15.109,49.42) | 0.308 | 1 |
| 673 | 3d6o | (38.314,3.712,12.525) | 0 | (39.483,4.634,10.464) | 2.543 | 0 |
| 674 | 3d6o | (37.718,-0.606,21.353) | 1 | (37.725,-0.834,21.444) | 0.246 | 1 |
| 675 | 3d6o | (34.413,5.988,12.677) | 1 | (34.564,6.107,13.624) | 0.966 | 1 |
| 676 | 3d6o | (40.894,-1.743,9.688) | 0 | (39.849,-1.291,9.944) | 1.167 | 1 |
| 677 | 3d6o | (35.328,3.056,13.680) | 1 | (34.699,5.158,13.341) | 2.220 | 0 |
| 678 | 3d6o | (33.964,-7.998,14.569) | 0 | (31.496,-7.096,11.904) | 3.743 | 0 |
| 679 | 3d6o | (33.464,-10.229,16.806) | 0 | (31.496,-7.096,11.904) | 6.142 | 0 |
| 680 | 3d6o | (37.598,-3.008,16.232) | 0 | (36.425,-1.316,17.448) | 2.391 | 0 |
| 681 | 3d6o | (31.936,-6.792,11.472) | 0 | (31.854,-6.287,11.438) | 0.513 | 1 |
| 682 | 3d6o | (36.539,1.953,21.343) | 1 | (35.531,1.089,21.452) | 1.332 | 1 |
| 683 | 3d6o | (39.881,-0.811,19.757) | 1 | (37.725,-0.834,21.444) | 2.738 | 0 |
| 684 | 3d6o | (36.572,5.295,14.272) | 0 | (34.699,5.158,13.341) | 2.096 | 0 |
| 685 | 3d6o | (43.723,-6.365,10.210) | 0 | (40.963,-1.398,11.133) | 5.757 | 0 |
| 686 | 3d6o | (38.375,3.724,20.197) | 0 | (35.691,1.116,20.465) | 3.752 | 0 |
| 687 | 3d6o | (39.232,-5.789,13.934) | 0 | (40.963,-1.398,11.133) | 5.488 | 0 |
| 688 | 3d6o | (39.693,4.921,14.308) | 0 | (40.242,4.954,11.031) | 3.323 | 0 |
| 689 | 3d6o | (40.917,3.180,12.622) | 0 | (40.242,4.954,11.031) | 2.477 | 0 |
| 690 | 3d6o | (39.918,5.030,10.788) | 0 | (40.242,4.954,11.031) | 0.412 | 1 |
| 691 | 3d6o | (41.721,5.789,12.683) | 0 | (40.242,4.954,11.031) | 2.369 | 0 |
| 692 | 3dwz | (-9.651,-0.238,-2.101) | 1 | (-8.832,-0.362,-2.214) | 0.836 | 1 |
| 693 | 3dwz | (-11.111,4.463,-9.070) | 1 | (-11.494,4.238,-8.784) | 0.528 | 1 |
| 694 | 3dwz | (-9.752,16.272,-2.407) | 0 | (-10,16.148,-1.86) | 0.613 | 1 |
| 695 | 3dwz | (-9.803,3.739,-2.902) | 1 | (-9.003,0.312,-2.932) | 3.519 | 0 |
| 696 | 3dwz | (-6.550,0.807,-0.184) | 1 | (-9.021,0.047,-1.321) | 2.824 | 0 |
| 697 | 3dwz | (-13.426,13.499,-5.935) | 1 | (-10.738,15.48,-1.954) | 5.196 | 0 |
| 698 | 3dwz | (-9.097,4.489,2.154) | 1 | (-8.56,5.045,3.575) | 1.618 | 1 |
| 699 | 3dwz | (-8.155,6.897,2.872) | 0 | (-8.56,5.045,3.575) | 2.022 | 0 |
| 700 | 3dwz | (-11.045,13.764,-1.661) | 0 | (-10.738,15.48,-1.954) | 1.768 | 1 |
| 701 | 3dwz | (-3.833,3.271,-7.073) | 1 | (-4.385,2.677,-6.402) | 1.053 | 1 |
| 702 | 3dwz | (-5.088,3.041,-4.514) | 1 | (-4.385,2.677,-6.402) | 2.047 | 0 |
| 703 | 3dwz | (0.938,5.893,-10.440) | 0 | (1.173,6.862,-13.902) | 3.603 | 0 |
| 704 | 3dwz | (-6.660,8.577,-0.195) | 0 | (-8.56,5.045,3.575) | 5.504 | 0 |
| 705 | 3dwz | (-9.698,11.767,3.562) | 1 | (-10.143,14.377,1.973) | 3.088 | 0 |
| 706 | 3dwz | (-9.859,13.678,0.961) | 1 | (-10.571,14.701,1.129) | 1.258 | 1 |
| 707 | 3e6p | (-55.557,-22.315,-10.387) | 1 | (-55.476,-22.306,-10.806) | 0.427 | 1 |
| 708 | 3e6p | (-52.766,-22.975,-9.486) | 1 | (-52.449,-22.648,-9.073) | 0.615 | 1 |
| 709 | 3e6p | (-55.266,-21.792,-12.890) | 1 | (-54.845,-21.584,-11.091) | 1.859 | 1 |
| 710 | 3e6p | (-57.340,-20.676,-12.576) | 1 | (-54.845,-21.584,-11.091) | 3.042 | 0 |
| 711 | 3e6p | (-49.898,-22.251,-11.040) | 1 | (-49.851,-22.354,-10.962) | 0.137 | 1 |
| 712 | 3e6p | (-55.218,-22.432,-15.547) | 1 | (-55.787,-23.053,-15.663) | 0.850 | 1 |
| 713 | 3e6p | (-55.734,-23.904,-17.641) | 1 | (-55.266,-25.217,-16.636) | 1.718 | 1 |
| 714 | 3e6p | (-54.994,-14.484,-19.902) | 1 | (-57.058,-14.82,-17.329) | 3.316 | 0 |
| 715 | 3e6p | (-54.825,-17.016,-16.549) | 1 | (-54.872,-16.789,-16.646) | 0.251 | 1 |
| 716 | 3e6p | (-58.426,-16.007,-18.490) | 1 | (-57.991,-15.147,-17.178) | 1.628 | 1 |
| 717 | 3e6p | (-48.732,-20.038,-14.959) | 1 | (-49.83,-22.655,-11.915) | 4.162 | 0 |
| 718 | 3e6p | (-54.020,-17.386,-29.560) | 1 | (-52.188,-19.027,-29.197) | 2.486 | 0 |
| 719 | 3e6p | (-59.675,-20.554,-26.166) | 0 | (-58.765,-24.56,-23.206) | 5.063 | 0 |
| 720 | 3e6p | (-57.385,-15.124,-23.991) | 1 | (-57.058,-14.82,-17.329) | 6.677 | 0 |
| 721 | 3e6p | (-59.040,-17.599,-21.029) | 1 | (-57.991,-15.147,-17.178) | 4.684 | 0 |
| 722 | 3e6p | (-58.922,-16.966,-23.209) | 0 | (-57.991,-15.147,-17.178) | 6.368 | 0 |
| 723 | 3e6p | (-58.071,-14.189,-15.880) | 1 | (-58.103,-15.403,-16.218) | 1.261 | 1 |
| 724 | 3e6p | (-49.908,-10.581,-18.773) | 0 | (-49.007,-10.67,-19.099) | 0.962 | 1 |
| 725 | 3e6p | (-51.920,-11.047,-19.804) | 1 | (-49.007,-10.67,-19.099) | 3.021 | 0 |
| 726 | 3e6p | (-60.559,-17.811,-19.160) | 0 | (-57.991,-15.147,-17.178) | 4.198 | 0 |
| 727 | 3e6p | (-60.599,-22.787,-24.652) | 0 | (-59.754,-24.657,-23.092) | 2.578 | 0 |
| 728 | 3fdz | (18.912,-36.082,53.242) | 1 | (18.963,-35.964,53.047) | 0.234 | 1 |
| 729 | 3fdz | (20.182,-23.717,61.255) | 1 | (19.794,-23.924,61.401) | 0.463 | 1 |
| 730 | 3fdz | (19.347,-36.311,59.427) | 1 | (19.725,-35.938,59.503) | 0.536 | 1 |
| 731 | 3fdz | (17.922,-28.255,66.578) | 1 | (13.923,-30.68,65.674) | 4.763 | 0 |
| 732 | 3fdz | (27.214,-36.021,60.783) | 0 | (27.365,-36.138,60.817) | 0.194 | 1 |
| 733 | 3fdz | (21.564,-33.596,66.192) | 0 | (22.881,-34.317,67.496) | 1.989 | 1 |
| 734 | 3fdz | (16.800,-32.869,65.342) | 0 | (14.846,-34.386,66.261) | 2.639 | 0 |
| 735 | 3fdz | (14.461,-29.774,65.128) | 1 | (13.461,-29.797,65.59) | 1.102 | 1 |
| 736 | 3ftu | (23.427,-0.236,-8.324) | 1 | (23.4,-0.121,-8.471) | 0.189 | 1 |
| 737 | 3ftu | (23.885,3.148,6.323) | 0 | (24.405,2.847,6.222) | 0.609 | 1 |
| 738 | 3ftu | (25.996,2.709,4.342) | 0 | (26.185,2.711,4.553) | 0.283 | 1 |
| 739 | 3ftu | (32.717,0.947,-4.278) | 0 | (32.714,0.908,-4.457) | 0.183 | 1 |
| 740 | 3ftu | (30.295,-0.285,6.799) | 0 | (29.131,-0.332,5.489) | 1.753 | 1 |
| 741 | 3ftu | (26.169,-4.902,4.231) | 0 | (26.385,-4.824,4.335) | 0.252 | 1 |
| 742 | 3ftu | (21.713,-4.232,-7.188) | 0 | (22.909,-0.991,-8.514) | 3.700 | 0 |
| 743 | 3ftu | (21.073,-6.330,-3.312) | 0 | (21.01,-6.357,-3.423) | 0.130 | 1 |
| 744 | 3ftu | (28.681,-0.963,5.056) | 0 | (28.24,-0.339,5.036) | 0.764 | 1 |
| 745 | 3ftu | (33.605,-3.165,0.628) | 0 | (33.774,-2.762,1.025) | 0.590 | 1 |
| 746 | 3ftu | (33.860,-1.159,-1.751) | 0 | (33.487,-1.223,-2.789) | 1.105 | 1 |
| 747 | 3gcq | (-2.326,-6.674,-17.360) | 0 | (-4.901,-5.136,-16.374) | 3.157 | 0 |
| 748 | 3gcq | (-3.725,4.846,-15.698) | 0 | (-3.115,4.735,-15.963) | 0.674 | 1 |
| 749 | 3gcq | (-6.746,-11.858,-13.760) | 1 | (-5.462,-9.99,-13.026) | 2.383 | 0 |
| 750 | 3gcq | (-5.102,-9.812,-13.084) | 1 | (-5.462,-9.99,-13.026) | 0.406 | 1 |
| 751 | 3gcq | (-4.255,-4.909,-22.050) | 0 | (-4.577,-8.067,-22.567) | 3.216 | 0 |
| 752 | 3gcq | (1.053,5.865,-25.056) | 0 | (1.399,5.633,-25.022) | 0.418 | 1 |
| 753 | 3gcq | (-5.146,-8.887,-16.701) | 0 | (-5.131,-8.903,-16.386) | 0.316 | 1 |
| 754 | 3gcq | (-7.464,-0.711,-18.407) | 0 | (-4.901,-5.136,-16.374) | 5.503 | 0 |
| 755 | 3gcq | (-3.332,-4.878,-25.573) | 0 | (-4.481,-5.731,-25.839) | 1.456 | 1 |
| 756 | 3gcq | (3.462,-7.264,-11.703) | 0 | (4.919,-8.441,-12.465) | 2.022 | 0 |
| 757 | 3gcq | (-1.348,-6.018,-27.029) | 0 | (-4.481,-5.731,-25.839) | 3.364 | 0 |
| 758 | 3gcq | (3.669,-8.057,-6.114) | 0 | (3.347,-8.036,-6.414) | 0.441 | 1 |
| 759 | 3gcq | (-0.743,-7.737,-25.021) | 0 | (-4.481,-5.731,-25.839) | 4.320 | 0 |
| 760 | 3gcq | (6.133,-9.534,-14.082) | 0 | (5.874,-9.724,-13.949) | 0.348 | 1 |
| 761 | 3gcq | (5.467,-7.996,-10.418) | 0 | (6.363,-7.879,-9.537) | 1.262 | 1 |
| 762 | 3gry | (25.442,20.006,13.552) | 0 | (25.574,19.535,13.059) | 0.694 | 1 |
| 763 | 3gry | (16.647,20.027,17.899) | 1 | (16.397,15.059,19.321) | 5.174 | 0 |
| 764 | 3gry | (24.028,29.456,12.466) | 0 | (22.499,28.547,10.345) | 2.768 | 0 |
| 765 | 3gry | (26.379,32.067,18.817) | 0 | (25.378,26.725,16.475) | 5.918 | 0 |
| 766 | 3gry | (26.509,28.953,17.547) | 1 | (26.971,26.818,16.129) | 2.604 | 0 |
| 767 | 3gry | (24.913,16.925,18.810) | 1 | (24.568,17.187,18.631) | 0.469 | 1 |
| 768 | 3hv7 | (-2.038,-6.932,-17.348) | 0 | (-2.078,-6.614,-16.93) | 0.527 | 1 |
| 769 | 3hv7 | (-4.783,-9.641,-12.749) | 0 | (-5.075,-9.778,-12.761) | 0.323 | 1 |
| 770 | 3hv7 | (0.888,-7.199,-5.045) | 0 | (0.697,-7.201,-5.54) | 0.531 | 1 |
| 771 | 3hv7 | (3.897,-6.949,-12.298) | 0 | (5.513,-6.486,-13.461) | 2.044 | 0 |
| 772 | 3hv7 | (2.868,-9.530,-11.607) | 0 | (5.934,-8.275,-10.851) | 3.398 | 0 |
| 773 | 3hv7 | (1.139,-11.876,-11.586) | 0 | (2.098,-9.783,-15.987) | 4.967 | 0 |
| 774 | 3i81 | (-8.882,-2.764,16.077) | 1 | (-3.453,-11.608,23.234) | 12.606 | 0 |
| 775 | 3i81 | (-12.124,8.149,10.182) | 0 | (-21.169,8.94,8.136) | 9.307 | 0 |
| 776 | 3i81 | (-12.210,5.335,10.050) | 0 | (-21.169,8.94,8.136) | 9.845 | 0 |
| 777 | 3i81 | (-8.506,3.554,9.903) | 0 | (-3.418,-2.067,3.036) | 10.229 | 0 |
| 778 | 3i81 | (-9.334,-2.228,11.669) | 0 | (-16.997,-4.321,4.963) | 10.396 | 0 |
| 779 | 3i81 | (-6.274,-4.510,11.082) | 0 | (-2.551,-6.899,4.842) | 7.649 | 0 |
| 780 | 3i81 | (-7.700,-4.047,13.812) | 0 | (-2.551,-6.899,4.842) | 10.729 | 0 |
| 781 | 3i81 | (-18.494,13.891,14.727) | 0 | (-22.086,10.074,8.872) | 7.858 | 0 |
| 782 | 3i81 | (-12.572,12.965,13.247) | 0 | (-21.375,9.917,8.186) | 10.602 | 0 |
| 783 | 3l5t | (16.993,-2.808,5.247) | 0 | (17.106,-2.565,4.842) | 0.486 | 1 |
| 784 | 3l5t | (4.156,-4.498,-0.020) | 1 | (5.777,-11.706,-1.744) | 7.587 | 0 |
| 785 | 3l5t | (3.609,-1.656,-1.415) | 1 | (5.28,-2.567,8.114) | 9.717 | 0 |
| 786 | 3lkh | (23.491,42.978,54.653) | 1 | (23.259,42.029,54.403) | 1.008 | 1 |
| 787 | 3lkh | (23.542,40.175,54.936) | 1 | (23.5,41.595,55.271) | 1.460 | 1 |
| 788 | 3lkh | (15.986,35.316,51.546) | 1 | (15.92,34.823,51.419) | 0.513 | 1 |
| 789 | 3lkh | (16.730,34.019,43.057) | 0 | (15.722,32.405,40.993) | 2.807 | 0 |
| 790 | 3lkh | (30.060,39.540,49.743) | 1 | (30.68,39.346,49.986) | 0.694 | 1 |
| 791 | 3lkh | (25.264,37.237,45.558) | 1 | (25.487,36.1,45.833) | 1.191 | 1 |
| 792 | 3lkh | (17.039,42.215,36.515) | 0 | (16.917,42.052,36.463) | 0.210 | 1 |
| 793 | 3lkh | (27.479,47.729,47.925) | 1 | (26.142,51.032,48.24) | 3.577 | 0 |
| 794 | 3lkh | (26.594,42.482,43.080) | 0 | (25.716,42.366,43.531) | 0.994 | 1 |
| 795 | 3lkh | (26.967,46.028,45.694) | 1 | (27.38,44.799,45.325) | 1.348 | 1 |
| 796 | 3m6h | (-11.113,-4.574,9.094) | 1 | (-11.532,-4.249,8.888) | 0.569 | 1 |
| 797 | 3m6h | (-6.213,-18.466,1.647) | 1 | (-10.051,-16.634,1.972) | 4.265 | 0 |
| 798 | 3m6h | (-7.146,-16.158,3.237) | 1 | (-10.051,-16.634,1.972) | 3.204 | 0 |
| 799 | 3m6h | (-9.652,0.193,2.279) | 1 | (-8.743,0.383,2.331) | 0.930 | 1 |
| 800 | 3m6h | (-9.816,-16.409,2.251) | 0 | (-10.051,-16.634,1.972) | 0.429 | 1 |
| 801 | 3m6h | (-9.722,-3.819,3.184) | 1 | (-9.129,-3.251,2.749) | 0.929 | 1 |
| 802 | 3m6h | (-13.460,-13.830,5.912) | 1 | (-13.376,-13.753,5.912) | 0.114 | 1 |
| 803 | 3m6h | (-3.781,-3.254,7.164) | 1 | (-7.589,-1.703,1.964) | 6.629 | 0 |
| 804 | 3m6h | (-4.953,-2.828,4.478) | 1 | (-7.589,-1.703,1.964) | 3.812 | 0 |
| 805 | 3m6h | (-10.923,-13.774,1.684) | 0 | (-10.521,-15.805,2.274) | 2.153 | 0 |
| 806 | 3m6h | (-9.043,-12.818,-1.524) | 0 | (-10.034,-14.363,-2.033) | 1.905 | 1 |
| 807 | 3m6h | (-9.368,-11.565,-3.650) | 0 | (-10.034,-14.363,-2.033) | 3.300 | 1 |
| 808 | 3n76 | (-33.139,-64.625,-44.165) | 0 | (-33.136,-64.93,-44.338) | 0.351 | 1 |
| 809 | 3n76 | (-42.321,-63.625,-46.047) | 1 | (-43.166,-63.416,-46.333) | 0.916 | 1 |
| 810 | 3n76 | (-41.586,-57.055,-47.161) | 1 | (-42.112,-56.662,-47.06) | 0.664 | 1 |
| 811 | 3n76 | (-37.551,-69.682,-53.181) | 1 | (-37.8,-69.377,-50.185) | 3.022 | 0 |
| 812 | 3n76 | (-33.717,-68.495,-52.701) | 1 | (-36.971,-68.893,-49.905) | 4.309 | 0 |
| 813 | 3n76 | (-38.282,-56.056,-56.119) | 1 | (-37.799,-56.377,-56.167) | 0.582 | 1 |
| 814 | 3n76 | (-40.596,-66.981,-44.008) | 0 | (-40.65,-65.169,-45.217) | 2.179 | 0 |
| 815 | 3n76 | (-37.297,-69.498,-50.230) | 0 | (-37.8,-69.377,-50.185) | 0.519 | 1 |
| 816 | 3n76 | (-43.676,-60.635,-58.227) | 1 | (-43.173,-60.598,-59.003) | 0.926 | 1 |
| 817 | 3n76 | (-35.921,-66.924,-37.919) | 0 | (-33.505,-65.074,-43.42) | 6.287 | 0 |
| 818 | 3pbo | (8.774,-4.853,17.970) | 0 | (10.56,-0.907,17.243) | 4.392 | 0 |
| 819 | 3pbo | (-0.678,-7.480,19.625) | 0 | (-0.616,-6.301,20.104) | 1.274 | 1 |
| 820 | 3pbo | (-1.391,-3.619,15.540) | 1 | (-1.876,-3.926,16.709) | 1.302 | 1 |
| 821 | 3pbo | (5.008,-0.473,21.605) | 1 | (2.893,0.399,22.63) | 2.507 | 0 |
| 822 | 3pbo | (-5.660,-3.342,16.980) | 0 | (-5.97,-3.793,17.871) | 1.046 | 1 |
| 823 | 3pbo | (11.766,0.714,20.352) | 1 | (11.471,0.102,20.635) | 0.736 | 1 |
| 824 | 3pbo | (-3.109,0.433,23.132) | 0 | (-4.833,-3.665,21.291) | 4.812 | 0 |
| 825 | 3pbo | (13.853,0.642,10.469) | 0 | (12.3,0.064,11.487) | 1.945 | 1 |
| 826 | 3pbo | (13.240,1.804,12.855) | 0 | (12.368,0.25,12.467) | 1.824 | 1 |
| 827 | 3pbo | (3.978,7.376,9.370) | 0 | (6.831,6.633,12.013) | 3.959 | 0 |
| 828 | 3pbo | (11.039,1.104,14.200) | 0 | (12.368,0.25,12.467) | 2.345 | 0 |
| 829 | 3pbo | (9.008,1.463,17.396) | 0 | (9.21,1.341,17.354) | 0.24 | 1 |
| 830 | 3pbo | (11.773,-3.733,8.364) | 0 | (11.582,-2.942,8.706) | 0.883 | 1 |
| 831 | 3pbo | (-2.561,-3.902,18.688) | 1 | (-2.806,-3.949,17.075) | 1.632 | 1 |
| 832 | 3pbo | (12.103,-0.396,16.421) | 0 | (13.158,-0.364,17.265) | 1.351 | 1 |
| 833 | 3pbo | (11.225,-5.655,6.761) | 0 | (10.625,-5.35,8.129) | 1.525 | 1 |
| 834 | 3pbs | (-5.320,5.908,-16.981) | 0 | (-6.355,5.772,-17.837) | 1.35 | 1 |
| 835 | 3pbs | (-1.098,5.702,-15.358) | 1 | (-1.569,8.428,-19.427) | 4.92 | 0 |
| 836 | 3pbs | (8.403,7.194,-18.452) | 0 | (8.441,7.396,-18.717) | 0.335 | 1 |
| 837 | 3pbs | (5.878,3.140,-21.364) | 1 | (6.027,3.931,-21.071) | 0.857 | 1 |
| 838 | 3pbs | (10.531,7.897,-7.324) | 0 | (10.746,8.265,-7.804) | 0.642 | 1 |
| 839 | 3pbs | (2.880,2.101,-23.812) | 0 | (2.902,1.919,-23.813) | 0.183 | 1 |
| 840 | 3pbs | (14.512,4.110,-6.605) | 0 | (12.912,2.673,-10.805) | 4.719 | 0 |
| 841 | 3pbs | (11.992,1.824,-20.599) | 1 | (11.561,3.532,-18.577) | 2.682 | 0 |
| 842 | 3pbs | (3.268,-4.870,-10.347) | 0 | (0.599,-2.969,-6.394) | 5.135 | 0 |
| 843 | 3pbs | (13.980,2.582,-10.547) | 0 | (12.912,2.673,-10.805) | 1.102 | 1 |
| 844 | 3pbs | (-3.724,4.021,-15.883) | 0 | (-5.956,4.662,-16.39) | 2.377 | 0 |
| 845 | 3pbs | (7.323,-0.077,-18.939) | 0 | (8.009,0.31,-18.737) | 0.813 | 1 |
| 846 | 3pd9 | (57.036,21.963,45.048) | 1 | (56.863,21.931,45.045) | 0.176 | 1 |
| 847 | 3pd9 | (55.635,29.792,48.503) | 1 | (54.856,29.698,48.49) | 0.785 | 1 |
| 848 | 3pd9 | (53.672,23.036,35.962) | 1 | (53.47,23.937,36.993) | 1.384 | 1 |
| 849 | 3pd9 | (53.101,25.620,44.272) | 0 | (52.989,25.694,44.569) | 0.326 | 1 |
| 850 | 3pd9 | (54.773,21.037,37.312) | 1 | (53.272,22.598,37.907) | 2.246 | 0 |
| 851 | 3pd9 | (53.559,26.543,48.005) | 1 | (53.757,27.138,48.385) | 0.733 | 1 |
| 852 | 3pd9 | (53.468,30.706,40.398) | 1 | (53.654,29.029,40.102) | 1.713 | 1 |
| 853 | 3pd9 | (55.879,33.704,45.052) | 1 | (55.774,32.921,45.384) | 0.857 | 1 |
| 854 | 3ph4 | (-2.913,25.229,35.468) | 1 | (-3.331,25.224,36.967) | 1.556 | 1 |
| 855 | 3ph4 | (-7.414,27.918,39.500) | 1 | (-7.249,27.732,39.545) | 0.253 | 1 |
| 856 | 3ph4 | (1.122,29.550,41.778) | 1 | (0.023,35.56,43.697) | 6.404 | 0 |
| 857 | 3ph4 | (-5.507,25.114,42.953) | 1 | (-5.622,25.056,42.697) | 0.287 | 1 |
| 858 | 3ph4 | (-3.956,27.020,33.338) | 1 | (-3.757,27.118,32.989) | 0.414 | 1 |
| 859 | 3ph4 | (-5.404,26.340,40.358) | 1 | (-5.035,25.152,39.598) | 1.458 | 1 |
| 860 | 3ph4 | (-4.478,20.561,34.826) | 1 | (-4.294,20.727,34.987) | 0.296 | 1 |
| 861 | 3ph4 | (-5.765,18.685,44.633) | 1 | (-6.141,18.228,44.424) | 0.628 | 1 |
| 862 | 3ph4 | (0.373,18.594,32.409) | 1 | (0.328,18.188,32.466) | 0.412 | 1 |
| 863 | 3ph4 | (1.076,19.878,37.230) | 1 | (0.328,18.188,32.466) | 5.110 | 0 |
| 864 | 3ph7 | (31.576,18.611,-13.486) | 1 | (31.585,18.55,-13.497) | 0.063 | 1 |
| 865 | 3ph7 | (30.177,16.735,-15.985) | 0 | (31.87,16.565,-15.752) | 1.717 | 1 |
| 866 | 3ph7 | (39.267,13.765,-14.650) | 1 | (39.122,13.765,-14.716) | 0.159 | 1 |
| 867 | 3ph7 | (23.675,7.089,-8.662) | 1 | (23.962,7.053,-9.011) | 0.453 | 1 |
| 868 | 3qvk | (17.157,17.120,8.692) | 1 | (16.751,16.789,9.603) | 1.051 | 1 |
| 869 | 3qvk | (23.521,17.547,10.880) | 1 | (23.486,17.481,11.11) | 0.242 | 1 |
| 870 | 3qvk | (22.011,8.303,11.145) | 1 | (22.993,8.418,12.336) | 1.548 | 1 |
| 871 | 3qvk | (14.008,12.726,9.786) | 0 | (13.831,12.41,9.387) | 0.539 | 1 |
| 872 | 3qvk | (14.126,18.228,11.220) | 1 | (14.099,18.197,11.257) | 0.055 | 1 |
| 873 | 3qvk | (18.936,22.011,18.974) | 1 | (18.923,22.089,18.871) | 0.130 | 1 |
| 874 | 3qvk | (13.654,12.345,12.442) | 1 | (13.645,12.353,12.917) | 0.475 | 1 |
| 875 | 3rxj | (-8.180,-18.502,13.792) | 1 | (-8.098,-19.127,12.571) | 1.374 | 1 |
| 876 | 3rxj | (1.749,-20.091,18.146) | 0 | (2.348,-20.307,20.713) | 2.645 | 0 |
| 877 | 3rxj | (0.586,-18.305,16.550) | 0 | (1.977,-17.8,13.415) | 3.467 | 0 |
| 878 | 3rxj | (-5.318,-17.956,15.355) | 0 | (-8.098,-19.127,12.571) | 4.105 | 0 |
| 879 | 3rxj | (-3.897,-8.781,23.664) | 1 | (-4.079,-9.033,24.021) | 0.473 | 1 |
| 880 | 3rxj | (-1.893,-7.922,20.322) | 1 | (-2.339,-7.513,20.358) | 0.606 | 1 |
| 881 | 3rxj | (-4.906,-11.420,24.232) | 1 | (-4.85,-11.628,24.354) | 0.248 | 1 |
| 882 | 3rxj | (2.063,-17.858,14.116) | 0 | (1.977,-17.8,13.415) | 0.709 | 1 |
| 883 | 3rxj | (-4.749,-15.139,22.977) | 1 | (-4.304,-12.456,24.481) | 3.108 | 0 |
| 884 | 3rxj | (-2.381,-20.081,18.618) | 0 | (0.782,-19.845,20.658) | 3.771 | 0 |
| 885 | 3rxj | (-2.146,-16.611,9.918) | 0 | (1.873,-17.482,12.472) | 4.841 | 0 |
| 886 | 3rxj | (3.643,-19.703,12.592) | 0 | (1.977,-17.8,13.415) | 2.660 | 0 |
| 887 | 3rxj | (0.778,-11.570,17.198) | 1 | (-1.513,-7.669,19.817) | 5.227 | 0 |
| 888 | 3shc | (6.807,8.461,-0.081) | 0 | (7.167,6.409,0.376) | 2.133 | 0 |
| 889 | 3shc | (19.050,8.053,0.983) | 0 | (19.669,8.421,3.75) | 2.859 | 0 |
| 890 | 3shc | (22.134,1.516,2.930) | 0 | (23.525,1.037,3.174) | 1.491 | 1 |
| 891 | 3shc | (17.164,9.995,3.850) | 0 | (19.169,9.017,4.378) | 2.292 | 0 |
| 892 | 3shc | (13.021,12.589,3.028) | 0 | (14.85,16.186,7.815) | 6.261 | 0 |
| 893 | 3shc | (22.208,1.270,-0.052) | 0 | (23.663,1.876,0.292) | 1.613 | 1 |
| 894 | 3shc | (19.007,4.731,-9.705) | 0 | (23.663,1.876,0.292) | 11.392 | 0 |
| 895 | 3shc | (17.082,6.503,-9.836) | 0 | (23.663,1.876,0.292) | 12.934 | 0 |
| 896 | 3skh | (29.132,-8.370,6.185) | 1 | (29.205,-8.508,5.525) | 0.678 | 1 |
| 897 | 3skh | (24.964,4.445,-1.448) | 1 | (24.314,3.105,-2.335) | 1.733 | 1 |
| 898 | 3skh | (20.113,0.295,10.809) | 1 | (19.492,0.766,11.037) | 0.812 | 1 |
| 899 | 3skh | (25.874,0.921,8.321) | 1 | (28.087,-1.394,7.31) | 3.358 | 0 |
| 900 | 3skh | (21.259,-2.969,-6.265) | 1 | (21.09,-3.329,-4.803) | 1.515 | 1 |
| 901 | 3skh | (19.519,-3.146,-4.212) | 1 | (21.09,-3.329,-4.803) | 1.688 | 1 |
| 902 | 3skh | (16.804,8.804,5.292) | 1 | (16.508,8.403,5.39) | 0.508 | 1 |
| 903 | 3skh | (22.967,8.463,3.879) | 1 | (23.153,8.454,3.736) | 0.235 | 1 |
| 904 | 3skh | (28.253,-8.218,3.456) | 1 | (28.871,-8.641,4.592) | 1.361 | 1 |
| 905 | 3skh | (21.861,7.782,-3.034) | 0 | (22.575,8.876,-1.916) | 1.719 | 1 |
| 906 | 3skh | (21.183,-3.096,-0.002) | 1 | (20.305,-1.98,0.639) | 1.558 | 1 |
| 907 | 3sli | (9.335,11.255,39.429) | 1 | (9.228,11.299,39.52) | 0.147 | 1 |
| 908 | 3sli | (6.286,11.173,38.278) | 1 | (6.259,10.83,38.258) | 0.345 | 1 |
| 909 | 3sli | (2.840,1.874,33.449) | 1 | (3.536,1.069,34.266) | 1.342 | 1 |
| 910 | 3sli | (8.010,4.405,41.654) | 1 | (7.124,3.267,41.876) | 1.459 | 1 |
| 911 | 3sli | (5.154,-0.005,41.479) | 1 | (5.436,-0.046,41.129) | 0.451 | 1 |
| 912 | 3sli | (5.741,2.781,43.543) | 1 | (6.08,1.172,44.987) | 2.188 | 0 |
| 913 | 3sli | (-2.413,12.089,42.544) | 1 | (-2.952,11.018,40.925) | 2.015 | 0 |
| 914 | 3sli | (-3.181,10.074,40.669) | 0 | (-2.952,11.018,40.925) | 1.005 | 1 |
| 915 | 3sli | (-0.903,8.847,40.943) | 0 | (-1.497,10.462,41.416) | 1.785 | 1 |
| 916 | 3sli | (1.939,3.667,36.523) | 0 | (1.808,3.924,36.203) | 0.431 | 1 |
| 917 | 3sli | (-1.701,1.300,36.092) | 1 | (-1.641,1.793,34.768) | 1.414 | 1 |
| 918 | 3sli | (4.421,4.359,41.438) | 0 | (6.616,3.522,41.053) | 2.381 | 0 |
| 919 | 3sli | (3.478,0.321,38.577) | 0 | (4.088,-1.113,39.538) | 1.831 | 1 |
| 920 | 3sli | (-5.066,8.610,42.005) | 0 | (-2.952,11.018,40.925) | 3.381 | 0 |
| 921 | 3sli | (1.255,10.369,44.303) | 1 | (1.986,10.197,44.116) | 0.774 | 1 |
| 922 | 3sli | (5.023,9.146,42.512) | 1 | (5.099,9.234,42.61) | 0.152 | 1 |
| 923 | 3sli | (-2.181,13.513,40.205) | 0 | (-2.036,13.456,40.359) | 0.219 | 1 |
| 924 | 3smb | (28.895,9.457,12.984) | 1 | (28.905,9.805,13.24) | 0.432 | 1 |
| 925 | 3smb | (43.306,14.014,5.764) | 0 | (39.951,15.468,5.847) | 3.657 | 0 |
| 926 | 3smb | (37.706,12.031,7.755) | 1 | (39.496,14.584,5.956) | 3.600 | 0 |
| 927 | 3smb | (25.356,11.885,8.411) | 1 | (25.64,13.882,12.232) | 4.321 | 0 |
| 928 | 3t1m | (16.829,-14.106,7.537) | 1 | (16.689,-14.135,7.757) | 0.262 | 1 |
| 929 | 3t1m | (9.032,-3.431,1.179) | 1 | (9.098,-3.403,1.581) | 0.408 | 1 |
| 930 | 3t1m | (23.056,-9.427,1.479) | 1 | (23.664,-8.894,1.393) | 0.813 | 1 |
| 931 | 3t1m | (12.224,-5.463,-0.315) | 1 | (10.019,-3.221,1.236) | 3.506 | 0 |
| 932 | 3t1m | (5.422,-9.352,2.108) | 1 | (4.701,-11.854,5.233) | 4.068 | 0 |
| 933 | 3t1m | (22.576,-6.024,-7.423) | 1 | (22.653,-0.018,-6.164) | 6.137 | 0 |
| 934 | 3t1m | (28.199,-4.679,0.399) | 1 | (23.664,-8.894,1.393) | 6.271 | 0 |
| 935 | 3t1m | (13.725,-3.614,-2.718) | 1 | (15.747,-6.318,-4.764) | 3.948 | 0 |
| 936 | 3t1m | (24.090,-12.600,2.927) | 1 | (24.673,-10.161,1.597) | 2.839 | 0 |
| 937 | 3t1m | (15.646,-8.973,1.609) | 1 | (15.747,-6.318,-4.764) | 6.905 | 0 |
| 938 | 3t1m | (8.693,-11.039,2.248) | 1 | (4.701,-11.854,5.233) | 5.051 | 0 |
| 939 | 3t1m | (6.439,-9.066,-0.712) | 0 | (8.486,-3.595,0.814) | 6.037 | 0 |
| 940 | 3t1m | (15.736,-10.787,3.693) | 1 | (15.755,-13.901,8.027) | 5.337 | 0 |
| 941 | 3t1m | (20.750,-18.341,6.343) | 1 | (17.943,-16.919,6.518) | 3.152 | 0 |
| 942 | 3t1m | (23.577,-6.713,-2.323) | 0 | (23.664,-8.894,1.393) | 4.310 | 0 |
| 943 | 3t1m | (6.607,-12.612,3.780) | 0 | (5.499,-13.101,5.922) | 2.461 | 0 |
| 944 | 3t1m | (16.241,-7.024,-5.372) | 1 | (16.299,-7.187,-6.031) | 0.681 | 1 |
| 945 | 3t1m | (18.725,-9.178,-4.949) | 0 | (16.299,-7.187,-6.031) | 3.320 | 0 |
| 946 | 3t78 | (-23.816,3.399,-6.666) | 0 | (-19.512,3.736,-3.835) | 5.163 | 0 |
| 947 | 3t78 | (-12.951,1.398,-6.843) | 1 | (-12.713,0.178,-7.431) | 1.375 | 1 |
| 948 | 3t78 | (-12.754,-0.464,-9.304) | 1 | (-13.231,-0.561,-7.861) | 1.523 | 1 |
| 949 | 3t78 | (-22.361,5.447,-7.868) | 1 | (-18.734,5.548,-8.132) | 3.638 | 0 |
| 950 | 3t78 | (-14.253,-6.353,-9.550) | 1 | (-16.022,-6.57,-9.509) | 1.783 | 1 |
| 951 | 3t78 | (-18.419,5.991,-9.154) | 1 | (-18.351,6.347,-9.503) | 0.503 | 1 |
| 952 | 3t78 | (-13.638,4.025,-9.280) | 0 | (-12.05,1.328,-11.537) | 3.859 | 0 |
| 953 | 3t78 | (-17.134,0.337,-7.249) | 0 | (-16.974,0.774,-7.255) | 0.465 | 1 |
| 954 | 3t78 | (-18.084,4.051,-3.630) | 0 | (-17.205,4.288,-3.679) | 0.912 | 1 |
| 955 | 3upn | (18.465,-67.480,12.876) | 0 | (18.152,-67.289,12.943) | 0.373 | 1 |
| 956 | 3upn | (12.605,-62.768,10.883) | 0 | (12.67,-63.292,11.085) | 0.565 | 1 |
| 957 | 3uwz | (-7.511,22.060,22.295) | 0 | (-5.864,26.085,20.293) | 4.788 | 0 |
| 958 | 3uwz | (-5.645,25.984,20.597) | 1 | (-5.864,26.085,20.293) | 0.388 | 1 |
| 959 | 3uwz | (-0.347,18.652,21.233) | 1 | (0.851,20.2,24.364) | 3.693 | 0 |
| 960 | 3uwz | (-7.731,17.205,20.414) | 0 | (-5.284,14.859,21.292) | 3.502 | 0 |
| 961 | 3uwz | (-1.753,21.355,25.634) | 1 | (-1.603,21.538,25.747) | 0.262 | 1 |
| 962 | 3uwz | (-2.107,20.217,19.794) | 1 | (-3.229,15.947,20.796) | 4.527 | 0 |
| 963 | 3uwz | (-4.035,15.453,27.968) | 1 | (-3.455,15.219,28.743) | 0.996 | 1 |
| 964 | 3uwz | (-3.820,24.135,26.835) | 1 | (-3.885,24.21,26.818) | 0.101 | 1 |
| 965 | 3uwz | (-7.629,24.630,21.566) | 1 | (-5.864,26.085,20.293) | 2.618 | 0 |
| 966 | 3uwz | (-9.724,25.195,19.873) | 1 | (-9.536,25.968,17.091) | 2.894 | 0 |
| 967 | 3vah | (-13.020,-9.565,23.856) | 0 | (-14.11,-13.454,23.04) | 4.12 | 0 |
| 968 | 3vah | (-7.233,-17.224,15.534) | 1 | (-9.011,-13.057,15.496) | 4.531 | 0 |
| 969 | 3vah | (-12.034,-12.405,14.781) | 1 | (-10.596,-12.914,13.255) | 2.158 | 0 |
| 970 | 3vah | (-1.902,-13.709,13.720) | 1 | (-1.614,-13.996,13.809) | 0.416 | 1 |
| 971 | 3vah | (-9.474,-6.329,18.153) | 1 | (-8.083,-8.954,17.8) | 2.992 | 0 |
| 972 | 3vah | (1.692,-12.857,19.187) | 0 | (2.149,-13.117,19.262) | 0.531 | 1 |
| 973 | 3vah | (-5.090,-9.465,26.235) | 1 | (-1.712,-10.107,24.848) | 3.708 | 0 |
| 974 | 3vah | (-11.159,-10.172,19.467) | 0 | (-7.995,-10.299,18.723) | 3.253 | 0 |
| 975 | 3vah | (-2.802,-5.610,26.834) | 1 | (0.472,-4.933,24.946) | 3.840 | 0 |
| 976 | 3vah | (-10.539,-9.878,22.066) | 0 | (-8.7,-10.581,22.94) | 2.154 | 0 |
| 977 | 3vah | (-5.325,-7.020,27.436) | 0 | (-1.645,-9.174,24.495) | 5.180 | 0 |
| 978 | 3voa | (2.163,-6.682,25.468) | 1 | (1.982,-7.143,25.746) | 0.568 | 1 |
| 979 | 3voa | (-0.461,-10.828,20.048) | 1 | (-1.109,-6.055,20.533) | 4.841 | 0 |
| 980 | 3voa | (0.765,-2.824,16.752) | 1 | (-0.189,-4.739,20.833) | 4.608 | 0 |
| 981 | 3voa | (9.546,-6.149,27.073) | 1 | (9.031,-7.099,27.353) | 1.116 | 1 |
| 982 | 3voa | (1.723,-11.316,26.341) | 1 | (1.569,-11.427,26.49) | 0.241 | 1 |
| 983 | 3voa | (9.551,-4.827,24.730) | 0 | (8.227,-2.872,24.457) | 2.377 | 0 |
| 984 | 3voa | (7.758,-8.352,28.857) | 0 | (8.335,-7.142,28.83) | 1.341 | 1 |
| 985 | 3voa | (7.562,-10.875,29.181) | 1 | (7.372,-12.599,28.047) | 2.072 | 0 |
| 986 | 3voa | (8.026,-2.537,25.338) | 0 | (8.027,-2.466,25.349) | 0.072 | 1 |
| 987 | 3voa | (11.205,-9.094,27.590) | 0 | (9.031,-7.099,27.353) | 2.960 | 0 |
| 988 | 3voa | (-0.802,-1.535,18.854) | 0 | (-1.94,-0.042,20.422) | 2.446 | 0 |
| 989 | 3voa | (8.465,1.394,18.232) | 0 | (8.673,1.005,18.913) | 0.811 | 1 |
| 990 | 3voa | (-0.668,-3.736,22.328) | 0 | (-0.849,-3.522,22.547) | 0.356 | 1 |
| 991 | 3voa | (1.064,-3.348,20.235) | 1 | (-0.189,-4.739,20.833) | 1.965 | 1 |
| 992 | 3voa | (0.349,-5.447,20.846) | 1 | (-0.273,-5.726,20.972) | 0.693 | 1 |
| 993 | 3w3d | (-16.920,17.272,41.657) | 1 | (-16.921,16.808,41.822) | 0.492 | 1 |
| 994 | 3w3d | (-19.058,16.160,39.494) | 1 | (-18.777,15.653,38.977) | 0.777 | 1 |
| 995 | 3w3d | (-17.881,13.859,41.705) | 1 | (-16.644,13.802,42.532) | 1.489 | 1 |
| 996 | 3w3d | (-14.234,13.050,29.410) | 1 | (-14.223,12.196,29.932) | 1.001 | 1 |
| 997 | 3w3d | (-15.219,11.506,40.042) | 1 | (-15.254,11.458,40.385) | 0.348 | 1 |
| 998 | 3w3d | (-17.193,13.632,38.660) | 1 | (-17.274,13.391,38.444) | 0.334 | 1 |
| 999 | 3w3d | (-13.648,20.442,31.106) | 1 | (-13.137,20.04,31.094) | 0.650 | 1 |
| 1000 | 3w3d | (-10.489,22.584,33.942) | 1 | (-12.556,20.427,33.275) | 3.061 | 0 |
| 1001 | 3w3d | (-22.486,15.186,38.620) | 1 | (-22.338,15.084,38.115) | 0.536 | 1 |
| 1002 | 3w3d | (-12.071,19.554,33.467) | 1 | (-12.335,18.809,33.316) | 0.805 | 1 |
| 1003 | 3w3d | (-22.222,18.192,39.448) | 1 | (-22.041,17.749,39.333) | 0.492 | 1 |
| 1004 | 3w3d | (-7.387,18.031,30.176) | 0 | (-5.603,16.049,29.138) | 2.862 | 0 |
| 1005 | 3w3d | (-22.941,13.086,25.745) | 0 | (-24.3,11.225,26.823) | 2.544 | 0 |
| 1006 | 3w3d | (-22.206,17.172,25.906) | 0 | (-22.499,16.738,27.515) | 1.692 | 1 |
| 1007 | 3w3d | (-15.899,10.451,42.830) | 1 | (-16.915,10.67,43.647) | 1.322 | 1 |
| 1008 | 3w3d | (-15.016,15.429,42.078) | 1 | (-15.168,15.152,42.857) | 0.841 | 1 |
| 1009 | 3w3d | (-19.980,12.169,42.166) | 0 | (-21.198,12.322,41.988) | 1.240 | 1 |
| 1010 | 3zya | (9.826,-17.675,-41.350) | 0 | (9.621,-17.617,-41.617) | 0.342 | 1 |
| 1011 | 3zya | (10.557,-9.688,-35.924) | 0 | (11.325,-10.585,-36.289) | 1.236 | 1 |
| 1012 | 3zya | (6.542,-15.320,-38.209) | 0 | (7.812,-12.425,-40.774) | 4.071 | 0 |
| 1013 | 3zya | (7.514,-8.566,-30.001) | 0 | (10.507,-5.547,-32.207) | 4.789 | 0 |
| 1014 | 3zya | (14.069,-10.901,-29.977) | 1 | (14.301,-8.929,-28.861) | 2.278 | 0 |
| 1015 | 3zya | (4.644,-13.679,-34.295) | 0 | (4.003,-13.523,-34.087) | 0.692 | 1 |
| 1016 | 3zya | (9.967,-20.664,-39.733) | 0 | (9.872,-20.379,-41.554) | 1.846 | 1 |
| 1017 | 3zya | (18.147,-6.630,-29.048) | 0 | (20.555,-5.217,-27.089) | 3.411 | 0 |
| 1018 | 3zya | (18.024,-10.563,-25.388) | 0 | (18.252,-10.667,-26.072) | 0.728 | 1 |
| 1019 | 3zya | (15.984,-20.719,-27.865) | 1 | (19.005,-21.443,-26.477) | 3.403 | 0 |
| 1020 | 3zya | (11.869,-25.371,-34.373) | 0 | (10.046,-24.998,-34.269) | 1.864 | 1 |
| 1021 | 3zya | (6.433,-20.754,-36.361) | 1 | (9.746,-23.66,-33.382) | 5.319 | 0 |
| 1022 | 3zya | (4.107,-18.119,-34.686) | 1 | (3.895,-14.45,-33.727) | 3.798 | 0 |
| 1023 | 3zya | (16.600,-12.782,-25.304) | 0 | (17.466,-11.285,-26.049) | 1.883 | 1 |
| 1024 | 4adu | (26.914,13.621,-22.261) | 1 | (26.602,12.918,-22.651) | 0.862 | 1 |
| 1025 | 4adu | (27.231,11.705,-29.084) | 1 | (27.272,11.454,-28.716) | 0.447 | 1 |
| 1026 | 4adu | (31.713,12.427,-26.928) | 1 | (31.628,12.365,-27.023) | 0.142 | 1 |
| 1027 | 4adu | (29.613,12.733,-29.937) | 1 | (29.456,12.043,-29.65) | 0.764 | 1 |
| 1028 | 4aqh | (-27.331,2.857,3.319) | 1 | (-27.257,3.161,2.557) | 0.824 | 1 |
| 1029 | 4aqh | (-39.183,4.058,2.627) | 0 | (-39.358,4.459,2.187) | 0.621 | 1 |
| 1030 | 4aqh | (-38.461,2.330,6.087) | 0 | (-38.831,4.65,3.015) | 3.867 | 0 |
| 1031 | 4b6p | (25.500,47.576,9.909) | 0 | (25.882,47.577,9.846) | 0.387 | 1 |
| 1032 | 4b6p | (23.070,52.483,4.658) | 1 | (27.629,49.238,5.316) | 5.634 | 0 |
| 1033 | 4b6p | (33.732,51.476,2.318) | 1 | (33.806,51.455,1.669) | 0.654 | 1 |
| 1034 | 4b6p | (23.838,38.319,6.447) | 1 | (23.566,38.517,6.583) | 0.363 | 1 |
| 1035 | 4b6p | (38.040,41.734,5.504) | 1 | (38.524,43.251,4.664) | 1.800 | 1 |
| 1036 | 4b6p | (32.035,48.788,11.402) | 1 | (30.917,47.225,9.773) | 2.519 | 0 |
| 1037 | 4b6p | (29.174,36.023,12.175) | 1 | (29.164,35.758,12.336) | 0.310 | 1 |
| 1038 | 4b6r | (19.888,28.818,0.161) | 1 | (20.672,28.18,0.07) | 1.015 | 1 |
| 1039 | 4b6r | (16.197,28.795,-5.175) | 1 | (16.15,28.828,-5.023) | 0.162 | 1 |
| 1040 | 4b6r | (20.251,20.900,2.622) | 1 | (20.739,20.809,3.443) | 0.959 | 1 |
| 1041 | 4b6r | (6.948,19.106,1.273) | 1 | (7.258,19.069,1.264) | 0.312 | 1 |
| 1042 | 4eys | (53.078,-2.652,6.539) | 1 | (52.543,-2.47,6.324) | 0.605 | 1 |
| 1043 | 4eys | (43.932,1.206,15.308) | 0 | (43.856,1.743,15.954) | 0.843 | 1 |
| 1044 | 4eys | (43.011,1.643,18.101) | 1 | (42.744,1.58,18.557) | 0.532 | 1 |
| 1045 | 4eys | (44.176,7.167,11.502) | 0 | (44.644,7.307,12.132) | 0.797 | 1 |
| 1046 | 4eys | (46.042,-7.444,10.337) | 1 | (46.301,-7.248,10.236) | 0.340 | 1 |
| 1047 | 4eys | (43.180,-7.404,6.554) | 1 | (43.322,-7.396,6.611) | 0.153 | 1 |
| 1048 | 4eys | (37.613,6.956,9.129) | 0 | (39.844,2.603,9.303) | 4.895 | 0 |
| 1049 | 4eys | (36.208,-2.164,3.107) | 0 | (39.637,1.795,8.751) | 7.700 | 0 |
| 1050 | 4eys | (46.029,-3.366,9.046) | 1 | (46.035,-3.636,9.281) | 0.358 | 1 |
| 1051 | 4eys | (40.558,-6.267,7.366) | 0 | (42.58,-6.747,6.781) | 2.159 | 0 |
| 1052 | 4eys | (45.028,6.937,14.149) | 1 | (46.432,8.4,14.629) | 2.084 | 0 |
| 1053 | 4eys | (50.421,-2.715,7.632) | 1 | (49.441,-2.715,8.158) | 1.112 | 1 |
| 1054 | 4eys | (43.483,-5.604,8.475) | 1 | (43.744,-6.607,8.819) | 1.092 | 1 |
| 1055 | 4eys | (40.834,5.727,11.205) | 0 | (40.666,3.048,8.948) | 3.507 | 0 |
| 1056 | 4eys | (43.143,4.110,8.705) | 0 | (43.684,4.372,8.557) | 0.619 | 1 |
| 1057 | 4eys | (40.170,3.393,7.205) | 0 | (40.666,3.048,8.948) | 1.845 | 1 |
| 1058 | 4f0f | (-12.057,17.196,-15.688) | 1 | / | / | / |
| 1059 | 4f0f | (-6.526,14.475,-22.069) | 1 | / | / | / |
| 1060 | 4f0f | (-3.076,13.930,-22.413) | 1 | / | / | / |
| 1061 | 4f0f | (-12.721,24.061,-18.654) | 1 | / | / | / |
| 1062 | 4f0f | (-6.722,16.926,-21.585) | 1 | / | / | / |
| 1063 | 4f0f | (2.722,19.333,-20.084) | 0 | / | / | / |
| 1064 | 4f0f | (-9.970,21.927,-20.647) | 1 | / | / | / |
| 1065 | 4f0f | (-6.300,23.014,-10.467) | 0 | / | / | / |
| 1066 | 4f0f | (-2.968,16.065,-21.015) | 0 | / | / | / |
| 1067 | 4f0f | (-8.117,25.364,-25.149) | 1 | / | / | / |
| 1068 | 4f0f | (-7.963,23.035,-25.276) | 0 | / | / | / |
| 1069 | 4f0f | (-4.409,18.621,-25.892) | 0 | / | / | / |
| 1070 | 4f0f | (-3.799,28.401,-13.845) | 1 | / | / | / |
| 1071 | 4f0f | (-15.222,24.112,-17.933) | 1 | / | / | / |
| 1072 | 4g8y | (37.881,0.861,14.966) | 1 | (38.174,1.004,15.021) | 0.331 | 1 |
| 1073 | 4g8y | (36.817,-1.703,14.884) | 0 | (36.557,-1.265,14.613) | 0.577 | 1 |
| 1074 | 4g8y | (34.494,8.189,21.766) | 0 | (33.881,7.361,24.982) | 3.377 | 0 |
| 1075 | 4g8y | (34.638,-5.421,23.691) | 1 | (28.507,-8.313,21.835) | 7.028 | 0 |
| 1076 | 4g8y | (38.891,-3.394,16.005) | 0 | (37.914,-3.093,17.034) | 1.451 | 1 |
| 1077 | 4g8y | (34.750,-8.389,22.040) | 1 | (35.61,-12.509,23.226) | 4.373 | 0 |
| 1078 | 4g8y | (37.726,0.481,20.145) | 0 | (36.211,0.163,19.438) | 1.702 | 1 |
| 1079 | 4g8y | (41.296,1.317,26.417) | 1 | (41.242,1.509,26.371) | 0.205 | 1 |
| 1080 | 4g8y | (32.469,7.092,24.929) | 1 | (32.297,6.448,24.739) | 0.693 | 1 |
| 1081 | 4g8y | (40.096,1.099,16.419) | 0 | (38.386,0.091,15.37) | 2.245 | 0 |
| 1082 | 4g8y | (38.097,2.704,18.656) | 0 | (36.688,0.171,17.876) | 3.002 | 0 |
| 1083 | 4g8y | (37.646,-7.735,15.339) | 0 | (34.148,-11.044,15.826) | 4.840 | 0 |
| 1084 | 4g8y | (40.311,0.570,21.827) | 0 | (41.524,1.726,25.437) | 3.980 | 0 |
| 1085 | 4g8y | (42.467,-2.524,14.301) | 0 | (38.386,0.091,15.37) | 4.963 | 0 |
| 1086 | 4g8y | (35.795,-0.834,17.282) | 0 | (34.668,-1.418,16.977) | 1.305 | 1 |
| 1087 | 4g8y | (39.281,7.661,23.691) | 0 | (38.298,8.839,24.46) | 1.716 | 1 |
| 1088 | 4g8y | (36.785,-9.469,20.968) | 0 | (35.61,-12.509,23.226) | 3.965 | 0 |
| 1089 | 4g8y | (39.798,-7.739,17.639) | 0 | (37.914,-3.093,17.034) | 5.050 | 0 |
| 1090 | 4g8y | (42.307,-7.618,20.913) | 0 | (37.914,-3.093,17.034) | 7.404 | 0 |
| 1091 | 4g8y | (33.061,10.006,19.463) | 0 | (32.572,9.624,24.565) | 5.140 | 0 |
| 1092 | 4g8y | (34.861,10.744,19.580) | 0 | (32.572,9.624,24.565) | 5.599 | 0 |
| 1093 | 4sli | (9.271,11.244,39.438) | 1 | (4.025,2.44,35.849) | 10.859 | 0 |
| 1094 | 4sli | (1.400,0.778,31.396) | 1 | (1.543,1.138,31.667) | 0.473 | 1 |
| 1095 | 4sli | (6.255,11.011,38.160) | 1 | (-1.304,14.383,40.464) | 8.592 | 0 |
| 1096 | 4sli | (2.728,1.815,33.619) | 1 | (1.638,1.738,32.461) | 1.592 | 1 |
| 1097 | 4sli | (8.075,4.428,41.719) | 1 | (4.025,2.44,35.849) | 7.403 | 0 |
| 1098 | 4sli | (5.177,-0.188,41.539) | 1 | (2.297,0.397,36.891) | 5.499 | 0 |
| 1099 | 4sli | (-1.201,0.650,31.056) | 1 | (-1.261,0.467,30.91) | 0.242 | 1 |
| 1100 | 4sli | (-2.138,12.002,42.824) | 1 | (-2.363,10.414,41.632) | 1.998 | 1 |
| 1101 | 4sli | (1.828,-1.695,38.123) | 1 | (1.801,-3.872,38.484) | 2.207 | 0 |
| 1102 | 4sli | (6.027,9.292,42.695) | 1 | (-2.363,10.414,41.632) | 8.531 | 0 |
| 1103 | 4sli | (-1.838,1.154,36.071) | 1 | (-1.503,0.86,34.635) | 1.504 | 1 |
| 1104 | 4sli | (6.074,2.621,43.848) | 1 | (2.297,0.397,36.891) | 8.223 | 0 |
| 1105 | 4sli | (1.945,10.781,44.335) | 1 | (-2.363,10.414,41.632) | 5.099 | 0 |
| 1106 | 4sli | (-0.751,4.329,35.616) | 0 | (-2.391,1.302,34.512) | 3.615 | 0 |
| 1107 | 4sli | (2.209,-2.379,40.602) | 1 | (1.313,-3.994,39.348) | 2.232 | 0 |
| 1108 | 4sli | (3.118,0.684,37.959) | 1 | (2.297,0.397,36.891) | 1.377 | 1 |
| 1109 | 4sli | (2.820,1.227,40.640) | 1 | (2.297,0.397,36.891) | 3.875 | 0 |
| 1110 | 4sli | (0.402,1.913,34.966) | 0 | (-0.959,0.968,33.803) | 2.024 | 0 |
| 1111 | 4sli | (-3.100,10.406,40.637) | 1 | (-2.598,10.702,40.703) | 0.586 | 1 |
| 1112 | 4thi | (17.453,26.756,11.189) | 1 | (17.373,26.918,11.075) | 0.214 | 1 |
| 1113 | 4thi | (11.157,31.482,9.678) | 0 | (10.826,30.867,9.649) | 0.699 | 1 |
| 1114 | 4thi | (16.958,23.500,9.641) | 0 | (17.106,23.371,9.561) | 0.212 | 1 |
| 1115 | 4thi | (12.490,23.579,-2.429) | 1 | (12.036,23.735,-2.536) | 0.492 | 1 |
| 1116 | 4thi | (12.856,25.704,3.153) | 0 | (14.627,27.11,1.231) | 2.968 | 0 |
| 1117 | 4thi | (13.835,32.701,9.718) | 0 | (14.05,32.713,9.192) | 0.568 | 1 |
| 1118 | 4thi | (16.041,35.242,5.601) | 1 | (16.759,34.398,6.592) | 1.487 | 1 |
| 1119 | 4thi | (11.289,31.824,1.227) | 0 | (10.826,31.616,1.519) | 0.586 | 1 |
| 1120 | 4thi | (13.194,34.512,7.385) | 0 | (13.689,33.8,6.567) | 1.192 | 1 |
| 1121 | 4thi | (20.011,26.700,4.395) | 0 | (18.894,26.375,3.912) | 1.260 | 1 |
| 1122 | 6tim | (45.696,16.644,-15.753) | 1 | (45.732,16.873,-15.83) | 0.244 | 1 |
| 1123 | 6tim | (47.648,20.193,-14.515) | 1 | (49.822,19.272,-15.863) | 2.719 | 0 |
| 1124 | 6tim | (50.348,21.023,-7.027) | 1 | (50.449,20.519,-7.121) | 0.523 | 1 |
| 1125 | 6tim | (47.286,20.063,-10.686) | 1 | (44.956,23.026,-10.59) | 3.771 | 0 |
| 1126 | 6tim | (48.908,13.354,-4.248) | 1 | (49.35,13.324,-4.083) | 0.473 | 1 |
| 1127 | 6tim | (46.669,12.788,-13.844) | 1 | (46.652,13.024,-13.449) | 0.460 | 1 |
